# The Carbonyl⋅⋅⋅Tellurazole Chalcogen Bond as a Molecular Recognition Unit: From Model Studies to Supramolecular Organic Frameworks

**DOI:** 10.1002/anie.202005374

**Published:** 2020-07-29

**Authors:** Saber Mehrparvar, Christoph Wölper, Rolf Gleiter, Gebhard Haberhauer

**Affiliations:** ^1^ Institut für Organische Chemie Universität Duisburg-Essen Universitätsstraße 7 45117 Essen Germany; ^2^ Organisch-Chemisches Institut Universität Heidelberg Im Neuenheimer Feld 270 69120 Heidelberg Germany

**Keywords:** chalcogen bonding bonds, DFT calculations, self-assembly, supramolecular organic frameworks

## Abstract

In the last years, chalcogen bonding, the noncovalent interaction involving chalcogen centers, has emerged as interesting alternative to the ubiquitous hydrogen bonding in many research areas. Here, we could show by means of high‐level quantum chemical calculations that the carbonyl⋅⋅⋅tellurazole chalcogen bond is at least as strong as conventional hydrogen bonds. Using the carbonyl⋅⋅⋅tellurazole binding motif, we were able to design complex supramolecular networks in solid phase starting from tellurazole‐substituted cyclic peptides. X‐ray analyses reveal that the rigid structure of the cyclic peptides is caused by hydrogen bonds, whereas the supramolecular network is held together by chalcogen bonding. The type of the supramolecular network depends on peptide used; both linear wires and a honeycomb‐like supramolecular organic framework (SOF) were observed. The unique structure of the SOF shows two channels filled with different types of solvent mixtures that are either locked or freely movable.

## Graphical abstract

**Quantum chemical calculations** show that the carbonyl⋅⋅⋅tellurazole chalcogen bond is at least as strong as conventional hydrogen bonds. This type of chalcogen bond was used in combination with hydrogen bonds to design a honeycomb‐like supramolecular organic framework showing two types of channels.

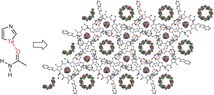

## Introduction

The concept of the chalcogen bond^1^ has been the subject of intensive research over the past years.^2^, ^3^, ^4^ It has been shown that chalcogen bonds can be used in several different fields ranging from crystal engineering^3^, ^5^, ^6^ through molecular recognition in solution^7^, ^8^, ^9^, ^10^, ^11^, ^12^, ^13^ up to catalysis.^12^, ^14^, ^15^, ^16^, ^17^, ^18^, ^19^, ^20^, ^21^ Systematic investigations of the chalcogen–chalcogen interactions^22^, ^23^, ^24^, ^25^, ^26^, ^27^, ^28^, ^29^, ^30^, ^31^ have demonstrated that chalcogen bonds strongly depend on the size of the chalcogen atom and the substituents on the acceptor chalcogen atom.^3^, ^32^, ^33^ Tellurium compounds^34^ with an electron‐withdrawing substituent show the highest bond strengths.^32^ In contrast, it was found for dimethylchalcogenides as test systems that the nature of the donor chalcogen atom plays no significant role for the strength of the chalcogen–chalcogen interaction.^32^ Accordingly, particularly strong chalcogen bonds are found in electron‐poor tellurium compounds such as isotellurazole oxides^13^, ^35^ and telluradiazole.^10^, ^36^, ^37^ The dimers und oligomers formed by these tellurium‐containing compounds can be found not only in the solid state but also in solution.^13^, ^36^ In addition to isotellurazole oxides and telluradiazole, tellurazoles have been recently used as promising building blocks for the design of strong chalcogen bonds.^38^ The advantage of 1,3‐benzotellurazoles is that they are both easily accessible and relatively stable.^39^ It could be shown that intermolecular interactions between the nitrogen and the tellurium atoms in these molecules result in the formation of supramolecular wires in solid state.^38^

Here we describe the design of new molecular recognition units based on chalcogen bonds. In order to build up complex supramolecular systems, the molecular recognition units consist of two different types of functional groups. One unit is the carbonyl group of an amide and the second unit represents chalcogenazoles. By means of quantum chemical calculations we could show that the strength of a chalcogen bond between the carbonyl group of an amide and the tellurium atom of a chalcogenazole is roughly the strength of a hydrogen bond between the amide group and the nitrogen atom of a chalcogenazole. Furthermore, we demonstrate that these molecular recognition units (carbonyl⋅⋅⋅tellurazole) can be used to design complex supramolecular networks in a solid phase. Besides two‐dimensional wires, the formation of a honeycomb‐like supramolecular organic framework (SOF)^40^, ^41^ was observed in the solid state.

## Results and Discussion

### Intermolecular C=O⋅⋅⋅E Chalcogen Bond vs. Hydrogen Bond

**Scheme 1:**
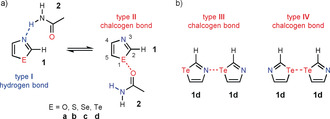
a) Interaction between a 1,3‐chalcogenazole (**1**) and acetamide (**2**) via a N−H⋅⋅⋅N hydrogen bond (type **I**) and a C=O⋅⋅⋅E chalcogen bond (type **II**). In the case of the studied C=O⋅⋅⋅E chalcogen bond, the lone pair of the carbonyl group interacts with the σ* orbital of the E−C(5) bond. b) Dimers of 1,3‐tellurazole (**1 d**) via intermolecular Te⋅⋅⋅N (type **III**) and Te⋅⋅⋅Te (type **IV**) interactions.

In the first step we intended to compare an intermolecular C=O⋅⋅⋅E chalcogen bond with the intermolecular hydrogen bond between a chalcogenazole and an amide by means of quantum chemical calculations. As model systems we used the nonsubstituted chalcogenazoles **1** (E=O, S, Se and Te) and acetamide **2** (see Scheme 1 a). These two model compounds can form two different intermolecular complexes: On the one hand they can interact via a N−H⋅⋅⋅N hydrogen bond between the hydrogen atom of the amide group and the nitrogen atom of the chalcogenazole (type **I**) and on the other hand the formation of a C=O⋅⋅⋅E chalcogen bond formed between the oxygen atom of the carbonyl group of **2** with the chalcogen atom of the chalcogenazole **1** is possible (type **II** in Scheme 1 a). For an additional comparison, two dimers of tellurazole **1 d** were considered in the calculations. The dimers show either a N⋅⋅⋅Te chalcogen bond (type **III**) or a Te⋅⋅⋅Te chalcogen bond (type **IV** in Scheme 1 b). Both types of interaction have already been observed in the solid state. For example, bis(methyltelluro)alkynes show relatively short Te⋅⋅⋅Te contacts of two neighbors giving rise to a zigzag arrangement between the Te centers and forming stacks of molecules.^28^ In the solid state benzo‐1,3‐tellurazoles form wire‐like polymeric structures built up through N⋅⋅⋅Te chalcogen bonds.^38^

In order to optimize the structures of the complexes **1⋅2** (type **I** and type **II**) and the dimers **1 d⋅1 d** (type **III** and type **IV**) the density functional method B3LYP^42^, ^43^, ^44^ and the double‐hybrid density functional approximation B2PLYP^45^ were employed. In the latter, a part of the correlation energy is calculated with second‐order perturbation theory.^45^ In both cases, the dispersion interaction, which is essential for this type of complex,^3^, ^22^, ^32^ is considered using the additional dispersion correction with Becke–Johnson damping^46^ (D3BJ). As the basis set TZVP was employed for the light elements C, H, N, O, S, and Se, whereas aug‐cc‐pVTZ‐PP was used for tellurium. Subsequent frequency analyses show that all structures are minima on the potential energy surface. Furthermore, single‐point calculations on the B2PLYP‐optimized structures were performed using B2PLYP, CCSD^47^ and CCSD(T).^48^ As basis sets TZVP and aug‐cc‐pVTZ‐PP were employed. To determine the solvent effect, single‐point calculations using the SMD^49^ model were conducted with both B2PLYP and CCSD as methods. As solvent, water was applied. The calculated data for the structures of the complexes **1⋅2** (type **I** and type **II**) are summarized in Tables 1 and 2.

**Table 1:** Calculated distances [Å] of the intermolecular N−H⋅⋅⋅N hydrogen bonds and C=O⋅⋅⋅E chalcogen bonds in the complexes **1⋅2** (type **I** and **II**).

E	Type	*d*(NH⋅⋅⋅N)^[a]^	*d*(NH⋅⋅⋅N)^[b]^	Type	*d*(COE)^[a]^	*d*(COE)^[b]^
O	**I**	2.021	2.042			
S	**I**	2.009	2.032	**II**	3.246	3.247
Se	**I**	2.013	2.035	**II**	3.126	3.146
Te	**I**	2.014	2.039	**II**	3.010	2.997

[a] B3LYP‐D3BJ/TZVP,cc‐pVTZ. [b] B2PLYP‐D3/TZVP,cc‐pVTZ.

**Table 2:** Formation energies [kcal mol^−1^] of the complexes **1⋅2** via a N−H⋅⋅⋅N hydrogen bond (type **I**) and C=O⋅⋅⋅E chalcogen bond (type **II**) calculated using different methods.

E	Type	Δ*E* ^[a]^	Δ*E* ^[b]^	Δ*E* ^[c]^	Δ*E* ^[d]^	Δ*E* ^[e]^	Δ*E* ^[f]^	Δ*E* ^[g]^
O	**I**	−10.4	−9.8	−8.8	−8.4	−9.0	‐3.8	‐1.5
S	**I**	−10.4	−9.9	−8.7	−8.5	−9.1	‐3.9	‐1.8
Se	**I**	−10.4	−9.8	−8.6	−8.4	−9.0	‐4.0	‐2.3
Te	**I**	−10.2	−9.6	−8.5	−8.3	−8.9	−3.9	−2.5
								
S	**II**	−5.5	−5.1	−4.5	−4.3	−4.7	−1.5	−1.1
Se	**II**	−6.2	−5.7	−4.9	−4.8	−5.3	−2.3	−1.7
Te	**II**	−7.8	−7.5	−6.1	−6.9	−7.6	−3.6	−3.7

[a] B3LYP‐D3BJ/TZVP,cc‐pVTZ. [b] B2PLYP‐D3/TZVP,cc‐pVTZ. [c] B2PLYP‐D3/cc‐pVTZ//B2PLYP‐D3/TZVP,cc‐pVTZ. [d] CCSD/TZVP,cc‐pVTZ//B2PLYP‐D3/TZVP,cc‐pVTZ. [e] CCSD(T)/TZVP,cc‐pVTZ//B2PLYP‐D3/TZVP,cc‐pVTZ. [f] B2PLYP‐D3(water as solvent)/cc‐pVTZ//B2PLYP‐D3/TZVP,cc‐pVTZ. [g] CCSD(water as solvent)/TZVP,cc‐pVTZ//B2PLYP‐D3/TZVP,cc‐pVTZ.

A glance at the lengths of the N⋅⋅⋅H−N bonds in the complexes **1⋅2** reveals that the thiazole complex **1 b⋅2** exhibits the smallest distance (Table 1). However, the differences between the N⋅⋅⋅H−N bonds amount to a maximum of ca. 0.01 Å. The energies for the N⋅⋅⋅H−N bonds were relatively independent of the identity of the chalcogen atom in complexes **1 a**–**1 d**. Depending on the method used, the energies of the hydrogen bonds amount to −8.3 to −10.4 kcal mol^−1^ in the gas phase (Table 2).

A completely different picture emerges when the C=O⋅⋅⋅E chalcogen bonds between the azoles and the amides are considered. A stabilizing chalcogen bond is only found for thiazole, selenazole, and tellurazole, whereby the distance between the chalcogen centers decreases from S via Se to Te. For example, the calculated C=O⋅⋅⋅S distance amounts to 3.25 Å; for the complex **1 d⋅2** a value of 3.0 Å is found for the C=O⋅⋅⋅Te bond (Table 1). Accordingly, the energies of the C=O⋅⋅⋅E interactions show the same trend. If the absolute values obtained by CCSD(T) calculations are considered, the energy of the C=O⋅⋅⋅Te bond (**1 d⋅2**: −7.6 kcal mol^−1^) is almost 3 kcal mol^−1^ higher in energy than the C=O⋅⋅⋅S interaction (**1 b⋅2**: −4.7 kcal mol^−1^). It should be noted that at the same level of theory (CCSD(T)), the C=O⋅⋅⋅Te bond in **1 d⋅2** (type **II**) is less favorable by 1.3 kcal mol^−1^ than the corresponding N⋅⋅⋅H−N bond in the type **I** conformation of **1 d⋅2**. However, these data are obtained in the gas phase. In order to take solvent effects into account (SMD^49^ model), calculations were also performed in water as a solvent. Using this model, the electrostatic components lose their importance in the attractive part of the interaction energy. As a result, the complex formation of **1 d⋅2** via a C=O⋅⋅⋅Te chalcogen bond (type **II**; −3.7 kcal mol^−1^) is energetically more favored than via a N⋅⋅⋅H−N hydrogen bond (type **I**; −2.5 kcal mol^−1^) according to CCSD calculations.

**Figure 1:**
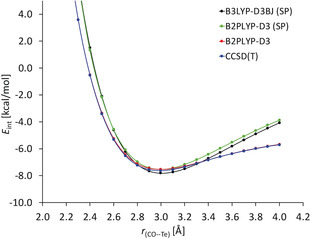
Potential energy curves for the interaction between 1,3‐tellurazole (**1 d**) and acetamide (**2**) via a C=O⋅⋅⋅Te chalcogen bond (type **II**) as derived by B2PLYP‐D3 and CCSD(T).

In the next step we wanted to study how strongly the energy of the C=O⋅⋅⋅Te chalcogen bond depends on the distance between the two chalcogen centers. For this purpose, we carried out different model calculations. In the first case, the distance between the oxygen and the tellurium atom was varied, whereby all other parameters of the optimized structure of the complex **1 d⋅2** were maintained. As methods, B3LYP‐D3BJ and B2PLYP‐D3 were applied. The curves obtained by this procedure are named B3LYP‐D3BJ(SP) and B2PLYP‐D3(SP) in Figure 1. In the second case, the distance between the oxygen and the tellurium atom was fixed at given values and all other parameters were optimized by means of B2PLYP‐D3. Furthermore, single‐point calculations using the CCSD(T) approximation were performed on these structures. The thus obtained curves (named as B2PLYP‐D3 and CCSD(T) in Figure 1) are very similar. It can also be seen that all curves show a flat region from 2.8 to 3.2 Å. This means that starting from an ideal distance between the oxygen and the tellurium atom of 3.0 Å, an increase or decrease of the distance *d*(O⋅⋅⋅Te) by 0.2 Å results in a reduction of the bond strength of at most 5 %.

It is also interesting to compare the C=O⋅⋅⋅Te chalcogen bond with the N⋅⋅⋅Te (type **III**) and Te⋅⋅⋅Te (type **IV**) chalcogen bonds in the dimers of **1 d** (Scheme 1 b). The calculated binding energies (CCSD(T)) are −6.8 kcal mol^−1^ for dimerization via a N⋅⋅⋅Te bond (type **III**) and −5.2 kcal mol^−1^ for the dimerization via a Te⋅⋅⋅Te bond (type **IV**). Thus, their absolute values are slightly lower than the corresponding value for the C=O⋅⋅⋅Te bond (type **II**; −7.6 kcal mol^−1^). The calculated data lead to the conclusion that the chalcogen bond between a tellurazole and a carbonyl group is in the same range as the corresponding N⋅⋅⋅H−N hydrogen bond. Therefore, a tellurazole and a carbonyl group should be usable as recognition units in a supramolecular system.

### Intramolecular Chalcogen Bonds vs. Hydrogen Bonds

**Scheme 2:**
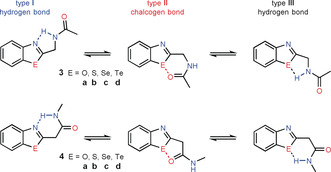
Structure stabilization by N⋅⋅⋅H−N hydrogen bonds (type **I**; blue centers), C=O⋅⋅⋅E chalcogen bonds (type **II**; red centers), and E⋅⋅⋅H−N hydrogen bonds (type **III**) in the benzazoles **3** and **4**.

In a second step we intended to examine whether the C=O⋅⋅⋅E chalcogen bonds can compete with intramolecular formed N⋅⋅⋅H−N and E⋅⋅⋅H−N hydrogen bonds. Therefore, we investigated the two model systems **3** and **4** using quantum chemical model calculations (Scheme 2). The computations were performed analogously to the investigations of the intermolecular interactions (see above). The structure and energy data are summarized in Tables 3 and 4.

**Table 3:** Calculated distances [Å] of the N⋅⋅⋅H−N hydrogen bonds, C=O⋅⋅⋅E chalcogen bonds and E⋅⋅⋅H−N hydrogen bonds in the structures of molecules **3** and **4** calculated using different levels of theory.

	E	Type	*d*(NH⋅⋅⋅N)^[a]^	*d*(NH⋅⋅⋅N)^[b]^	type	*d*(CO⋅⋅⋅E)^[a]^	*d*(CO⋅⋅⋅E)^[b]^	Type	*d*(NH⋅⋅⋅E)^[a]^	*d*(NH⋅⋅⋅E)^[b]^
**3 a**	O	**I**	2.314	2.310				**III**	2.656	2.634
**3 b**	S	**I**	2.250	2.254	**II**	3.313	3.244			
**3 c**	Se	**I**	2.255	2.256	**II**	3.167	3.126			
**3 d**	Te	**I**	2.255	2.254	**II**	3.045	3.009			
										
**4 a**	O	**I**	2.015	2.054				**III**	2.168	2.165
**4 b**	S	**I**	2.084	2.108						
**4 c**	Se	**I**	2.095	2.122	**II**	2.973	3.014			
**4 d**	Te	**I**	2.131	2.183	**II**	2.882	2.894

[a] B3LYP‐D3BJ/TZVP,cc‐pVTZ. [b] B2PLYP‐D3/TZVP,cc‐pVTZ.

**Table 4:** Energies [kcal mol^−1^] of the conformers **II** (for **3 b**‐**d** and **4 c**‐**d**; C=O⋅⋅⋅E chalcogen bonds) and conformers **III** (for **3 a** and **4 a**; E⋅⋅⋅H−N hydrogen bonds) relative to the energy of the conformers **I** (N⋅⋅⋅H−N hydrogen bonds) by means of different computation methods.

	E	Δ*E* ^[a]^	Δ*E* ^[b]^	Δ*E* ^[c]^	Δ*E* ^[d]^	Δ*E* ^[e]^	Δ*E* ^[f]^	Δ*E* ^[g]^
**3 a**	O	1.36	1.82	2.31	1.50	1.20	0.42	−0.32
**3 b**	S	0.22	−0.23	0.04	−0.25	−0.79	−1.37	−1.66
**3 c**	Se	−0.66	−1.07	−0.76	−0.98	−1.59	−1.11	−1.23
**3 d**	Te	−2.27	−3.02	−2.71	−3.41	−4.29	−2.16	−2.91
								
**4 a**	O	3.12	1.97	2.11	1.51	1.58	−0.31	−0.78
**4 c**	Se	1.73	1.60	1.54	1.11	1.26	−0.21	−0.44
**4 d**	Te	−0.56	−0.74	−0.92	−1.33	−1.23	−1.82	−2.14

[a] B3LYP‐D3BJ/TZVP,cc‐pVTZ. [b] B2PLYP‐D3/TZVP,cc‐pVTZ. [c] B2PLYP‐D3/cc‐pVTZ//B2PLYP‐D3/TZVP,cc‐pVTZ. [d] CCSD/TZVP,cc‐pVTZ//B2PLYP‐D3/TZVP,cc‐pVTZ. [e] CCSD(T)/TZVP,cc‐pVTZ//B2PLYP‐D3/TZVP,cc‐pVTZ. [f] B2PLYP‐D3(water as solvent)/cc‐pVTZ//B2PLYP‐D3/TZVP,cc‐pVTZ. [g] CCSD(water as solvent)/TZVP,cc‐pVTZ//B2PLYP‐D3/TZVP,cc‐pVTZ.

The calculations show that the benzoxazoles **3 a** and **4 a** do not exhibit stabilizing C=O⋅⋅⋅O chalcogen interactions and consequently exist as conformers with either N⋅⋅⋅H−N or O⋅⋅⋅H−N hydrogen bonds, whereby the conformers **I** (N⋅⋅⋅H−N bonds) are more stable in the gas phase than the conformers **III** with O⋅⋅⋅H−N hydrogen bonds (Table 4). In water as solvent the conformers **I** and **III** exhibit almost the same energy. For the benzothiazole **3 b** and the benzoselenazole **4 c**, the equilibrium position depends on the used calculation method and the considered medium. Thus, in these two systems it can be expected that the two stabilizing effects (chalcogen bond vs. N⋅⋅⋅H−N hydrogen bond) will be balanced. In the case of the benzoselenazole **3 c** and the benzotellurazoles **3 d** and **4 d**, the conformers **II** that are stabilized by C=O⋅⋅⋅E are favorable compared to the conformers **I** with N⋅⋅⋅H−N hydrogen bonds. The highest difference between the conformers is found for the benzotellurazoles **3 d** and amounts to −4.3 kcal mol^−1^ in the gas phase (CCSD(T)) and −2.9 kcal mol^−1^ in water as solvent (CCSD). Thus, in the benzotellurazole **3 d** and similar systems we expect a clear preference for an intramolecular chalcogen bond over an intramolecular hydrogen bond.

**Scheme 3:**
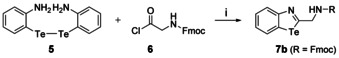
Synthesis of the benzotellurazole **7 b**. Reaction conditions: i) H_3_PO_2_, HCl_conc._, EtOH, THF, Δ, 43 %.

In a next step, we intended to examine whether the C=O⋅⋅⋅Te chalcogen bonds, which are predicted by the above‐mentioned calculations, can be confirmed experimentally. We chose the benzotellurazoles **7** (**7 a**: R=Moc; **7 b**: R=Fmoc) as test systems (Scheme 3 and Table S1). Computation of the model system **7 a** (R=Moc) shows that the energy difference between the conformers in the carbamate **7 a** resembles that found for the amide **3 d** (see Table S2). In both cases the conformer with the C=O⋅⋅⋅Te chalcogen bond is more stable than the structure showing a N⋅⋅⋅H−N hydrogen bond. The model compound **7 b** (R=Fmoc) can be easily synthesized from ditelluride **5** in analogy to a known procedure^50^ and it is very similar to the model system **3 d**, which shows the highest preference for an intramolecular C=O⋅⋅⋅Te chalcogen bond over an intramolecular N⋅⋅⋅H−N hydrogen bond.

**Figure 2:**
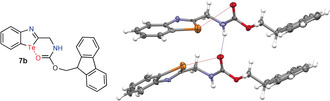
Formula (left) and solid‐state structure (right) of benzotellurazole **7 b**. Four independent molecules are found in the asymmetric unit; only two of them are displayed and the minor components of the disorder omitted for clarity. Displacement ellipsoids are drawn at the 50 % probability level, and hydrogen atoms as spheres of arbitrary radii.

To study the structure of **7 b** in the solid state, a single crystal was measured using X‐ray structure analysis. There are four molecules in the asymmetric unit (Figure 2). The benzotellurazole rings of the molecules are disordered over two alternate positions. The ratio of occupancy for the components is approximately 1:2. As a consequence of disorder, the values for the distances must be interpreted with caution. In all four molecules, the carbonyl groups are oriented in such a way that they can form a chalcogen bond to the tellurium atoms. The *d*(C=O⋅⋅⋅Te) distances are 3.262(10), 3.309(9), 3.251(11), and 3.428(11) Å and clearly demonstrate the formation of C=O⋅⋅⋅Te chalcogen bonds. In addition to these intramolecular C=O⋅⋅⋅Te chalcogen bonds, there are also chalcogen–chalcogen interactions between tellurium atoms, whereby the Te⋅⋅⋅Te distances amount to 3.410(5), 3.592(5), 3.611(5), 3.622(6) and 3.574(7) Å. They are distinctly smaller than the sum of the van der Waals radii of two tellurium atoms (4.12 Å).^51^, ^52^ One might speculate that the competitive formation of C=O⋅⋅⋅Te and Te⋅⋅⋅Te interactions is the cause for the disorder. Furthermore, there also exist hydrogen bonds which are formed intermolecularly between the carbamate units. This example shows, that in the case of favorable geometric conditions the formation of a carbonyl–tellurium bond can be more favored than hydrogen bonding.

### Intermolecular Chalcogen Bond as Structure‐Forming Unit

**Scheme 4:**
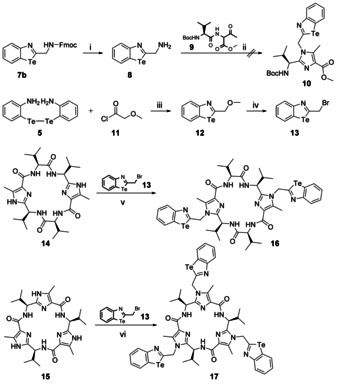
Synthesis of the cyclic peptides **16** and **17** showing two and three benzotellurazole recognition units, respectively. Reaction conditions: i) piperidine, DMF, 56 %; ii) CF_3_COOH, xylenes, Δ; iii) H_3_PO_2_, HCl_conc._, EtOH, THF, Δ, 46 %; iv) BBr_3_, DCM, 74 %; v) Cs_2_CO_3_, CH_3_CN, 28 %; vi) Cs_2_CO_3_, CH_3_CN, 36 %.

Based on these promising results, we attempted to use the carbonyl⋅⋅⋅tellurazole chalcogen bond as a recognition unit for intermolecular interactions. Therefore, we intended to synthesize the cyclic peptides **16** and **17** (Scheme 4) having two different spheres: The interior of the macrocycles is dominated by the nitrogen atoms of the imidazoles having free electron pairs and the hydrogen atoms of the amide bonds. These functional groups form a polar self‐complementary pattern of hydrogen donors and acceptors.^53^, ^54^ The outer sphere of the macrocycles consists of the carbonyl groups of the amides and the benzotellurazole units, which are bonded to the nitrogen atoms via a methylene group. These functional groups protruding outward should be able to form intra‐ or intermolecular interactions.

For the synthesis of cyclic peptides **16** and **17**, we intended to follow a well‐known pattern. Accordingly, we wanted to synthesize the imidazole building block **10** starting from amidoketone **9** and the corresponding amine **8** (Scheme 4). The latter can readily be obtained from **7 b** by deprotection. The imidazole **10** could subsequently be oligomerized to the desired macrocycles **16** and **17**. Although we tested different reaction conditions, we were not able to isolate the desired imidazole building block **10**; it was impossible to determine whether the imidazole **10** was unstable or not formed under the reaction conditions. We assume that the acidic conditions and the high temperatures required for cyclization to the imidazole ring cause decomposition of the benzotellurazole unit. To avoid these conditions, we looked for an alternative route. Therefore, we intended to introduce the benzotellurazole entities in the final step of the synthesis to the cyclic peptides **16** and **17** via alkylation of the cyclic peptides **14** and **15** with bromide **13** (Scheme 4). The latter can be prepared in only two steps and in good yield starting from the ditelluride **5** and the acid chloride **11** (Scheme 4). The cyclic peptides **14** and **15** required for this approach have already been used in the design of molecular motors^55^, ^56^, ^57^ and supramolecular systems^58^, ^59^ and can be synthesized according to known procedures.^53^, ^60^, ^61^ The alkylation reaction of the cyclic peptides **14** and **15** with bromide **13** gave the target macrocycles **16** and **17** in rather good yields.

A study of the solution structure of the macrocycles **16** and **17** reveals that the structure of the peptidic skeleton resemble that of similar peptides lacking benzotellurazole units in solution and the solid state (see the Supporting Information). The latter form a polar self‐complementary pattern of hydrogen donors and acceptors.^53^, ^54^, ^62^

**Figure 3:**
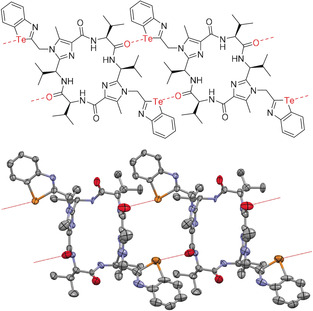
Schematic representation of the interaction (left) and molecular structures (right) of two molecules **16** in the solid state. The chalcogen bonds R_2_C=O⋅⋅⋅Te between the molecules are indicated by red dashed lines. The distances *d*(CO⋅⋅⋅Te) amount to 3.173(15) Å and 3.241(19) Å. Displacement ellipsoids are drawn at the 50 % probability level. All hydrogen atoms and solvent molecules are omitted for the sake of clarity.

To investigate the structures of the cyclic peptides **16** and **17** in the solid state, we tried to crystallize them from different solvent mixtures. We obtained dark yellow to brownish crystals of peptides **16** and **17**, which were suitable for X‐ray structure analysis. In the case of **16** the crystals were obtained from a mixture of dichloromethane (DCM) and methanol; for the cyclic peptide **17** we used a mixture of dichloromethane and diethyl ether. The solid‐state structure of cyclic peptide **16** is depicted in Figure 3 and Figure S6. It can be seen that the molecules form wires in the solid state. The single wires are twisted relative to each other in such a way that the gap formed by one wire is filled by the bulge of another wire, allowing a maximum of stabilizing interactions. Between the chains of the cyclic peptide molecules a large number of water molecules are found in the solid‐state structure (Figure S1). They are especially located near the inside of the cyclopeptide ring forming hydrogen bonds to the carbonyl units and NH protons of the peptidic ring. As expected, the nitrogen atoms of the imidazoles that have free electron pairs and the hydrogen atoms of the amide bonds point to the interior of the macrocycle forming a rim of hydrogen bonds. These hydrogen bonds are geometrically almost perpendicular to the chalcogen bonds that share a common amide group. The dihedral angles θ(Te‐O‐H‐N) amount to 104° and 108°. An analogous phenomenon has already been found in halogen bonding studies involving peptides:^63^, ^64^ The halogen bonds are formed perpendicular to the hydrogen bonding networks so that the pattern of the hydrogen bonds remains unaltered.

The wires themselves are held together by carbonyl⋅⋅⋅tellurazole chalcogen bonds, whereby each cycle provides four of these bonds: two each with its two neighbors. The schematic representation of these interactions and the molecular structures of two molecules **16** in the solid state are shown in Figure 3. The distances between the C=O⋅⋅⋅Te bonds are 3.173(15) Å and 3.241(19) Å and thus significantly smaller than the sum of the van der Waals radii (3.58 Å).^51^, ^52^ Based on the above calculation for the model system **1 d⋅2** (Figure 1), it can be assumed that a molecule **16** is captivated in the wire with an energy of approximately 28 kcal mol^−1^ by these four C=O⋅⋅⋅Te bonds. In other words, the C=O⋅⋅⋅Te bonds are crucial for the wire formation.

**Figure 4:**
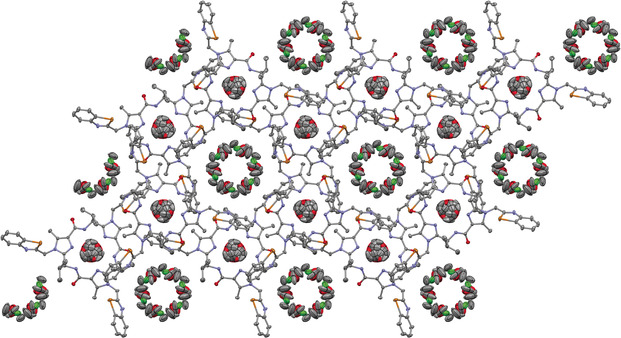
Top view of the honeycombs in the solid‐state structure of **17**. Displacement ellipsoids are drawn at the 50 % probability level. All hydrogen atoms and solvent molecules are omitted for the sake of clarity.

A totally different picture emerges for the cycle **17**. Here, a three‐dimensional framework is found. Considering the structure of **17** in the solid state along the 3‐axis, a honeycomb‐like structure can be recognized (Figure 4). However, in contrast to the classic honeycomb, the molecules are arranged to form two types of channels: a large one formed by the 6_3_‐axis and a small one formed by the 3‐axis. Both are filled with disordered solvent molecules. The solvent molecules found in the large channel are methylene chloride and diethyl ether. In the small channel only diethyl ether is present. Due to the disorder, an exact determination of the orientation of the solvent molecules is not possible. The large channel has an approximate diameter of 9 Å and is built‐up by the benzotellurazole units and the outward‐pointing parts of the peptidic system. The second channel is significantly smaller and is formed by the benzotellurazole entities and the parts of the peptide system pointing into the interior. A closer look reveals that the diameter of the small channel does not have same size throughout the channel, but it fluctuates between 4 and 6 Å. The narrowing is caused by the intrusion of the nitrogen atoms of the imidazoles and the hydrogen atoms of amides into the interior of the macrocycle, forming a polar self‐complementary pattern of hydrogen donors and acceptors. Consequently, the solvent molecules in the small channels are presumably not freely movable, but they are restricted to a single chamber. On the other hand, the approximately doubled diameter of the large channel suggests that free movement of the solvent molecules along the channel may be possible.

**Figure 5:**
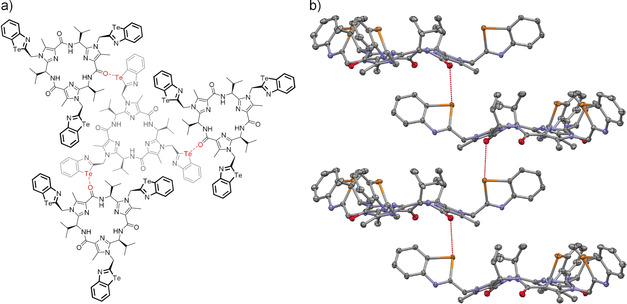
a) Schematic representation of the chalcogen bonds (R_2_C=O⋅⋅⋅Te) between four molecules **17** in the solid state (view parallel to the *c*‐axis). The three outer molecules (displayed in black) are located in a higher layer than the central molecule (displayed in gray). b) Interactions between four layers of **17** in the solid state (view perpendicular to the *c*‐axis). The indicated distances *d*(CO⋅⋅⋅Te) amount to 3.178(3) Å. Displacement ellipsoids are drawn at the 50 % probability level. All hydrogen atoms and solvent molecules are omitted for the sake of clarity.

An analysis of the interactions between the molecules **17** in solid‐state structure indicates that the honeycombs are formed by carbonyl⋅⋅⋅tellurazole chalcogen bonds which are geometrically almost perpendicular to the hydrogen bonds within the macrocycle. The dihedral angles θ(Te‐O‐H‐N) amount to −80°. Due to the symmetry in the crystal, each molecule **17** forms a total of six C=O⋅⋅⋅Te bonds, which have an identical distance of 3.178(3) Å. This value deviates only slightly from the ideal distance of 3.0 Å for the interaction between a tellurazole and the carbonyl group of acetamide (see the previous section). According to the calculations, a bond extension of 0.18 Å leads to a reduction of the attractive interaction energy from −7.6 kcal mol^−1^ to −7.4 kcal mol^−1^. Thus, the deviation from an ideal interaction is very small. The C=O⋅⋅⋅Te bonds in the honeycomb‐like structure of **17** is formed between molecules belonging to different planes (see Figure 5). For instance, one molecule interacts with three molecules of the level above via the tellurium atoms of the benzotellurazole and with three molecules of the level below via three carbonyl groups. This pattern generates the honeycomb‐like system of the solid structure which has already been described above.

A modification of the size of the channels should be possible via the size of the macrocycle and the type of connection between the macrocycle and benzotellurazole units: an enlargement of the macrocycle should lead to an enlargement of both channels. Changing the linkage between the macrocycle and benzotellurazoles should only affect the size of the large channel.

## Conclusion

We were able to introduce the carbonyl⋅⋅⋅tellurazole chalcogen bond as recognition unit. According to quantum chemical calculations, the energy of such a C=O⋅⋅⋅Te bond amounts to about −7.5 kcal mol^−1^ on the CCSD(T) level of theory. The distance between the oxygen and the tellurium atom in this chalcogen bond was calculated to be about 3.0 Å, whereby a deviation of 0.2 Å from the ideal bond distance results in a reduction of the bond strength of only 5 %. Thus, this bond type has a very flat region from 2.8 to 3.2 Å. Using this bonding motif, we were able to design complex supramolecular networks in the solid phase starting from tellurazole‐substituted cyclic peptides. In one case, the tellurazole‐substituted macrocycle exhibits two benzotellurazole units and forms chains connected via C=O⋅⋅⋅Te bonds. In the second case, a honeycomb‐like supramolecular organic framework (SOF) formed by carbonyl⋅⋅⋅tellurazole chalcogen bonds was observed. This honeycomb‐like structure shows two different types of channels filled with different types of solvent mixtures. In the future, this concept should make it possible to design SOFs based on carbonyltellurazole chalcogen bonds with both permanent and size‐controllable channels.

## Supporting Information

Figures, synthesis of the new compounds, computational details, cartesian coordinates, and absolute energies for all calculated compounds, crystal structure data as well as ^1^H NMR and ^13^C NMR spectra of new compounds. Deposition numbers 1995939 (**7 b**), 1995940 (**16**) and 1995941 (**17**) contain the supplementary crystallographic data for this paper. These data are provided free of charge by the joint Cambridge Crystallographic Data Centre and Fachinformationszentrum Karlsruhe Access Structures service.

## Conflict of interest

The authors declare no conflict of interest.

## Supporting information

As a service to our authors and readers, this journal provides supporting information supplied by the authors. Such materials are peer reviewed and may be re‐organized for online delivery, but are not copy‐edited or typeset. Technical support issues arising from supporting information (other than missing files) should be addressed to the authors.

Supplementary
