# Supplementary material for: The Carbonyl⋅⋅⋅Tellurazole Chalcogen Bond as a Molecular Recognition Unit: From Model Studies to Supramolecular Organic Frameworks
Source: Angew Chem Int Ed Engl. 2020 Jul 29;59(39):17154–61. doi: 10.1002/anie.202005374 (PMC7540342; doi:10.1002/anie.202005374)
Supplement: Supplementary file 1 — Supplementary [file ANIE-59-17154-s001.pdf]

Angewandte Chemie
Eine Zeitschrift der Gesellschaft Deutscher Chemiker

Supporting Information

The Carbonyl···Tellurazole Chalcogen Bond as a Molecular Recognition Unit: From Model Studies to Supramolecular Organic Frameworks

Saber Mehrparvar, Christoph Wölper, Rolf Gleiter, and Gebhard Haberhauer**

anie_202005374_sm_miscellaneous_information.pdf

Supporting Information

1. **Figures and Tables** S2
2. **Investigation of the Structures of 16 and 17 in Solution** S6
3. **Synthesis of New Compounds** S8
4. **Computational Details** S14
5. **Cartesian Coordinates and Absolute Energies for All Calculated Compounds** S15
6. **Crystal Structure Data** S39
7. **^{1}H NMR and ^{13}C NMR Spectra of New Compounds** S45
8. **Supporting Information References** S52

1. Figures and Tables

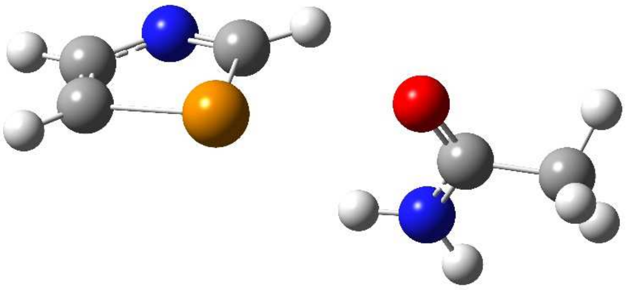

Figure S1. Molecular structure of **1d•2** (type **II**) calculated by means of B2PLYP-D3/TZVP,cc-pVTZ.

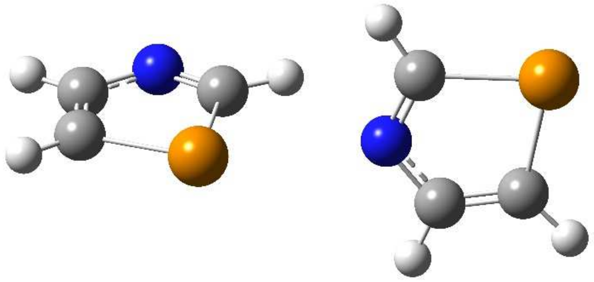

Figure S2. Molecular structure of **1d•1d** (type **III**) calculated by means of B2PLYP-D3/TZVP,cc-pVTZ.

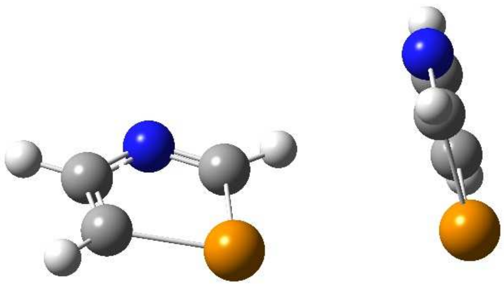

Figure S3. Molecular structure of **1d•1d** (type **IV**) calculated by means of B2PLYP-D3/TZVP,cc-pVTZ.

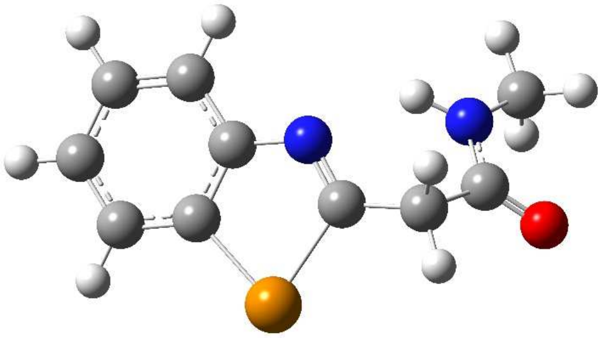

Figure S4. Molecular structure of **3d** (type **I**) calculated by means of B2PLYP-D3/TZVP,cc-pVTZ.

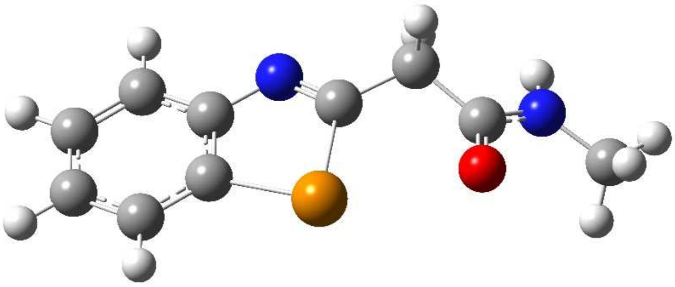

Figure S5. Molecular structure of **3d** (type **II**) calculated by means of B2PLYP-D3/TZVP,cc-pVTZ.

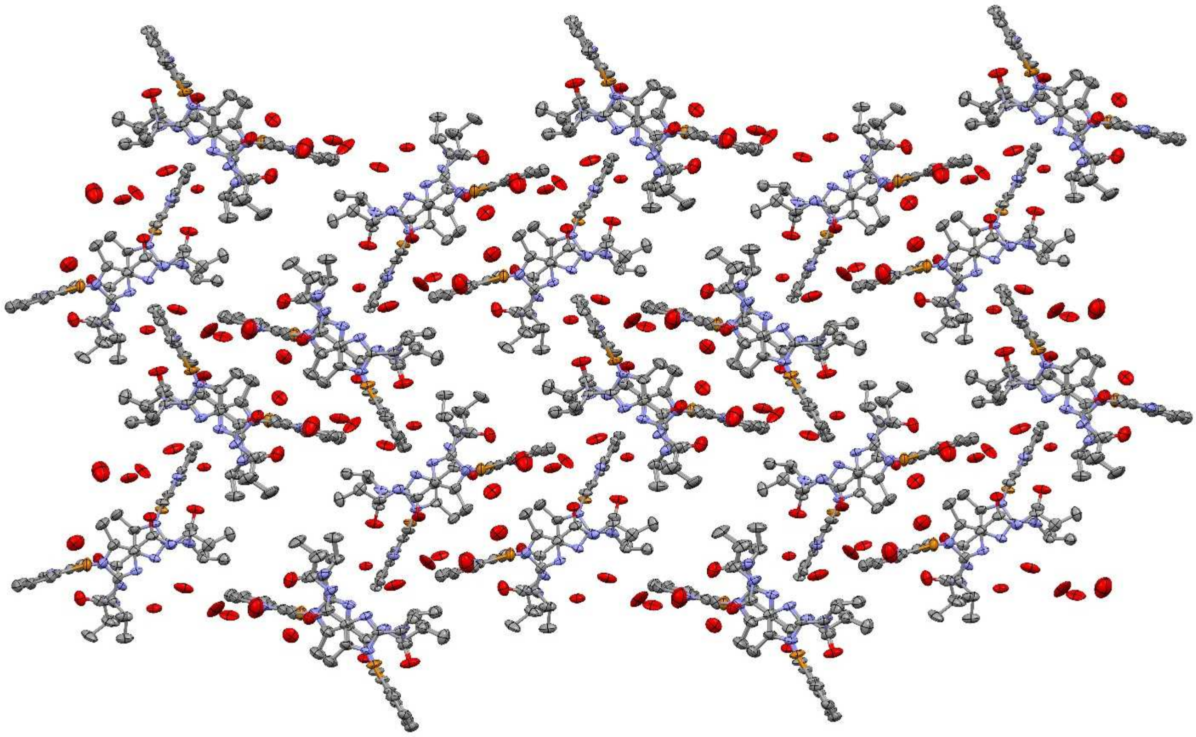

Figure S6. Top view of the chains in the solid state of **16**. All hydrogen atoms are omitted for the sake of clarity. Displacement ellipsoids are drawn at the 50% probability level. All hydrogen atoms and solvent molecules are omitted for the sake of clarity.

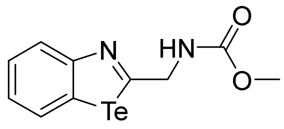

7a

Table S1. Calculated distances [Å] of the N···H-N hydrogen bonds and C=O···E chalcogen bonds in the structures of molecule **7a** calculated using different level of theory.

type	*d*(NH···N)[a]	*d*(NH···N)[b]	type	*d*(CO···E)[a]	*d*(CO···E)[b]
**I**	2.255	2.254	**II**	3.045	3.009

[a] B3LYP-D3BJ/TZVP,cc-pVTZ.

[b] B2PLYP-D3/TZVP,cc-pVTZ.

Table S2. Energy [kcal/mol] of the conformer **II** (C=O···E chalcogen bonds) relative to the energy of the conformer **I** (N···H-N hydrogen bonds) of **7a** by means of different computation methods.

ΔE^a	ΔE^b	ΔE^c	ΔE^d	ΔE^e	ΔE^f	ΔE^g
-2.49	-3.16	-2.90	-3.38	-4.07	-2.51	-2.99

[a] B3LYP-D3BJ/TZVP,cc-pVTZ.

[b] B2PLYP-D3/TZVP,cc-pVTZ.

[c] B2PLYP-D3/cc-pVTZ//B2PLYP-D3/TZVP,cc-pVTZ.

[d] CCSD/TZVP,cc-pVTZ//B2PLYP-D3/TZVP,cc-pVTZ.

[e] CCSD(T)/TZVP,cc-pVTZ//B2PLYP-D3/TZVP,cc-pVTZ.

[f] B2PLYP-D3(water as solvent)/cc-pVTZ//B2PLYP-D3/TZVP,cc-pVTZ.

[g] CCSD(water as solvent)/TZVP,cc-pVTZ//B2PLYP-D3/TZVP,cc-pVTZ.

2. Investigation of the Structures of 16 and 17 in Solution

The NMR spectra (^{1}H, ^{13}C) for the cyclic peptides **16** and **17** indicate that in solution, they are C_2- and C_3-symmetric, respectively. The doublets of the amide NH resonances in **17** are shifted about δ = 0.5 to 1.5 ppm further downfield than those in **16**, suggesting that the interaction between the lone pairs of the imidazole nitrogen and the hydrogen of the secondary amides are probably stronger in **17** than in **16** (Table S3). The vicinal coupling constant ($^{3}J_{NHCH}$) for the amide NH resonance in **17** is 9.0 to 9.3 Hz and corresponds to NHαCH dihedral angles of 153° < $|\theta|$ < 156° in solution (Table S3).[1] The dihedral angles found for **17** in solid state amounts to 156°. Thus, the structure of the peptidic skeleton in solid state corresponds to that in solution.

In the case of the cyclic peptide **16**, the vicinal coupling constants are measured to be 7.8 to 10.7 Hz (Table S3). Accordingly, dihedral angles between 144° and 174° are expected. In solid state the NHαCH dihedral angles amount to -145°, -151°, -157° and -163°, which agree with the values determined by ^{1}H NMR spectroscopy. The low dependence of the vicinal coupling constants on the solvent shows that the geometry of the macrocycles **16** and **17** is essential the same in all solvents. Overall, the NMR data of the cycles **16** and **17** resemble that of similar peptides lacking benzotellurazole units.[2-3] This allows the conclusion that the benzotellurazole units have no impact on the solution structure of the cyclopeptides.

Table S3. Values for the NH amide resonances [ppm] and vicinal coupling $^{3}J_{HNCH}$ [Hz] of the cyclic peptides **16** and **17** in different solvents.

Solvent	δ [ppm]			$^{3}J_{HNCH}$ [Hz]		
	**16**	**16**	**17**	**16**	**16**	**17**
$CDCl_3$	6.814	7.156	8.528	8.23	10.68	9.12
$CDCl_3$ + 1 eq. TBACl	6.836	---[a]	8.511	7.75	---[a]	9.05
$CDCl_3$ + 2 eq. TBACl	6.861	---[a]	8.509	---[a]	---[a]	8.78
CD_3CN	6.996	7.884	8.314	7.87	10.38	8.96
CD_3COCD_3	7.198	7.911	8.488	8.70	10.42	8.99
DMSO	7.707	7.972	8.366	8.03	10.62	9.04
DMSO-D_6 + 20 eq. KCl	7.783	8.076	8.361	7.93	10.67	9.01
DMSO-D_6 + 50 eq. KCl	7.815	8.119	8.360	8.04	10.62	9.01
CD_3OD	---[b]	---[b]	8.577	---[b]	---[b]	9.27

[a] not determinable. [b] no signals due to a rapid H/D exchange.

The addition of chloride anions results in no significant change of the ^1H NMR spectra of **16** and **17** and accordingly to no significant change of the structure in solution. NMR titration of **16** and **17** with TBACl (tetrabutylammonium chloride) in $CDCl_3$ and KCl in DMSO-D_6 leads to following binding constants:

K_a (**16** + TBACl in $CDCl_3$) = 45.4 ± 3.5 M^{-1}

K_a (**17** + TBACl in $CDCl_3$) = 154.4 ± 4.3 M^{-1}

K_a (**16** + KCl in DMSO-D_6) = 2.98 ± 1.39 M^{-1}

K_a (**17** + KCl in DMSO-D_6) = 58.2 ± 3.4 M^{-1}

The fitting was performed using Bindfit (http://app.supramolecular.org/bindfit/) assuming a binding stoichiometry 1:1. These binding constants resemble those of similar peptides lacking benzotellurazole units, which suggest that the hydrogen atoms of the amide groups are responsible for the binding.[4]

3. Synthesis of New Compounds

General remarks. All chemicals were reagent grade and used without further purification. Reactions were monitored by TLC analysis with silica gel 60 F254 thin-layer plates. Flash chromatography was carried out on silica 60 (40–63 µm, 230–400 mesh). ^{1}H and ^{13}C NMR spectra were measured with Bruker Avance DRX 500 and Avance HD 600 spectrometers. All chemical shifts (*δ*) are given in ppm. The spectra were referenced to the peak for the protium impurity in the deuterated solvents indicated in brackets in the analytical data. Signal multiplicity for ^{1}H NMR was determined as s (singlet), d (doublet), t (triplet), sext (sextet), sept (septet), m (multiplet), dd (doublet of doublets) and td (triplet of doublets). ^{13}C NMR spectra were measured with ^{1}H decoupling and the ^{13}C assignment was achieved via DEPT 135, HSQC, HMBC, and COSY spectra. ^{13}C signal multiplicity was determined as p (primary), s (secondary), t (tertiary), q (quaternary). HR-MS spectra were recorded with a Bruker BioTOF III Instrument with ESI as ionization source. UV/Vis absorption spectra were obtained with a Jasco V-550 spectrophotometer. CD absorption spectra were recorded with a Jasco J-815 spectrophotometer. IR absorption spectra were recorded with a Shimadzu IR Tracer-100 FTIR spectrophotometer. Ditelluride **5**[5], amide ketone **9**[6], and macrocycle **15**[7] were synthesized according to known procedures. Macrocycle **14**[8] was purchased from Squarix GmbH.

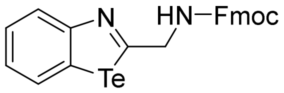

7b

Synthesis of benzotellurazole 7b. Ditelluride **5** (3.29 g, 7.50 mmol) was dissolved in THF (50 mL) at room temperature. The mixture was stirred for 5 min until all ditelluride had gone into solution. Then acid chloride **6** (4.79 g, 15.2 mmol) was added to this solution and stirring continued for 5 min. Afterwards, 1.5 mL of 50% aq. hypophosphorous acid was added. The mixture was stirred for 5 min and this step was followed by an addition of 25 mL of 95% ethanol and 14 mL of 36% hydrochloric acid. This mixture was subsequently allowed to heat to reflux for 3 hours. After cooling to room temperature, the mixture was chilled in an ice-bath for 30 min. Ammonia solution (5% aq.) was added to the cooled mixture to basify to pH = 8 and the solution was repeatedly extracted with DCM. The organic layers were combined, dried over $MgSO_4$ and concentrated in vacuo. The residue was purified by flash column chromatography with silicagel

(n-pentane/EtOAc 5:1 → 3:1) and **7b** was obtained as a yellow solid (3.15 g, 6.55 mmol, 43%). ^{1}H NMR (600 MHz, $CDCl_3$): δ = 8.08 (d, $^{3}J_{H,H}$ = 7.8 Hz, 1 H; $C_{ar}H$), 7.89-7.87 (m, 1 H, $C_{ar}H$), 7.78 (d, $^{3}J_{H,H}$ = 7.8 Hz, 2 H; $C_{ar}H$, $C_{ar}H$), 7.62 (d, $^{3}J_{H,H}$ = 7.2 Hz, 2 H; $C_{ar}H$, $C_{ar}H$), 7.47-7.44 (m, 1 H, $C_{ar}H$), 7.42-7.40 (m, 1 H, $C_{ar}H$), 7.33-7.31 (m, 1 H, $C_{ar}H$), 7.18-7.16 (m, 1 H, $C_{ar}H$), 5.79 (s, H; NH), 4.61 (d, $^{3}J_{H,H}$ = 6.0 Hz, 2 H; OCH_2), 4.51 (d, $^{3}J_{H,H}$ = 7.2 Hz, 2 H; $CH_2C_{tellurazole}$), 4.26 ppm (t, $^{3}J_{H,H}$ = 6.0 Hz, 1 H; C*H*CH_2). ^{13}C NMR (151 MHz, $CDCl_3$): δ = 175.8 (q; $C_{tellurazole}$), 160.5 (q; CO), 156.7 (q; C_{ar}), 143.8 (q; C_{ar}), 141.4 (q; C_{ar}), 134.6 (t; C_{ar}), 131.8 (q; C_{ar}), 127.9 (q; C_{ar}), 126.3 (t; C_{ar}), 125.1 (t; C_{ar}), 120.2 (t; C_{ar}), 67.3 (s; CH_2O), 51.5 (t; *C*HCH_2), 47.3 ppm (t; *C*$H_2C_{tellurazole}$). ^{125}Te NMR (600 MHz, $CDCl_3$): δ = 900.9 ppm. IR (ATR): ṽ = 3344, 2824, 2816, 1581, 1529, 1421, 1149, 1093, 756 cm^{-1}. UV/Vis (CH_3CN): λ_{max} (log ε) = 261 (4.60), 299 nm (4.02). HRMS (ESI-TOF) m/z: $[C_{23}H_{18}N_2O_2Te+H]^+$ calculated: 485.0503; found: 485.0514.

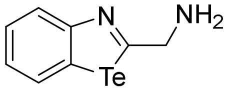

8

Synthesis of benzotellurazole 8. Piperidine (2.0 ml) was added to a solution of tellurazole **7** (500 mg, 1.03 mmol) in DMF (8.0 mL) and the reaction was stirred for 2 h at room temperature. The reaction was concentrated and purified by flash column chromatography eluting with CH_2Cl_2/MeOH (100:0 → 90:10) containing 1% Et_3N to give 150 mg (0.58 mmol, 56%) of benzotellurazole **8** as a yellow oil which was used without further purification. ^{1}H NMR (600 MHz, $CDCl_3$): δ = 8.01 (d, $^{3}J_{H,H}$ = 9.0 Hz, 1 H; $C_{ar}H$), 7.93 (d, $^{3}J_{H,H}$ = 9.0 Hz, 1 H; $C_{ar}H$), 7.45-7.42 (m, 1 H; $C_{ar}H$), 7.15-7.12 (m, 1 H; $C_{ar}H$), 3.99 ppm (s, 2 H; NCH_2). ^{13}C NMR (151 MHz, $CDCl_3$): δ = 162.8 (q; $C_{tellurazole}$), 133.1 (t; C_{ar}), 131.9 (q; C_{ar}), 126.6 (t; C_{ar}), 126.0 (t; C_{ar}), 124.2 (t; C_{ar}), 50.2 ppm (t; NCH_2). ^{125}Te NMR (600 MHz, $CDCl_3$): δ = 874.6 ppm. IR (ATR): ṽ = 3374, 2924, 2850, 1610, 1460, 1155, 734 cm^{-1}. UV/Vis (MeOH): λ_{max} (log ε) = 219 (4.68), 250 nm (3.86). HRMS (ESI-TOF) m/z: $[C_8H_8N_2Te+H]^+$ calculated: 262.9822; found: 262.9833.

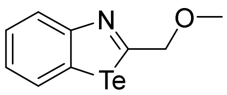

12

Synthesis of benzotellurazole 12. Ditelluride **5** (2.91 g, 6.63 mmol) was dissolved in THF (30 mL) at room temperature. The mixture was stirred for 5 min until all ditelluride had gone into

solution. Then acid chloride **11** (1.54 g, 14.21 mmol) was added to this solution and stirring continued for 5 min. Afterwards, 1.2 mL of 50% aq. hypophosphorous acid was added. The mixture was stirred for 5 min and this step was followed by an addition of 20 mL of 99.8% ethanol and 12 mL of 36% hydrochloric acid. This mixture was subsequently allowed to heat to reflux for 3 hours. After cooling to room temperature, the mixture was chilled in an ice-bath for 30 min. Ammonia solution (5% aq.) was added to the cooled mixture to basify to pH = 8 and the solution was repeatedly extracted with DCM. The organic layers were combined, dried over $MgSO_4$ and concentrated in vacuo. The residue was purified by flash column chromatography with silicagel (*n*-pentane/EtOAc 6:1) and tellurazole **12** was obtained as a yellow solid (1.80 g, 6.55 mmol, 46%). ^{1}H NMR (600 MHz, $CDCl_3$): δ = 8.09-8.07 (m, 1 H; $C_{ar}H$), 7.94-7.93 (m, 1 H; $C_{ar}H$), 7.47-7.45 (m, 1 H; $C_{ar}H$), 7.18-7.15 (m, 1 H; $C_{ar}H$), 4.60 (s, 2 H; CH_2OCH_3), 3.59 ppm (s, 3 H; OCH_3). ^{13}C NMR (151 MHz, $CDCl_3$): δ = 180.3 (q; $C_{tellurazole}$), 160.9 (q; C_{ar}), 133.0 (t; C_{ar}), 131.9 (q; C_{ar}), 126.8 (t; C_{ar}), 124.7 (t; C_{ar}), 78.0 (s; CH_2OCH_3), 59.7 ppm (p; OCH_3). ^{125}Te NMR (600 MHz, $CDCl_3$): δ = 902.4 ppm. IR (ATR): ṽ = 3052, 1525, 1421, 1089 cm^{-1}. UV/Vis (CH_3CN): λ_{max} (log ε) = 240 (4.21), 315 nm (3.47). HRMS (ESI-TOF) *m*/*z*: $[C_9H_9NOTe+H]^+$ calculated: 277.9819; found: 277.8544.

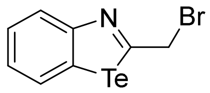

13

Synthesis of benzotellurazole 13. A solution of BBr_3 in CH_2Cl_2 (1 M, 3.82 mmol) was added to a solution of **12** (700 mg, 2.50 mmol) in CH_2Cl_2 (20 mL) at 0 °C. The ice bath was removed and stirring was continued until TLC showed consumption of starting material (3 h). Then the solution was diluted with saturated sodium bicarbonate solution and the aqueous layer was repeatedly washed with CH_2Cl_2. The organic layers were collected, dried over $MgSO_4$, and concentrated under reduced pressure to give **13**, which was used in the next step without further purification due to its instability (600 mg, 1.86 mmol, 74 %). ^{1}H NMR (600 MHz, $CDCl_3$): δ = 8.10-8.09 (m, 1 H; $C_{ar}H$), 7.90-7.88 (m, 1 H; $C_{ar}H$), 7.49-7.46 (m, 1 H; $C_{ar}H$), 7.21-7.19 (m, 1 H; $C_{ar}H$), 4.73 ppm (s, 2 H; CH_2Br). ^{13}C NMR (151 MHz, $CDCl_3$): δ = 174.3 (q; $C_{tellurazole}$), 160.5 (q; C_{ar}), 136.5 (t; C_{ar}), 131.8 (q; C_{ar}), 127.2 (t; C_{ar}), 126.9 (t; C_{ar}), 126.9 (t; C_{ar}), 38.6 ppm (s; CH_2Br). ^{125}Te NMR (600 MHz, $CDCl_3$): δ = 918.3 ppm. IR (ATR): ṽ = 2941, 1682, 1400, 1066,

744 cm^{-1}. UV/Vis (MeOH): λ_{max} (log ε) = 239 (4.41), 318 nm (3.69). HRMS (ESI-TOF) *m*/*z*: $[C_8H_6{}^{79}BrNTe+H]^+$ calculated: 325.8819; found: 325.8806.

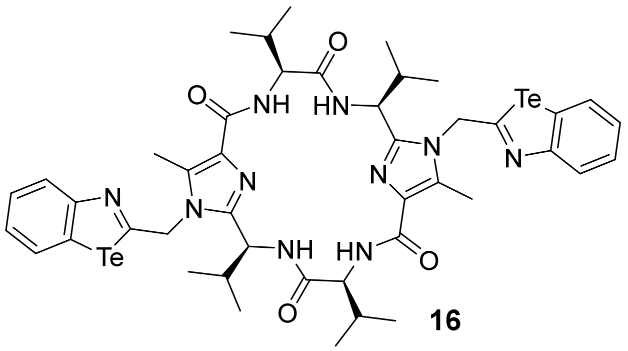

16

Synthesis of cyclic peptide 16. Caesium carbonate (700 mg, 2.15 mmol) was added under argon to a solution of macrocycle **14** (110 mg, 0.2 mmol) in acetonitrile (90 mL). The mixture was stirred for 10 min, then benzotellurazole **13** (650 mg, 2.00 mmol) was added to this solution and stirring was continued at room temperature for 24 h. Subsequently, the solvent was evaporated to dryness, the residue was dissolved in DCM and washed with water. The aqueous layer was saturated with NaCl and then repeatedly extracted with DCM. The organic layers were combined, dried over $MgSO_4$ and concentrated in vacuo. The residue was purified by flash column chromatography with silica gel (DCM/MeOH 99:1 → 99:3) to yield compound **16** (70 mg, 0.05 mmol, 28%) as a yellow solid. ^{1}H NMR (600 MHz, $CDCl_3$): δ = 8.06 (d, $^3J_{H,H}$ = 8.4 Hz, 2 H; NH, NH), 7.87 (d, $^3J_{H,H}$ = 7.2 Hz, 2 H; NH, NH), 7.46-7.43 (m, 2 H; C_{ar}H, C_{ar}H), 7.18-7.15 (d, 4 H; C_{ar}H, C_{ar}H, C_{ar}H, C_{ar}H), 6.79 (d, $^3J_{H,H}$ = 8.4 Hz, 2 H; C_{ar}H, C_{ar}H), 5.45 (d, $^2J_{H,H}$ = 18.0 Hz, 2 H; $CH_2C_{tellurazole}$), 5.25 (d, $^2J_{H,H}$ = 17.4 Hz, 2 H; $CH_2C_{tellurazole}$), 4.92-4.89 (m, 2 H; NHC*H*), 4.53-4.50 (m, 2 H; NHC*H*), 2.54 (s, 6 H; $C_{imi}CH_3$), 2.38-2.32 (m, 1 H; C*H*($CH_3)_2$), 2.32-2.27 (m, 1 H; C*H*($CH_3)_2$), 1.08 (d, $^3J_{H,H}$ = 6.6 Hz, 6 H; CH(C$H_3)_2$), 1.07-1.05 (m, 12 H; CH(C$H_3)_2$),0.89 ppm (d, $^3J_{H,H}$ = 6.6 Hz, 6 H; CH(C$H_3)_2$). ^{13}C NMR (151 MHz, $CDCl_3$): δ = 171.1 (q; $C_{tellurazole}$), 170.5 (q; CO), 162.8 (q; CO), 160.4 (q; C_{ar}), 147.5 (q; C_{ar}), 135.0 (q; C_{ar}), 133.1 (q; C_{ar}), 131.8 (q; C_{ar}), 130.2 (q; C_{ar}), 127.0 (t; C_{ar}), 126.7 (t; C_{ar}), 126.6 (t; C_{ar}), 125.2 (q; C_{ar}), 58.9 (s; $CH_2C_{tellurazole}$), 53.3 (t; CHNH), 51.0 (t; CHNH), 33.4 (t; *C*H($CH_3)_2$), 30.5 (t; *C*H($CH_3)_2$), 19.5 (p; CH(*C*$H_3)_2$), 19.4 (p; CH(*C*$H_3)_2$), 19.1 (p; CH(*C*$H_3)_2$), 18.1 (p; CH(*C*$H_3)_2$), 10.1 ppm (p; $C_{imi}CH_3$). ^{125}Te NMR (600 MHz, $CDCl_3$): δ = 929.3 ppm. IR (ATR): ṽ = 3375, 2966, 2947, 2870, 1651, 1589, 1504, 1425, 1290, 1114, 1055, 765 cm^{-1}. UV/Vis (MeOH): λ_{max} (log ε) = 240 (4.84), 318 nm (3.85).

HRMS (ESI-TOF) *m*/*z*: $[C_{44}H_{54}N_{10}O_4Te_2+H]^+$ calculated: 1044.2422; found: 1045.2415. $[C_{44}H_{54}N_{10}O_4Te_2+2H]^{2+}$ 523.1291; found: 523.1312.

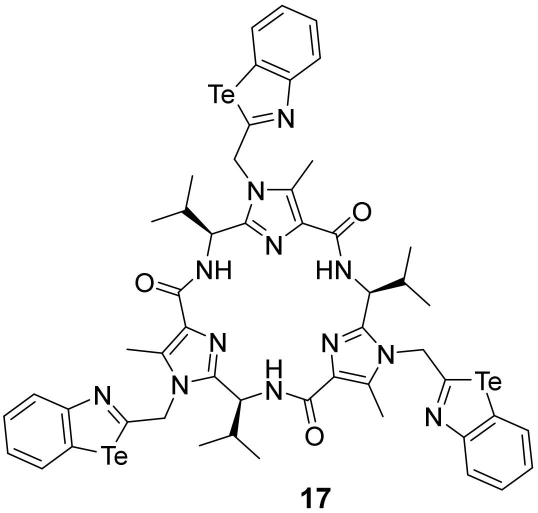

17

Synthesis of cyclic peptide 17. Caesium carbonate (700 mg, 2.15 mmol) was added under argon to a solution of macrocycle **15** (100 mg, 0.20 mmol) in acetonitrile (90 mL). The mixture was stirred for 10 min and then benzotellurazole **13** (581 mg, 1.8 mmol) was added to this solution and stirring was continued at room temperature for 24 h. Then, the solvent was evaporated to dryness, the residue was dissolved in DCM and washed with water. The aqueous layer was saturated with NaCl and then repeatedly extracted with DCM. The organic layers were combined, dried over $MgSO_4$ and concentrated in vacuo. The residue was purified by flash column chromatography with silica gel (DCM/MeOH 99:2 → 99:5) to yield compound **17** (90 mg, 0.07 mmol, 36%) as a yellow solid. ^{1}H NMR (600 MHz, $CDCl_3$) δ = 8.52 (d, $^3J_{H,H}$ = 9.0 Hz, 4 H; NH, NH, NH, NH), 8.11-8.09 (m, 3 H; $C_{ar}H$), 7.77-7.76 (m, 3 H; $C_{ar}H$), 7.47-7.44 (m, 3 H; $C_{ar}H$), 7.16-7.13 (m, 3 H; $C_{ar}H$), 5.34-5.30 (m, 6 H; $CH_2C_{tellurazole}$), 5.29-5.27 (m, 3 H; C*H*NH), 2.54 (m, 9 H; $C_{azol}CH_3$), 2.16-2.11 (m, 3 H; C*H*$(CH_3)_2$), 1.06 (d, $^2J_{H,H}$ = 6.6 Hz, 2 H; $CH(C\mathit{H}_3)_2$), 1.04 ppm (d, $^2J_{H,H}$ = 6.6 Hz, 9 H; $CH(C\mathit{H}_3)_2$). ^{13}C NMR (151 MHz, $CDCl_3$) δ = 171.8 (q; $C_{tellurazole}$), 162.7 (q; CO), 160.6 (q; C_{ar}), 146.8 (q; C_{ar}), 135.0 (q; C_{ar}), 132.1 (q; C_{ar}), 131.6 (q; C_{ar}), 130.7 (q; C_{ar}), 127.0 (t; C_{ar}), 126.6 (t; C_{ar}), 125.2 (t; C_{ar}), 53.1 (s; $CH_2C_{tellurazole}$), 49.7 (t; CHNH), 34.8 (t; *C*H$(CH_3)_2$), 19.8 (p; $CH(\mathit{C}H_3)_2$), 17.2 (p; $CH(\mathit{C}H_3)_2$), 10.3 ppm (p; $\mathit{C}_{imi}\mathit{C}H_3$). ^{125}Te NMR (600 MHz, $CDCl_3$): δ = 931.1 ppm. IR (ATR): $\tilde{\nu}$ = 3381, 2957, 2926, 2870, 1649, 1591, 1504, 1419, 1199, 1053, 1010, 759 cm^{-1}. UV/Vis (MeOH): λ_{max} (log ε) = 241 (4.67), 319 nm (3.66).

HRMS (ESI-TOF) *m*/*z*: $[C_{51}H_{54}N_{12}O_3Te_3+H]^+$ calculated: 1267.1655; found: 1267.1680. $[C_{51}H_{54}N_{12}O_3Te_3+2H]^{2+}$ calculated: 634.0864; found: 634.0873.

4. Computational Details

All calculations were performed by using the program package Gaussian 16[13]. The geometrical parameters of all stationary points were optimized by means of the density functional B3LYP[9-11] and the double-hybrid density functional approximation B2PLYP[12]. In both cases the additional dispersion correction with Becke-Johnson damping[13] (D3BJ) were used. For all structures C1 symmetry was applied. Frequency calculations were carried out at each of the stationary points to verify the nature of the stationary point. It turned out that all stationary states have none imaginary frequency. As basis set TZVP was employed for the light elements C, H, N, O, S and Se, whereas aug-cc-pVTZ-PP was used for tellurium. Furthermore, single point calculations on the B2PLYP-optimized structures were performed using B2PLYP, CCSD[14] and CCSD(T)[15]. As basis sets TZVP and aug-cc-pVTZ-PP were employed. To determine the solvent effect, single point calculations were conducted using water as solvent and both B2PLYP and CCSD as methods.

5. Cartesian Coordinates and Absolute Energies for All Calculated Compounds

Table S4. Absolute energies [au] calculated by means of different methods.

	E[a]	*E*[b]	*E*[c]	*E*[d]
**1a**	-246.1655240	-245.9322280	-245.6795570	-245.6863700
**1b**	-569.1409780	-568.8090013	-568.5857050	-568.5923030
**1c**	-2572.5069960	-2571.9025090	-2571.6839440	-2571.6897340
**1d**	-439.0262260	-438.5066396	-438.2422740	-438.2490090
**2**	-209.3092670	-209.1005766	-208.8837720	-208.8996660
**1a•2** (type I)	-455.4912990	-455.0483852	-454.5774180	-454.5887780
**1b•2** (type I)	-778.4668870	-777.9253407	-777.4834030	-777.4950170
**1c•2** (type I)	-2781.8327680	-2781.0187131	-2780.5814870	-2780.5932320
**1d•2** (type I)	-648.3517040	-647.6225650	-647.1395060	-647.1525310
**1b•2** (type II)	-778.4589640	-777.9176587	-777.4765810	-777.4934520
**1c•2** (type II)	-2781.8262010	-2781.0122223	-2780.5755420	-2780.5918330
**1d•2** (type II)	-648.3479510	-647.6192180	-647.1357960	-647.1526810
**1d•1d** (type III)		-877.0243070		
**1d•1d** (type IV)		-877.0215182		

[a] B3LYP-D3BJ/TZVP,cc-pVTZ.
[b] B2PLYP-D3/TZVP,cc-pVTZ.
[c] B2PLYP-D3/cc-pVTZ//B2PLYP-D3/TZVP,cc-pVTZ.
[d] B2PLYP-D3(water as solvent)/cc-pVTZ//B2PLYP-D3/TZVP,cc-pVTZ.

Table S5. Absolute energies [au] calculated by means of different methods.

	E[a]	*E*[b]	*E*[c]
**1a**	-245.5013641	-245.5381024	-245.5082168
**1b**	-568.1027437	-568.1401869	-568.1092366
**1c**	-2570.4798842	-2570.5204850	-2570.4856405
**1d**	-437.6155222	-437.6592603	-437.6214188
**2**	-208.7421876	-208.7677026	-208.7568429
**1a•2** (type I)	-454.2569611	-454.3200634	-454.2674130
**1b•2** (type I)	-776.8584520	-776.9223770	-776.8689057
**1c•2** (type I)	-2779.2354795	-2779.3025631	-2779.2461764
**1d•2** (type I)	-646.3708743	-646.4410726	-646.3821685
**1b•2** (type II)	-776.8518138	-776.9154451	-776.8677974
**1c•2** (type II)	-2779.2296917	-2779.2966031	-2779.2451749
**1d•2** (type II)	-646.3686221	-646.4391343	-646.3841631
**1d•1d** (type III)		-875.3292957	-875.2471828
**1d•1d** (type IV)		-875.3270795	-875.2505100

[a] CCSD/TZVP,cc-pVTZ//B2PLYP-D3/TZVP,cc-pVTZ.
[b] CCSD(T)/TZVP,cc-pVTZ//B2PLYP-D3/TZVP,cc-pVTZ.
[c] CCSD(water as solvent)/TZVP,cc-pVTZ//B2PLYP-D3/TZVP,cc-pVTZ.

Table S6. Absolute energies [au] calculated by means of different methods.

	E[a]	*E*[b]	*E*[c]	*E*[d]
**3a** (type I)	-647.3214470	-646.6742028	-646.7930476	-646.8097145
**3a** (type III)	-647.3192720	-646.6712950	-646.7893644	-646.8090384
**3b** (type II)	-970.2969880	-969.5521979	-969.6833623	-969.7018110
**3b** (type I)	-970.2973370	-969.5518250	-969.6834244	-969.6996214
**3c** (type II)	-2973.6661770	-2972.6487656	-2972.8017229	-2972.8188441
**3c** (type I)	-2973.6651250	-2972.6470636	-2972.8005046	-2972.8170697
**3d** (type II)	-840.1897830	-839.2578586	-839.3631858	-839.3801056
**3d** (type I)	-840.1861660	-839.2530527	-839.3588671	-839.3766665
**4a** (type I)	-647.3227550	-646.6757538	-646.7938922	-646.8086078
**4a** (type III)	-647.3177820	-646.6726132	-646.7905325	-646.8091030
**4b** (type I)	-970.2983530	-969.5534487	-969.6843277	-969.6988444

**4c** (type II)	-2973.6631520	-2972.6458821	-2972.7986986	-2972.8165112
**4c** (type I)	-2973.6659110	-2972.6484318	-2972.8011495	-2972.8161830
**4d** (type II)	-840.1873270	-839.2550409	-839.3606003	-839.3781093
**4d** (type I)	-840.1864360	-839.2538676	-839.3591399	-839.3752147
**7a** (type II)	-915.4425910	-914.4549994	-914.5735485	-914.5870794
**7a** (type I)	-915.4386180	-914.4499608	-914.5689342	-914.5830827

[a] B3LYP-D3BJ/TZVP,cc-pVTZ.
[b] B2PLYP-D3/TZVP,cc-pVTZ.
[c] B2PLYP-D3/cc-pVTZ//B2PLYP-D3/TZVP,cc-pVTZ.
[d] B2PLYP-D3(water as solvent)/cc-pVTZ//B2PLYP-D3/TZVP,cc-pVTZ.

Table S7. Absolute energies [au] calculated by means of different methods.

	E[a]	*E*[b]	*E*[c]
**3a** (type I)	-645.5048630	-645.6054999	-645.5226387
**3a** (type III)	-645.5024725	-645.6035884	-645.5231470
**3b** (type II)	-968.1077526	-968.2104516	-968.1270146
**3b** (type I)	-968.1073497	-968.2091913	-968.1243746
**3c** (type II)	-2970.4873350	-2970.5932016	-2970.5055918
**3c** (type I)	-2970.4857663	-2970.5906610	-2970.5036267
**3d** (type II)	-837.6280788	-837.7373436	-837.6458035
**3d** (type I)	-837.6226367	-837.7305021	-837.6411622
**4a** (type I)	-645.5054709	-645.6071790	-645.5213901
**4a** (type III)	-645.5030680	-645.6046567	-645.5226403
**4b** (type I)	-968.1082235	-968.2112450	-968.1236582
**4c** (type II)	-2970.4845779	-2970.5904387	-2970.5034711
**4c** (type I)	-2970.4863410	-2970.5924516	-2970.5027747
**4d** (type II)	-837.6247769	-837.7337608	-837.6429870
**4d** (type I)	-837.6226537	-837.7318031	-837.6395737
**7a** (type II)	-912.7120172	-912.8275963	-912.7265984
**7a** (type I)	-912.7066337	-912.8211051	-912.7218361

[a] CCSD/TZVP,cc-pVTZ//B2PLYP-D3/TZVP,cc-pVTZ.
[b] CCSD(T)/TZVP,cc-pVTZ//B2PLYP-D3/TZVP,cc-pVTZ.
[c] CCSD(water as solvent)/TZVP,cc-pVTZ//B2PLYP-D3/TZVP,cc-pVTZ.

Cartesian coordinates of the optimized geometry for **1a** at B3LYP-D3BJ/TZVP,cc-pVTZ level of theory (number of imaginary frequencies = 0):

N	0.42184400	-1.13616200	-0.00001200
C	1.10340400	-0.04261200	0.00001100
C	-0.90588000	-0.71891600	0.00000800
C	-0.94405900	0.63131900	-0.00000300
H	2.17255100	0.08482300	0.00000000
H	-1.72044900	-1.42238000	0.00000900
H	-1.72005900	1.37423800	-0.00000800
O	0.34928200	1.08721300	-0.00000200

Cartesian coordinates of the optimized geometry for **1a** at B2PLYP-D3/TZVP,cc-pVTZ level of theory (number of imaginary frequencies = 0):

N	-0.41799100	1.14093700	-0.00001100
C	-1.10144200	0.04588900	0.00001000
C	0.90780200	0.71640000	0.00000800
C	0.94083600	-0.63490200	-0.00000100
H	-2.16933900	-0.07901200	0.00000800
H	1.72480300	1.41479400	0.00001300
H	1.71332900	-1.37971300	-0.00000300
O	-0.35325500	-1.08836900	-0.00000500

Cartesian coordinates of the optimized geometry for **1b** at B3LYP-D3BJ/TZVP,cc-pVTZ level of theory (number of imaginary frequencies = 0):

N	-1.25273900	-0.78241200	0.00000100
C	-0.03085100	-1.20894300	0.00000000
C	-1.29207000	0.59252900	-0.00000100
C	-0.08595400	1.22073900	0.00000000
H	0.25070300	-2.25167800	-0.00000100
H	-2.25000800	1.09112300	0.00000000
H	0.12353800	2.27723900	0.00000200
S	1.19363700	0.04589000	0.00000000

Cartesian coordinates of the optimized geometry for **1b** at B2PLYP-D3/TZVP,cc-pVTZ level of theory (number of imaginary frequencies = 0):

N	1.25883900	0.77929400	0.00000000
C	0.03182400	1.20598200	-0.00000100
C	1.28977500	-0.59559500	0.00000000
C	0.07859100	-1.21943900	0.00000100
H	-0.24867200	2.24744500	-0.00000100
H	2.24357100	-1.09883400	0.00000000
H	-0.13463000	-2.27421700	0.00000100
S	-1.19208000	-0.04219600	0.00000000

Cartesian coordinates of the optimized geometry for **1c** at B3LYP-D3BJ/TZVP,cc-pVTZ level of theory (number of imaginary frequencies = 0):

N	-1.68491100	-0.75120200	-0.00000600
C	-0.50194900	-1.25879700	0.00000900
C	-1.68279700	0.62662500	0.00000100
C	-0.48278700	1.25958100	0.00000600
H	-0.29844600	-2.32020300	-0.00000200
H	-2.63530700	1.13768500	-0.00001200
H	-0.29899200	2.32126400	0.00000000
Se	0.91271500	0.01044700	-0.00000100

Cartesian coordinates of the optimized geometry for **1c** at B2PLYP-D3/TZVP,cc-pVTZ level of theory (number of imaginary frequencies = 0):

N	1.68768100	-0.75006100	0.00002100
C	0.49725600	-1.25519500	0.00005800
C	1.68057400	0.62624700	0.00002500
C	0.47612300	1.25834500	0.00005400
H	0.29291700	-2.31507900	0.00006100
H	2.63008900	1.14023900	0.00002300
H	0.29141200	2.31916800	0.00007100
Se	-0.91035000	0.00969800	-0.00003300

Cartesian coordinates of the optimized geometry for **1d** at B3LYP-D3BJ/TZVP,cc-pVTZ level of theory (number of imaginary frequencies = 0):

Te	0.77900100	0.00431100	0.00003000
N	-2.00714000	-0.73973200	0.00008700
C	-0.86232000	-1.31810100	-0.00020800
C	-1.99333200	0.63986200	0.00016300
C	-0.81830100	1.31530200	-0.00023600
H	-0.74594200	-2.39395500	-0.00030800
H	-2.95541200	1.13672300	0.00026700
H	-0.71301200	2.38880000	-0.00042000

Cartesian coordinates of the optimized geometry for **1d** at B2PLYP-D3/TZVP,cc-pVTZ level of theory (number of imaginary frequencies = 0):

Te	-0.77616400	0.00397400	-0.00006500
N	2.00779600	-0.73854000	-0.00010300
C	0.85447100	-1.31589700	0.00033800
C	1.98962700	0.63838800	-0.00009900
C	0.80931700	1.31518100	0.00033200
H	0.73486400	-2.39012400	0.00038700
H	2.94829700	1.13911900	-0.00024200
H	0.70233700	2.38812400	0.00050800

Cartesian coordinates of the optimized geometry for **2** at B3LYP-D3BJ/TZVP,cc-pVTZ level of theory (number of imaginary frequencies = 0):

O	0.35399300	1.33096200	0.00000200
C	0.07561600	0.14638500	-0.00003000
N	1.03376800	-0.82562600	-0.00001000
C	-1.35930500	-0.34594800	-0.00000200
H	-1.45155900	-1.43231500	-0.00091500
H	-1.86304200	0.05480300	0.87991700
H	-1.86362500	0.05637500	-0.87885500
H	2.00099000	-0.54515000	0.00010500
H	0.81105000	-1.80465200	-0.00000900

Cartesian coordinates of the optimized geometry for **2** at B2PLYP-D3/TZVP,cc-pVTZ level of theory (number of imaginary frequencies = 0):

O	0.36031700	1.33166000	0.00258000
C	0.07522700	0.14627000	-0.00312100
N	1.02866300	-0.83127600	-0.01640200
C	-1.35936700	-0.34095600	-0.00000100
H	-1.45244100	-1.41820500	-0.12795800
H	-1.82093400	-0.05344000	0.94383100
H	-1.89752200	0.16403500	-0.79927200
H	1.99367300	-0.55357900	0.04293500
H	0.79888800	-1.80504000	0.05337600

Cartesian coordinates of the optimized geometry for **1a•2** (type I) at B3LYP-D3BJ/TZVP,cc-pVTZ level of theory (number of imaginary frequencies = 0):

N	-1.23018700	0.67537800	0.00013100
C	-1.27594500	-0.61805300	0.00029400
C	-2.55988300	1.08532300	-0.00008200
C	-3.34973600	-0.00913900	-0.00018200
O	1.75994700	-1.14407700	-0.00023600
C	2.37112600	-0.08116900	0.00002500
N	1.75012400	1.12123000	-0.00007600
H	-0.43672400	-1.29786400	0.00058900
H	-2.83678300	2.12521200	-0.00015400
H	-4.40922600	-0.18620800	-0.00046200
C	3.88790100	-0.05458200	0.00002700
H	4.30829000	0.95147600	-0.00042100
H	4.24523100	-0.59117600	0.87937700
H	4.24497700	-0.59179000	-0.87906700
H	0.73053500	1.16379000	0.00015500
H	2.27741300	1.97588300	0.00046300
O	-2.53545200	-1.11340700	0.00006700

Cartesian coordinates of the optimized geometry for **1a•2** (type I) at B2PLYP-D3/TZVP,cc-pVTZ level of theory (number of imaginary frequencies = 0):

N	-1.23490700	0.67544400	0.00022000

C	-1.29012700	-0.61968100	0.00054100
C	-2.56405200	1.08747800	-0.00006200
C	-3.35850300	-0.00500000	0.00008700
O	1.76695500	-1.14482100	-0.00075100
C	2.38300800	-0.08252700	-0.00088000
N	1.76500100	1.12215100	-0.00016400
H	-0.45813900	-1.30534300	0.00087000
H	-2.83842900	2.12672600	-0.00033900
H	-4.41763200	-0.17741800	-0.00000900
C	3.89800000	-0.05746200	0.00018400
H	4.31573700	0.94820300	0.00041100
H	4.25311500	-0.59263500	0.87931800
H	4.25421800	-0.59272700	-0.87843200
H	0.74778900	1.16383400	0.00015000
H	2.29363700	1.97456800	0.00070400
O	-2.55081900	-1.11333100	0.00046400

Cartesian coordinates of the optimized geometry for **1b•2** (type I) at B3LYP-D3BJ/TZVP,cc-pVTZ level of theory (number of imaginary frequencies = 0):

N	-0.85234800	0.83145600	0.00006900
C	-0.95389200	-0.46486500	0.00021100
C	-2.08977800	1.43405300	-0.00012600
C	-3.15232100	0.58770500	-0.00017300
O	2.07840100	-1.15779400	-0.00026900
C	2.72390200	-0.11533100	-0.00004600
N	2.14323700	1.10695000	0.00014000
H	-0.09153600	-1.12189800	0.00027600
H	-2.15239400	2.51201300	-0.00022000
H	-4.20276600	0.82516900	-0.00032900
C	4.24080800	-0.13910300	0.00000400
H	4.69447600	0.85240400	-0.00005800
H	4.57997800	-0.68750300	0.87927400
H	4.58002800	-0.68764500	-0.87915300
H	1.12492100	1.18624500	0.00008400
H	2.69985800	1.94278900	0.00033400
S	-2.59426900	-1.05892600	0.00007900

Cartesian coordinates of the optimized geometry for **1b•2** (type I) at B2PLYP-D3/TZVP,cc-pVTZ level of theory (number of imaginary frequencies = 0):

N	-0.85828500	0.83450900	0.00005900
C	-0.96655300	-0.46588800	0.00023000
C	-2.09631400	1.43342700	-0.00017000
C	-3.16062300	0.58476700	-0.00020700
O	2.08698000	-1.15881400	-0.00025600
C	2.73656400	-0.11665500	0.00006900
N	2.15873200	1.10773200	0.00021900
H	-0.10896500	-1.12623100	0.00047900
H	-2.16218700	2.51007400	-0.00028700
H	-4.20988300	0.82387200	-0.00035800
C	4.25172600	-0.14097600	-0.00001600
H	4.70215300	0.85043000	-0.00001500
H	4.58971700	-0.68754300	0.87880900
H	4.58963700	-0.68751100	-0.87888900
H	1.14298400	1.18775100	0.00022700
H	2.71699400	1.94109400	0.00035800
S	-2.60301300	-1.05657300	0.00002100

Cartesian coordinates of the optimized geometry for **1c•2** (type I) at B3LYP-D3BJ/TZVP,cc-pVTZ level of theory (number of imaginary frequencies = 0):

N	-0.22884700	1.06371800	-0.00002700

C	-0.42462500	-0.21506700	-0.00015300
C	-1.39300900	1.80278600	-0.00012900
C	-2.56658700	1.12442300	-0.00048400
O	2.54308300	-1.16387900	-0.00027100
C	3.26758300	-0.17471000	-0.00020900
N	2.78442500	1.08928600	-0.00008200
H	0.39027700	-0.93115300	-0.00017700
H	-1.31553400	2.88089300	-0.00022800
H	-3.56287400	1.53443300	-0.00082500
C	4.77806100	-0.31657900	-0.00016500
H	5.30767300	0.63652100	-0.00020000
H	5.07340300	-0.88978500	0.87909100
H	5.07343600	-0.88987800	-0.87934600
H	1.77596300	1.25047700	-0.00004100
H	3.40532000	1.87858800	-0.00000200
Se	-2.24558600	-0.72221200	0.00033800

Cartesian coordinates of the optimized geometry for **1c•2** (type I) at B2PLYP-D3/TZVP,cc-pVTZ level of theory (number of imaginary frequencies = 0):

N	0.23462200	1.06722900	-0.00001300
C	0.43590300	-0.21645800	-0.00003500
C	1.40014600	1.80039500	0.00001800
C	2.57536400	1.11786000	-0.00000100
O	-2.55279300	-1.16421700	-0.00009700
C	-3.28077100	-0.17529800	-0.00010300
N	-2.79905200	1.09005900	-0.00003700
H	-0.37561500	-0.93382500	-0.00006300
H	1.32770900	2.87780300	0.00001600
H	3.57118200	1.52722400	-0.00000700
C	-4.78950100	-0.31635800	-0.00014100
H	-5.31506400	0.63740900	-0.00001300
H	-5.08433900	-0.88724400	-0.87910400
H	-5.08436800	-0.88750000	0.87864600
H	-1.79264900	1.24892500	-0.00001700
H	-3.41986400	1.87799300	-0.00003900
Se	2.24998600	-0.72085000	0.00009600

Cartesian coordinates of the optimized geometry for **1d•2** (type I) at B3LYP-D3BJ/TZVP,cc-pVTZ level of theory (number of imaginary frequencies = 0):

N	0.27808800	1.16827300	0.00007200
C	0.02131300	-0.09522300	0.00007700
C	-0.80781500	2.02181200	-0.00001400
C	-2.06662600	1.52280800	-0.00004500
O	2.95174200	-1.19658700	0.00007900
C	3.71867400	-0.24008400	0.00012600
N	3.29107300	1.04390700	0.00009700
H	0.81381200	-0.83756900	0.00013300
H	-0.59610700	3.08367200	-0.00003100
H	-2.97202700	2.10881100	-0.00009800
C	5.22157300	-0.44756700	0.00010900
H	5.79223600	0.48155000	-0.00004900
H	5.49175200	-1.03298300	0.87941700
H	5.49170300	-1.03326300	-0.87902500
H	2.29050400	1.24923700	0.00009100
H	3.94582700	1.80535600	0.00009600
Te	-2.02650900	-0.54438200	-0.00007400

Cartesian coordinates of the optimized geometry for **1d•2** (type I) at B2PLYP-D3/TZVP,cc-pVTZ level of theory (number of imaginary frequencies = 0):

N	0.27004400	1.17375300	0.00001600

C	0.01112200	-0.09599900	0.00003100
C	-0.81858000	2.01949800	-0.00001300
C	-2.08023300	1.51430500	-0.00002000
O	2.96018100	-1.19502400	0.00006000
C	3.73109300	-0.23930300	0.00003400
N	3.30576500	1.04627600	0.00000700
H	0.80149800	-0.83854700	0.00006000
H	-0.61461100	3.08188700	-0.00001100
H	-2.98730500	2.09702600	-0.00002800
C	5.23218200	-0.44666400	0.00014000
H	5.79930800	0.48299100	0.00002500
H	5.50153300	-1.02996400	0.87913100
H	5.50160700	-1.03023600	-0.87864500
H	2.30741400	1.24986800	0.00000700
H	3.96079700	1.80601400	0.00004100
Te	-2.02761300	-0.54442500	-0.00004300

Cartesian coordinates of the optimized geometry for **1b•2** (type II) at B3LYP-D3BJ/TZVP,cc-pVTZ level of theory (number of imaginary frequencies = 0):

N	-2.32908100	1.33477600	-0.49157600
C	-1.21292300	0.97524200	0.05302200
C	-3.23003900	0.29271800	-0.51079100
C	-2.79335800	-0.87784700	0.02438500
O	1.80218700	0.61924900	0.73356400
C	2.56485600	0.02648600	-0.01404100
N	2.13726700	-0.94792000	-0.86040800
H	-0.34144800	1.59594600	0.18971400
H	-4.20912900	0.45320900	-0.93778900
H	-3.32403100	-1.81050100	0.11962800
C	4.04723100	0.33603800	-0.05017500
H	4.60275900	-0.26535600	-0.76992000
H	4.17572600	1.39167700	-0.29030100
H	4.45936700	0.17346300	0.94604000
H	1.15757900	-1.18798100	-0.86554500
H	2.76145000	-1.43249800	-1.47990000
S	-1.16323000	-0.69348600	0.60431500

Cartesian coordinates of the optimized geometry for **1b•2** (type II) at B2PLYP-D3/TZVP,cc-pVTZ level of theory (number of imaginary frequencies = 0):

N	2.34462900	1.34492500	0.48483800
C	1.22344200	0.96913200	-0.05087200
C	3.24676400	0.30527600	0.50466800
C	2.80804700	-0.87363800	-0.01721000
O	-1.79358500	0.60148200	-0.72571600
C	-2.58425300	0.02022500	0.00523300
N	-2.20309600	-1.00540500	0.81340900
H	0.35137200	1.58653300	-0.18901000
H	4.22751200	0.47036600	0.92302400
H	3.34209600	-1.80376100	-0.10938200
C	-4.04830500	0.39783000	0.06010200
H	-4.63885000	-0.24836900	0.70734600
H	-4.12828600	1.42533900	0.41191400
H	-4.45235800	0.36133000	-0.94968500
H	-1.23007300	-1.26478600	0.83254700
H	-2.83980300	-1.45187600	1.44685100
S	1.17826000	-0.69851400	-0.58544600

Cartesian coordinates of the optimized geometry for **1c•2** (type II) at B3LYP-D3BJ/TZVP,cc-pVTZ level of theory (number of imaginary frequencies = 0):

N	-1.99003700	1.71627000	0.10647800

C	-0.90819900	1.10537900	0.43998800
C	-2.96637200	0.86809300	-0.37585200
C	-2.67855100	-0.45599900	-0.43945800
O	1.99262200	0.03061400	0.91014600
C	2.75836600	0.10449900	-0.03985300
N	2.32433700	0.04485800	-1.32620900
H	-0.02199900	1.57092300	0.84372200
H	-3.91002300	1.30015000	-0.67899200
H	-3.32510700	-1.24601000	-0.78545200
C	4.25126700	0.26920500	0.15318500
H	4.80848800	0.33578300	-0.78131000
H	4.42820000	1.16992200	0.74126400
H	4.62122300	-0.57801300	0.73125900
Se	-0.93855900	-0.77953400	0.18722700
H	1.33777600	-0.07523500	-1.50001800
H	2.95231900	0.10677700	-2.10730300

Cartesian coordinates of the optimized geometry for **1c•2** (type II) at B2PLYP-D3/TZVP,cc-pVTZ level of theory (number of imaginary frequencies = 0):

N	1.98860300	1.72615900	-0.07228500
C	0.90703000	1.10566800	-0.41210900
C	2.97054500	0.87427100	0.38673800
C	2.69112600	-0.45615600	0.42500400
O	-1.99623100	0.05212400	-0.89957500
C	-2.78034200	0.09925600	0.03987700
N	-2.37181700	-0.01978000	1.33103000
H	0.01701100	1.57098500	-0.80433500
H	3.91207100	1.30427700	0.69511100
H	3.34487200	-1.24771200	0.75112900
C	-4.26610300	0.28967200	-0.17085900
H	-4.83543700	0.31854300	0.75658600
H	-4.42248200	1.21819700	-0.71707900
H	-4.63382700	-0.52537800	-0.79184100
Se	0.95697600	-0.77561000	-0.19809100
H	-1.38964700	-0.14435800	1.51713700
H	-3.01091300	0.03825900	2.10184900

Cartesian coordinates of the optimized geometry for **1d•2** (type II) at B3LYP-D3BJ/TZVP,cc-pVTZ level of theory (number of imaginary frequencies = 0):

N	-1.72300700	1.94333200	0.45713500
C	-0.65513400	1.25105600	0.62115000
C	-2.79567900	1.27587200	-0.10581900
C	-2.68384900	-0.03129900	-0.44690600
O	2.10852000	-0.38284800	0.72449700
C	2.89227100	0.18030400	-0.02971600
N	2.47777300	0.86767000	-1.12327900
H	0.25087100	1.64247800	1.06444300
H	-3.70156200	1.85151700	-0.25043900
H	-3.47453400	-0.61751600	-0.89006800
C	4.38496000	0.14602800	0.21509300
H	4.95998900	0.71440900	-0.51582000
H	4.58258600	0.53894800	1.21244500
H	4.71450900	-0.89330100	0.20054500
H	1.49122600	0.90010200	-1.33470700
H	3.12260200	1.32132400	-1.74535600
Te	-0.78755000	-0.75007600	-0.00763100

Cartesian coordinates of the optimized geometry for **1d•2** (type II) at B2PLYP-D3/TZVP,cc-pVTZ level of theory (number of imaginary frequencies = 0):

N	1.78988600	1.91404300	-0.46141100

C	0.69982200	1.24563700	-0.63374000
C	2.82995900	1.21795600	0.12151500
C	2.67259000	-0.08713800	0.47219700
O	-2.09394300	-0.30744300	-0.76261700
C	-2.89116700	0.21359700	0.01141500
N	-2.48895800	0.96709300	1.06656600
H	-0.18745100	1.65590500	-1.09528000
H	3.75119900	1.76391100	0.27591100
H	3.43748200	-0.69411800	0.93130100
C	-4.38385000	0.05234700	-0.16272000
H	-4.96677200	0.66937100	0.51882100
H	-4.64205600	0.30394200	-1.18909700
H	-4.63948900	-0.99455000	-0.00334300
H	-1.50229300	1.04642200	1.25798300
H	-3.14219000	1.34433600	1.72817400
Te	0.76870200	-0.74342300	0.01131500

Cartesian coordinates of the optimized geometry for **1d•1d** (type III) at B2PLYP-D3/TZVP,cc-pVTZ level of theory (number of imaginary frequencies = 0):

N	3.30412100	1.94869100	0.56510000
C	2.18533900	1.31043500	0.50799200
C	4.41602800	1.24257600	0.14893600
C	4.29299100	-0.04072000	-0.28411500
H	1.23859000	1.73917400	0.80465900
H	5.36310500	1.76424300	0.18999100
H	5.11373000	-0.65331900	-0.62400300
Te	2.31931800	-0.65185900	-0.20470100
C	-1.38418200	0.23797000	-0.66107500
C	-2.69859100	-0.62882600	1.44854300
C	-1.34325100	-0.72387400	1.39221600
N	-0.66195000	-0.26774000	0.28551000
H	-0.95490200	0.62525300	-1.57482500
H	-3.31620600	-0.95591300	2.26959000
H	-0.74937700	-1.14830500	2.19039100
Te	-3.43470600	0.23795200	-0.26693500

Cartesian coordinates of the optimized geometry for **1d•1d** (type IV) at B2PLYP-D3/TZVP,cc-pVTZ level of theory (number of imaginary frequencies = 0):

N	1.85185700	1.71525200	1.37303400
C	1.74417200	0.43822200	1.50248500
C	2.22317000	2.16662400	0.12560100
C	2.45103200	1.28754000	-0.88767200
H	1.45234000	-0.03941700	2.42716400
H	2.31964200	3.23683600	0.00168600
H	2.74363800	1.55094000	-1.89164100
Te	2.17302800	-0.64920300	-0.24554300
C	-1.41911000	1.24998700	-0.44569400
C	-3.67567300	1.31092900	-0.29698000
C	-3.68056500	0.00147000	0.06944000
H	-0.42579000	1.63663400	-0.62381700
H	-4.58104700	1.89512500	-0.39317100
H	-4.56121800	-0.57795700	0.29727200
Te	-1.75671400	-0.73827000	0.13271500
N	-2.48815500	1.95872100	-0.56639600

Cartesian coordinates of the optimized geometry for **3a** (type I) at B3LYP-D3BJ/TZVP,cc-pVTZ level of theory (number of imaginary frequencies = 0):

O	-0.81834100	-1.42366400	-0.00027000
N	-0.27676900	0.75840500	-0.00110700
C	0.15240700	-0.45604600	-0.00102200

C	-1.67254700	0.64401500	-0.00038100
C	-2.67876100	1.60305300	-0.00011900
H	-2.44159300	2.65816700	-0.00049100
C	-3.99247200	1.14672300	0.00061900
H	-4.80331200	1.86348000	0.00083800
C	-4.29541600	-0.22173400	0.00108500
H	-5.33154100	-0.53390800	0.00166200
C	-3.29519200	-1.19183300	0.00083200
H	-3.51767000	-2.24969300	0.00119500
C	-2.00074500	-0.71379400	0.00009600
C	1.56435800	-0.91843700	-0.00166900
H	1.74727200	-1.55073000	-0.87658700
H	1.74809000	-1.55109600	0.87279300
N	2.44033600	0.23026300	-0.00177700
C	3.79094900	0.07550500	0.00048100
H	2.00539200	1.14023500	-0.00180700
O	4.31226900	-1.02904000	0.00184700
C	4.59860100	1.35638200	0.00109100
H	5.24149500	1.36268300	0.88172600
H	5.24417000	1.36188200	-0.87758100
H	3.98420600	2.25692600	-0.00026000

Cartesian coordinates of the optimized geometry for **3a** (type I) at B2PLYP-D3/TZVP,cc-pVTZ level of theory (number of imaginary frequencies = 0):

O	-0.81421600	-1.42478500	-0.02224500
N	-0.27203200	0.76250700	-0.06375300
C	0.15331700	-0.45624900	-0.06072500
C	-1.66674700	0.64392200	-0.02555800
C	-2.67242800	1.60430000	0.01761400
H	-2.43640100	2.65864600	-0.00428600
C	-3.98555500	1.14705600	0.03828600
H	-4.79575200	1.86243000	0.07048600
C	-4.28740900	-0.22285300	0.07241200
H	-5.32263700	-0.53444900	0.09130200
C	-3.28878000	-1.19310900	0.03076800
H	-3.51010300	-2.25009200	0.05303200
C	-1.99361300	-0.71226400	0.00879700
C	1.56433900	-0.92148100	-0.10949800
H	1.71170500	-1.55050800	-0.99176700
H	1.78138100	-1.54940200	0.75798800
N	2.43868200	0.22791500	-0.13383000
C	3.78032000	0.07642100	0.03352900
H	2.00535300	1.13729600	-0.16609600
O	4.29426000	-1.02781100	0.15450700
C	4.58591500	1.35654200	0.05074300
H	5.11072500	1.42444200	1.00241600
H	5.33473200	1.30672800	-0.73791700
H	3.97793200	2.24902400	-0.08838800

Cartesian coordinates of the optimized geometry for **3b** (type I) at B3LYP-D3BJ/TZVP,cc-pVTZ level of theory (number of imaginary frequencies = 0):

S	0.92703000	-1.85874900	-0.00002500
N	0.16165800	0.63453900	0.00086400
C	-0.29574900	-0.56812100	0.00075300
C	1.54921900	0.65956200	0.00033100
C	2.33792200	1.81023300	0.00028400
H	1.86166900	2.78154100	0.00068600
C	3.71695400	1.67679200	-0.00027600
H	4.34051800	2.56131100	-0.00031500
C	4.31686900	0.41161300	-0.00079000
H	5.39623400	0.33147500	-0.00122400
C	3.54896300	-0.74462400	-0.00075300

H	4.01646000	-1.72016900	-0.00115000
C	2.16526500	-0.60866400	-0.00018600
C	-1.75358800	-0.90722900	0.00146100
H	-1.99594400	-1.51885800	0.87663600
H	-1.99658000	-1.52069500	-0.87220900
N	-2.53545300	0.30694900	0.00034500
C	-3.89292900	0.26424400	-0.00054600
H	-2.02260700	1.17552700	0.00056000
O	-4.50637500	-0.79268100	-0.00041800
C	-4.59184100	1.60808000	-0.00103200
H	-5.23481200	1.66649900	-0.87970200
H	-5.23182800	1.66871200	0.87970200
H	-3.90453100	2.45435400	-0.00317900

Cartesian coordinates of the optimized geometry for **3b** (type I) at B2PLYP-D3/TZVP,cc-pVTZ level of theory (number of imaginary frequencies = 0):

S	0.91420200	-1.85239400	-0.02352500
N	0.16113500	0.64144900	0.13208000
C	-0.29717100	-0.56578300	0.10330000
C	1.54666800	0.65755000	0.05807000
C	2.33885800	1.80780200	0.04145700
H	1.86899900	2.77878500	0.11321000
C	3.71598600	1.66939300	-0.01595200
H	4.34228200	2.55086400	-0.02590500
C	4.30890500	0.40240200	-0.11016400
H	5.38625500	0.31923000	-0.15475600
C	3.53833800	-0.75149100	-0.09423400
H	3.99996000	-1.72634500	-0.16682600
C	2.15345400	-0.60996500	-0.04640200
C	-1.75406400	-0.90257700	0.19017100
H	-1.94746500	-1.45649700	1.11398700
H	-2.04394300	-1.56124200	-0.63133100
N	-2.53468300	0.31122400	0.14389600
C	-3.87910000	0.26474900	-0.05095300
H	-2.02701300	1.18119600	0.17941600
O	-4.48301600	-0.79579700	-0.15210600
C	-4.57467700	1.60623300	-0.12777600
H	-5.07484200	1.68408400	-1.09187600
H	-5.33910600	1.64657800	0.64640500
H	-3.89657700	2.44943900	-0.00600400

Cartesian coordinates of the optimized geometry for **3c** (type I) at B3LYP-D3BJ/TZVP,cc-pVTZ level of theory (number of imaginary frequencies = 0):

Se	0.91394500	-1.69837100	-0.00010600
N	-0.02142700	0.86769200	0.00143300
C	-0.44933800	-0.33955600	0.00127900
C	1.36288400	0.98262200	0.00054600
C	2.03976700	2.20314200	0.00051400
H	1.46874600	3.12203300	0.00120800
C	3.42528900	2.21066300	-0.00040100
H	3.95650400	3.15354300	-0.00043100
C	4.14581300	1.01198400	-0.00128900
H	5.22798500	1.03581000	-0.00199900
C	3.49084800	-0.21255300	-0.00127200
H	4.05459800	-1.13595900	-0.00196200
C	2.10109000	-0.21920200	-0.00035300
C	-1.90015000	-0.70775700	0.00235900
H	-2.12816500	-1.32531700	0.87722300
H	-2.12947300	-1.32659200	-0.87121200
N	-2.71203600	0.48707700	0.00186900
C	-4.06777900	0.41198600	-0.00064400
H	-2.21996800	1.36749400	0.00236200

O	-4.65622400	-0.65929800	-0.00211900
C	-4.79909300	1.73843700	-0.00131400
H	-5.44193900	1.78186400	-0.88094200
H	-5.44172400	1.78286200	0.87845100
H	-4.13264700	2.60128200	-0.00180200

Cartesian coordinates of the optimized geometry for **3c** (type I) at B2PLYP-D3/TZVP,cc-pVTZ level of theory (number of imaginary frequencies = 0):

Se	0.90335500	-1.69281600	-0.01463200
N	-0.02089700	0.87472700	0.10462300
C	-0.44908700	-0.33767800	0.08723400
C	1.36283700	0.97979200	0.04829100
C	2.04393400	2.19985900	0.02849300
H	1.47873100	3.11987300	0.08393800
C	3.42872500	2.20121400	-0.01064400
H	3.96338100	3.14107000	-0.02293400
C	4.14311700	0.99900900	-0.08565900
H	5.22406100	1.01901400	-0.11688500
C	3.48493500	-0.22329100	-0.06605100
H	4.04311200	-1.14753200	-0.12206700
C	2.09282500	-0.22369000	-0.03311700
C	-1.89949200	-0.70454400	0.15842500
H	-2.08731700	-1.27711100	1.07179700
H	-2.16714700	-1.35797700	-0.67510300
N	-2.70877500	0.49118300	0.12612900
C	-4.05540300	0.41334700	-0.04017600
H	-2.22050000	1.37239300	0.15301500
O	-4.63627900	-0.66122900	-0.12844900
C	-4.78385200	1.73810400	-0.10291900
H	-5.30397600	1.80437400	-1.05729100
H	-5.53410200	1.76032000	0.68572500
H	-4.12360100	2.59706300	0.00634700

Cartesian coordinates of the optimized geometry for **3d** (type I) at B3LYP-D3BJ/TZVP,cc-pVTZ level of theory (number of imaginary frequencies = 0):

Te	0.96609900	-1.60420200	-0.00016000
N	-0.22660400	1.04665300	0.00249600
C	-0.60978200	-0.17052800	0.00239300
C	1.14333600	1.28849800	0.00093900
C	1.65573000	2.58809300	0.00088700
H	0.96420200	3.42030100	0.00208100
C	3.02654800	2.78864400	-0.00067000
H	3.42198500	3.79609300	-0.00070600
C	3.90135000	1.69977200	-0.00219400
H	4.97101400	1.86666100	-0.00340400
C	3.41164300	0.39972000	-0.00216800
H	4.09874900	-0.43629500	-0.00335500
C	2.03622900	0.19348100	-0.00060800
C	-2.05190900	-0.57473700	0.00432200
H	-2.25987100	-1.19954000	0.87922900
H	-2.26217800	-1.20187200	-0.86827100
N	-2.90245000	0.59312800	0.00348100
C	-4.25515400	0.47688900	-0.00112300
H	-2.43759800	1.48812000	0.00427500
O	-4.81153400	-0.61145200	-0.00374700
C	-5.02646700	1.78083600	-0.00256000
H	-5.66923700	1.80498100	-0.88300000
H	-5.67126700	1.80571000	0.87640600
H	-4.38644900	2.66349400	-0.00208800

Cartesian coordinates of the optimized geometry for **3d** (type I) at B2PLYP-D3/TZVP,cc-pVTZ level of theory (number of imaginary frequencies = 0):

Te	0.95762100	-1.59906100	-0.00061800
N	-0.22404800	1.05458700	0.00404600
C	-0.60878800	-0.16813700	0.00362400
C	1.14746600	1.28516500	0.00380500
C	1.66691600	2.58416800	-0.00649200
H	0.98029600	3.41960200	-0.00022600
C	3.03871700	2.77623000	0.00565300
H	3.43994800	3.78045300	-0.00225000
C	3.90924100	1.68128200	-0.00723300
H	4.97877000	1.84355100	0.00032400
C	3.41461800	0.38349400	0.00319700
H	4.09623300	-0.45612900	-0.00143500
C	2.03476700	0.18546100	-0.00325100
C	-2.05239100	-0.57141500	0.00511800
H	-2.26044600	-1.19325400	0.88042400
H	-2.26225500	-1.19431500	-0.86894200
N	-2.89747200	0.59996900	0.00491100
C	-4.25072700	0.48008100	-0.00115900
H	-2.43462900	1.49510000	0.00608900
O	-4.80377600	-0.61290200	-0.00502000
C	-5.02333100	1.78130200	-0.00251600
H	-5.66129000	1.80635400	-0.88453700
H	-5.66867200	1.80380100	0.87418600
H	-4.38233200	2.66156100	0.00148600

Cartesian coordinates of the optimized geometry for **3b** (type II) at B3LYP-D3BJ/TZVP,cc-pVTZ level of theory (number of imaginary frequencies = 0):

S	-0.01006400	-0.99630000	-0.60737800
N	0.64579000	1.50500500	-0.22176100
C	-0.34786800	0.75109200	-0.54555100
C	1.79378000	0.76370500	0.01108100
C	3.03445200	1.29112600	0.37479400
H	3.14227600	2.36128800	0.49118800
C	4.09683000	0.42632500	0.57677700
H	5.06423500	0.82165600	0.85846100
C	3.93728500	-0.95702400	0.41962700
H	4.78227700	-1.61400100	0.58150200
C	2.71282900	-1.49978900	0.05848600
H	2.59511000	-2.56847900	-0.06268500
C	1.64407100	-0.63114400	-0.14391200
C	-1.72678200	1.27575900	-0.82193600
H	-2.12016900	0.84960200	-1.74401300
H	-1.65197800	2.35658300	-0.92946900
N	-2.67088000	0.96079000	0.23849700
C	-3.38066500	-0.20619200	0.23251100
H	-2.63957500	1.51437100	1.07877800
O	-3.32182000	-0.99372600	-0.69774200
C	-4.24317100	-0.45640500	1.45030000
H	-5.24980900	-0.70915200	1.11888000
H	-3.84481000	-1.31988100	1.98537100
H	-4.29090300	0.39333400	2.13177500

Cartesian coordinates of the optimized geometry for **3b** (type II) at B2PLYP-D3/TZVP,cc-pVTZ level of theory (number of imaginary frequencies = 0):

S	-0.02020800	-0.98986400	-0.61884800
N	0.63612200	1.51190500	-0.22412100
C	-0.35677100	0.75078400	-0.55625600
C	1.77976900	0.76523900	0.00974700
C	3.01519000	1.29038400	0.40061000
H	3.12449300	2.35968900	0.51707000

C	4.08147000	0.42597000	0.58091500
H	5.04407600	0.81763200	0.88030300
C	3.91959200	-0.95917100	0.42538900
H	4.76766100	-1.61467500	0.57055600
C	2.70253600	-1.49809100	0.03589200
H	2.58386700	-2.56598000	-0.08513300
C	1.62530600	-0.62929300	-0.13586000
C	-1.73218300	1.28171400	-0.83574900
H	-2.12709300	0.85978000	-1.75789700
H	-1.65033000	2.36141300	-0.93952900
N	-2.67788300	0.97571300	0.22482500
C	-3.34922800	-0.21467800	0.24240400
H	-2.64306700	1.53185300	1.06281600
O	-3.26403200	-1.01815100	-0.67591700
C	-4.20464300	-0.46605800	1.46292200
H	-5.19375300	-0.77902500	1.13541900
H	-3.76643200	-1.28670500	2.03043700
H	-4.29772200	0.40491100	2.10985000

Cartesian coordinates of the optimized geometry for **3c** (type II) at B3LYP-D3BJ/TZVP,cc-pVTZ level of theory (number of imaginary frequencies = 0):

Se	-0.07144000	-1.02391400	-0.38289300
N	0.65875000	1.61485500	-0.31738800
C	-0.36655500	0.87413200	-0.52902000
C	1.81350200	0.90471700	-0.01881400
C	3.04424000	1.51033400	0.24563800
H	3.11566200	2.58955500	0.21650800
C	4.14156700	0.71764400	0.53751800
H	5.09876400	1.17943700	0.74266600
C	4.02579100	-0.67691900	0.56906500
H	4.89381700	-1.28213100	0.79798500
C	2.81122000	-1.29658400	0.30952300
H	2.73071000	-2.37545300	0.33541900
C	1.70698500	-0.50254500	0.01503700
C	-1.72429300	1.42497900	-0.85773100
H	-2.11063500	0.95926900	-1.76405900
H	-1.61610500	2.49550400	-1.02477300
N	-2.69958900	1.20380700	0.19918500
C	-3.37060600	0.02176300	0.30161800
H	-2.72412700	1.85430200	0.96698600
O	-3.23145400	-0.87040800	-0.52212700
C	-4.29084300	-0.11709800	1.49337200
H	-5.26796700	-0.44794300	1.14286300
H	-3.89401600	-0.89435400	2.14838900
H	-4.40569300	0.80499600	2.06357300

Cartesian coordinates of the optimized geometry for **3c** (type II) at B2PLYP-D3/TZVP,cc-pVTZ level of theory (number of imaginary frequencies = 0):

Se	-0.07560000	-1.01858400	-0.38137000
N	0.65682500	1.61969600	-0.31707100
C	-0.37078800	0.87083000	-0.52687700
C	1.80847800	0.90411300	-0.02268000
C	3.03651600	1.51019300	0.26260200
H	3.11055300	2.58847100	0.23051300
C	4.13740300	0.71466800	0.53112500
H	5.09148000	1.17462400	0.74997900
C	4.01756100	-0.68128800	0.57111900
H	4.88837800	-1.28739900	0.78232300
C	2.80823600	-1.30051800	0.28903300
H	2.72522900	-2.37839200	0.31885100
C	1.69663000	-0.50190900	0.02315700
C	-1.72483700	1.42806900	-0.85846800

H	-2.11071400	0.97138100	-1.76854000
H	-1.60929200	2.49845400	-1.01557400
N	-2.70320300	1.20906100	0.19456200
C	-3.35500400	0.01629100	0.30336000
H	-2.73220100	1.86004700	0.96113200
O	-3.19538100	-0.88062300	-0.51583000
C	-4.28150800	-0.12568200	1.48798000
H	-5.24951800	-0.47212700	1.13184400
H	-3.87887700	-0.88888400	2.15336100
H	-4.41308800	0.80076300	2.04478100

Cartesian coordinates of the optimized geometry for **3d** (type II) at B3LYP-D3BJ/TZVP,cc-pVTZ level of theory (number of imaginary frequencies = 0):

Te	-0.13406200	-1.05982400	-0.23556200
N	0.70170000	1.73048800	-0.35271000
C	-0.36179600	1.03655200	-0.49837000
C	1.86706800	1.03581600	-0.04198600
C	3.08068600	1.70602800	0.14013100
H	3.10083000	2.78263200	0.03204500
C	4.22336900	0.98866200	0.45137500
H	5.16331100	1.50703700	0.59139000
C	4.17006500	-0.40191000	0.58521000
H	5.06908600	-0.95475300	0.82777000
C	2.97310200	-1.08318500	0.40866300
H	2.94616300	-2.16026400	0.51508700
C	1.81855400	-0.37067100	0.09320200
C	-1.68447600	1.65813200	-0.85093200
H	-2.06006800	1.22618900	-1.77940600
H	-1.52934000	2.72667700	-0.99419600
N	-2.70352300	1.46449900	0.17238400
C	-3.36313100	0.28455400	0.29690400
H	-2.80842300	2.16897300	0.88335300
O	-3.14355800	-0.65653600	-0.45831900
C	-4.37017400	0.19224900	1.41894500
H	-5.31291300	-0.17247200	1.01209200
H	-4.01576800	-0.54381600	2.14227700
H	-4.54005600	1.14067100	1.92877700

Cartesian coordinates of the optimized geometry for **3d** (type II) at B2PLYP-D3/TZVP,cc-pVTZ level of theory (number of imaginary frequencies = 0):

Te	-0.14169700	-1.04721900	-0.24105600
N	0.70564500	1.73814000	-0.34694300
C	-0.36318400	1.04105000	-0.49558300
C	1.86401200	1.03258100	-0.03714300
C	3.08484800	1.69560800	0.13929500
H	3.11045500	2.77236000	0.04043700
C	4.21941600	0.97078500	0.46196800
H	5.16356000	1.48118900	0.59650200
C	4.16070000	-0.42322100	0.57898400
H	5.05437600	-0.97983800	0.82892400
C	2.95879100	-1.09710800	0.41027600
H	2.92554200	-2.17410200	0.50881000
C	1.80849500	-0.37486700	0.08742900
C	-1.68445100	1.66825900	-0.84172400
H	-2.06393800	1.24742900	-1.77274900
H	-1.52678800	2.73751200	-0.97058800
N	-2.69766500	1.46462500	0.18380900
C	-3.34413500	0.27616400	0.30091900
H	-2.80231900	2.16054800	0.90260100
O	-3.11631800	-0.65657000	-0.46723100
C	-4.34396600	0.16343400	1.42559800
H	-5.28347900	-0.20559200	1.01899800

H	-3.97834500	-0.57330100	2.14043200
H	-4.51930200	1.10625800	1.94120500

Cartesian coordinates of the optimized geometry for **3a** (type III) at B3LYP-D3BJ/TZVP,cc-pVTZ level of theory (number of imaginary frequencies = 0):

O	0.38028900	0.73501800	0.74245100
N	0.59043300	-1.42468100	0.13746300
C	-0.19013400	-0.51909600	0.60899800
C	1.80894100	-0.77168100	-0.08195000
C	3.02212500	-1.23209000	-0.58166600
H	3.14385100	-2.26571100	-0.87536800
C	4.05899300	-0.31166900	-0.68519900
H	5.01842500	-0.63296800	-1.06927400
C	3.89347100	1.02737000	-0.30442800
H	4.72670800	1.71121400	-0.40081800
C	2.68242500	1.50074800	0.19598000
H	2.54601300	2.53153700	0.49223700
C	1.66904200	0.56779900	0.29048400
C	-1.60685700	-0.64275800	1.05014600
H	-1.65365600	-0.58208100	2.14276100
H	-1.98412000	-1.61528400	0.74322500
N	-2.45553400	0.36958300	0.44918900
C	-3.54292800	0.04679500	-0.31567000
H	-2.20048700	1.33598200	0.56534100
O	-3.89310000	-1.10370100	-0.50864200
C	-4.28877400	1.22594800	-0.90574400
H	-4.27493200	1.13448000	-1.99223700
H	-5.32924500	1.17627000	-0.58446000
H	-3.87219100	2.19351300	-0.62415100

Cartesian coordinates of the optimized geometry for **3a** (type III) at B2PLYP-D3/TZVP,cc-pVTZ level of theory (number of imaginary frequencies = 0):

O	0.38271100	0.73540200	0.73888400
N	0.59332800	-1.42879600	0.12805800
C	-0.18588400	-0.51703200	0.59871100
C	1.81281600	-0.77411800	-0.07952900
C	3.02135400	-1.23036400	-0.59720300
H	3.14430600	-2.26329100	-0.88997200
C	4.06516200	-0.31529600	-0.67479600
H	5.02044400	-0.63177000	-1.07044600
C	3.89588300	1.02686900	-0.30115200
H	4.73379200	1.70583900	-0.38003700
C	2.69076700	1.49569700	0.21657400
H	2.55279200	2.52593400	0.51088200
C	1.67101700	0.56510700	0.28805800
C	-1.60334500	-0.63687700	1.03534400
H	-1.65614200	-0.56828200	2.12566600
H	-1.98050800	-1.60893200	0.72987400
N	-2.44014100	0.37460200	0.41861500
C	-3.55401400	0.04939200	-0.30696000
H	-2.17130700	1.33911000	0.51108400
O	-3.92951100	-1.10254900	-0.45617200
C	-4.29137300	1.22399500	-0.91244200
H	-4.31181500	1.09758200	-1.99371500
H	-5.32031500	1.20855500	-0.55741000
H	-3.84344800	2.18755900	-0.67395500

Cartesian coordinates of the optimized geometry for **4a** (type I) at B3LYP-D3BJ/TZVP,cc-pVTZ level of theory (number of imaginary frequencies = 0):

N	0.04610000	0.55512800	-0.34439600

C	-0.30319600	-0.68311300	-0.22708400
C	1.43276800	0.56851600	-0.16584700
C	2.35804500	1.60616800	-0.17913100
H	2.04773800	2.62758800	-0.35235600
C	3.69019500	1.27207100	0.03766600
H	4.44040600	2.05203000	0.03385500
C	4.08896300	-0.05317900	0.26110400
H	5.13638600	-0.26934900	0.42587300
C	3.16992300	-1.09994400	0.27732500
H	3.46643500	-2.12509000	0.44966600
C	1.85444300	-0.74352800	0.05823700
C	-1.64860900	-1.30093900	-0.35181100
H	-1.68695200	-2.20243100	0.25794000
H	-1.79111400	-1.62850400	-1.38757400
C	-2.88622500	-0.46479700	0.00949800
O	-3.93571400	-1.03914500	0.25730200
N	-2.75412100	0.87465000	-0.02012200
H	-1.83336900	1.26360100	-0.18712900
C	-3.87843900	1.74178300	0.28179200
H	-3.56828400	2.77506800	0.13793700
H	-4.71965700	1.52307400	-0.37719200
H	-4.21652800	1.60825900	1.31163700
O	0.73745000	-1.54722000	0.02125700

Cartesian coordinates of the optimized geometry for **4a** (type I) at B2PLYP-D3/TZVP,cc-pVTZ level of theory (number of imaginary frequencies = 0):

N	-0.06166000	0.55895600	0.50204500
C	0.30873100	-0.67017200	0.33082300
C	-1.43534400	0.56022300	0.24410600
C	-2.37368000	1.58808700	0.24362400
H	-2.09201800	2.60004400	0.49775300
C	-3.68863300	1.24529600	-0.04983400
H	-4.44781500	2.01521400	-0.05983700
C	-4.04994900	-0.06910200	-0.38427800
H	-5.08414400	-0.29054000	-0.60901600
C	-3.11938800	-1.10507300	-0.38620500
H	-3.38706800	-2.12067900	-0.63846600
C	-1.82050300	-0.73937200	-0.08647500
C	1.65851500	-1.26798200	0.49991300
H	1.68932500	-2.23172300	-0.00186100
H	1.83351000	-1.45398800	1.56352900
C	2.86112900	-0.45992200	-0.00881400
O	3.87684900	-1.04505300	-0.36173400
N	2.74541000	0.88247300	0.02852300
H	1.84627700	1.28146400	0.26425300
C	3.83806800	1.73083400	-0.41120800
H	3.55056100	2.76835400	-0.26174800
H	4.73895000	1.52005900	0.16318600
H	4.06449400	1.56765400	-1.46522300
O	-0.69834900	-1.53279200	-0.02782400

Cartesian coordinates of the optimized geometry for **4b** (type I) at B3LYP-D3BJ/TZVP,cc-pVTZ level of theory (number of imaginary frequencies = 0):

N	-0.00939500	0.47273900	0.64515300
C	0.46290400	-0.71239300	0.44511400
C	-1.35300300	0.56102300	0.31989100
C	-2.13943700	1.70925800	0.42941300
H	-1.69588200	2.62527000	0.79631300
C	-3.47408200	1.64414600	0.06589300
H	-4.09455800	2.52721100	0.14644500
C	-4.03487500	0.44948600	-0.40394700
H	-5.08045300	0.42271800	-0.68225500

C	-3.27024600	-0.70233000	-0.51898800
H	-3.70688000	-1.62300600	-0.88230200
C	-1.92949700	-0.63684200	-0.15363500
C	1.87630200	-1.12245600	0.70322100
H	2.04920600	-2.14715500	0.38265400
H	2.05897100	-1.08508800	1.78193700
C	2.97505600	-0.27114200	0.03781700
O	4.01192400	-0.79598400	-0.33638200
N	2.73375500	1.05252000	-0.03523800
H	1.82283400	1.38555100	0.25397100
C	3.70320500	1.96322800	-0.61584500
H	3.34540600	2.98277500	-0.48474900
H	4.67049900	1.85533600	-0.12390200
H	3.84497000	1.76742500	-1.68112800
S	-0.70487400	-1.89449000	-0.18368400

Cartesian coordinates of the optimized geometry for **4b** (type I) at B2PLYP-D3/TZVP,cc-pVTZ level of theory (number of imaginary frequencies = 0):

N	-0.02206400	0.48260200	0.70404200
C	0.46676000	-0.69850700	0.48724200
C	-1.35720900	0.55539700	0.34314700
C	-2.16780900	1.68643300	0.47612300
H	-1.74548200	2.60114500	0.86837900
C	-3.48601300	1.61647300	0.05834900
H	-4.12395600	2.48449300	0.15331400
C	-4.02113500	0.42044500	-0.44244500
H	-5.05383900	0.39049000	-0.76210200
C	-3.23286300	-0.71205000	-0.58275400
H	-3.64898000	-1.63148600	-0.97039600
C	-1.90845000	-0.64401800	-0.15411500
C	1.88155700	-1.08945500	0.76859600
H	2.05957300	-2.12667900	0.49716500
H	2.06585600	-0.98984800	1.84141500
C	2.95708100	-0.26301800	0.04350300
O	3.97606900	-0.79839400	-0.37244900
N	2.72168300	1.06269700	-0.03479600
H	1.82403500	1.40605000	0.27782500
C	3.67294700	1.95131700	-0.67613300
H	3.31859100	2.97324600	-0.56806800
H	4.65293800	1.85876500	-0.21043400
H	3.77957100	1.71766700	-1.73594800
S	-0.66646200	-1.87824300	-0.18908400

Cartesian coordinates of the optimized geometry for **4c** (type I) at B3LYP-D3BJ/TZVP,cc-pVTZ level of theory (number of imaginary frequencies = 0):

N	0.13054400	0.73601400	0.66431400
C	0.58166700	-0.45785500	0.51239100
C	-1.20694800	0.89240900	0.33396600
C	-1.89333100	2.10516300	0.42897100
H	-1.36668300	2.98278900	0.77979800
C	-3.23136600	2.15724900	0.07564300
H	-3.76873600	3.09393400	0.14735100
C	-3.89780800	1.01118200	-0.37177300
H	-4.94383800	1.07000600	-0.64378200
C	-3.23421200	-0.20388100	-0.47205000
H	-3.75557700	-1.08626200	-0.81858100
C	-1.89067000	-0.25706700	-0.11779600
C	1.98834800	-0.87419600	0.79828700
H	2.14780900	-1.92011200	0.54798500
H	2.17139100	-0.76490300	1.87213900
C	3.09770100	-0.08556100	0.07462900
O	4.11567500	-0.65326700	-0.28837400

N	2.88547600	1.23838100	-0.06119900
H	1.98488300	1.60441900	0.21952300
C	3.86243900	2.09628000	-0.70599500
H	3.54338600	3.13130800	-0.59769500
H	4.84171700	1.97131400	-0.24311100
H	3.96067300	1.86114600	-1.76840000
Se	-0.70316100	-1.73060600	-0.13963700

Cartesian coordinates of the optimized geometry for **4c** (type I) at B2PLYP-D3/TZVP,cc-pVTZ level of theory (number of imaginary frequencies = 0):

N	0.10810000	0.75096200	0.72246900
C	0.58157800	-0.43868600	0.55769700
C	-1.22208700	0.88283400	0.35553600
C	-1.94155200	2.07635300	0.47461400
H	-1.44099200	2.95840500	0.84958700
C	-3.26482900	2.11264700	0.06867200
H	-3.82676100	3.03248200	0.15581800
C	-3.89992600	0.95771200	-0.40679400
H	-4.93454300	1.00494200	-0.71845100
C	-3.20413000	-0.23624600	-0.53319200
H	-3.69979500	-1.12309400	-0.90307400
C	-1.87411600	-0.27524800	-0.11994900
C	1.99119100	-0.82812500	0.86839900
H	2.15902500	-1.88371400	0.67295800
H	2.17555700	-0.65164100	1.93143900
C	3.07463400	-0.06781700	0.08412700
O	4.07667000	-0.64649400	-0.31429000
N	2.86341500	1.25597500	-0.06646600
H	1.97508200	1.63220900	0.23389900
C	3.81891200	2.08808000	-0.77392700
H	3.49757900	3.12386600	-0.69949000
H	4.80908800	1.98558000	-0.33290500
H	3.88683900	1.80732300	-1.82562000
Se	-0.65803200	-1.71741100	-0.14391100

Cartesian coordinates of the optimized geometry for **4d** (type I) at B3LYP-D3BJ/TZVP,cc-pVTZ level of theory (number of imaginary frequencies = 0):

Te	-0.71478900	-1.63429800	-0.12360800
N	0.26607400	0.95761100	0.73177300
C	0.70670500	-0.24013200	0.62239900
C	-1.05275400	1.19552400	0.36906200
C	-1.61782300	2.47114600	0.46256100
H	-1.00693900	3.28544900	0.82965900
C	-2.93712600	2.66990300	0.09231800
H	-3.37254900	3.65803600	0.16592900
C	-3.71084900	1.60368100	-0.37409400
H	-4.74153200	1.76966100	-0.66071700
C	-3.16947100	0.32888800	-0.47329900
H	-3.77788700	-0.48961000	-0.83517100
C	-1.84386200	0.12264500	-0.10383700
C	2.11206300	-0.63326700	0.95514500
H	2.26496100	-1.70286400	0.83913600
H	2.30253900	-0.38199000	2.00344200
C	3.20968700	0.05215200	0.11652300
O	4.18141400	-0.57962700	-0.26596500
N	3.03510700	1.37288700	-0.09665900
H	2.15563100	1.78163100	0.19110700
C	3.98457900	2.15048600	-0.87094900
H	3.80773000	3.20878700	-0.68578100
H	5.00028200	1.89672000	-0.57040900
H	3.89031200	1.95506400	-1.94260900

Cartesian coordinates of the optimized geometry for **4d** (type I) at B2PLYP-D3/TZVP,cc-pVTZ level of theory (number of imaginary frequencies = 0):

Te	-0.62525000	-1.62310700	-0.12989800
N	0.20552600	0.98914300	0.81523400
C	0.69961100	-0.19324600	0.69482100
C	-1.10746700	1.17078300	0.40408400
C	-1.73921700	2.41590000	0.51449900
H	-1.17965700	3.24941500	0.91679600
C	-3.04680600	2.56445700	0.08468500
H	-3.53354600	3.52643400	0.17023900
C	-3.75709300	1.47051400	-0.42325100
H	-4.77929800	1.59870800	-0.75329900
C	-3.15164400	0.22679100	-0.54002400
H	-3.70759500	-0.61103500	-0.93826600
C	-1.83197800	0.07210700	-0.11564800
C	2.11294000	-0.52649400	1.05846500
H	2.29006600	-1.59773100	1.02672500
H	2.30062900	-0.17840100	2.07749500
C	3.16838000	0.11535500	0.14091200
O	4.12571200	-0.52809900	-0.26689400
N	2.97580100	1.42555000	-0.12377000
H	2.11063200	1.84674000	0.18256900
C	3.88358200	2.15748100	-0.98779500
H	3.63068400	3.21377000	-0.94206200
H	4.90981100	2.01816400	-0.65353500
H	3.81443500	1.81556100	-2.02156700

Cartesian coordinates of the optimized geometry for **4c** (type II) at B3LYP-D3BJ/TZVP,cc-pVTZ level of theory (number of imaginary frequencies = 0):

N	0.54954800	1.50184800	-0.41568700
C	-0.38417300	0.62924900	-0.52743100
C	1.78570600	0.96287000	-0.08671700
C	2.93843600	1.73094800	0.09357500
H	2.87922600	2.80444800	-0.02846300
C	4.12785900	1.10231300	0.42269700
H	5.02587600	1.69042600	0.56198400
C	4.18081000	-0.28780400	0.57733300
H	5.11878900	-0.76381100	0.83394800
C	3.04441800	-1.06626200	0.40578400
H	3.09345900	-2.14038900	0.52919600
C	1.84914000	-0.43780900	0.06944100
C	-1.77752000	0.99542400	-0.93756900
H	-1.90166000	0.77407700	-2.00278200
H	-1.88535200	2.07237900	-0.80814400
C	-2.85144900	0.19690800	-0.21098800
O	-2.83332100	-1.02514100	-0.20844100
N	-3.81498600	0.93366200	0.39381100
H	-3.72756900	1.93601800	0.38191900
C	-4.92131700	0.33715700	1.12208300
H	-5.87489900	0.71741000	0.75205300
H	-4.87925500	-0.73804300	0.96673700
H	-4.84456100	0.54257100	2.19206300
Se	0.13438600	-1.19766100	-0.20962100

Cartesian coordinates of the optimized geometry for **4c** (type II) at B2PLYP-D3/TZVP,cc-pVTZ level of theory (number of imaginary frequencies = 0):

N	0.53156600	1.48780200	-0.46375100
C	-0.39284200	0.59648300	-0.57379900
C	1.76325600	0.96071900	-0.10260500
C	2.91172400	1.74076200	0.06733500

H	2.84495500	2.81143700	-0.06863200
C	4.09444200	1.12972900	0.44861100
H	4.98760000	1.72515400	0.58108600
C	4.15882800	-0.26020200	0.61666600
H	5.09147800	-0.72097800	0.91363500
C	3.02761900	-1.04873000	0.46042500
H	3.08451300	-2.12005300	0.59747300
C	1.83837400	-0.43787400	0.06466400
C	-1.78324100	0.93852900	-1.01358000
H	-1.91021100	0.64316000	-2.05851000
H	-1.88703900	2.02110200	-0.95275700
C	-2.84912600	0.19569100	-0.22343600
O	-2.87007300	-1.02848200	-0.17768900
N	-3.77456000	0.97060300	0.39095800
H	-3.63846100	1.96628000	0.40690100
C	-4.82169700	0.38488100	1.21157300
H	-5.58307800	1.13773600	1.39920000
H	-5.26632100	-0.45470500	0.68310900
H	-4.42892600	0.02159500	2.16216900
Se	0.13773500	-1.21210700	-0.21963900

Cartesian coordinates of the optimized geometry for **4d** (type II) at B3LYP-D3BJ/TZVP,cc-pVTZ level of theory (number of imaginary frequencies = 0):

Te	0.06603300	-1.16799600	0.11112500
N	0.59834800	1.69396700	0.28208700
C	-0.39222500	0.88934000	0.35976200
C	1.84778600	1.12637300	0.04702500
C	2.99214200	1.92177900	-0.06704200
H	2.89110200	2.99482700	0.03150500
C	4.22188600	1.32935800	-0.30040900
H	5.10840500	1.94467100	-0.38692500
C	4.32494900	-0.05931500	-0.42501200
H	5.29096000	-0.51412200	-0.60616400
C	3.19747600	-0.86269900	-0.31737800
H	3.29112100	-1.93683500	-0.41766400
C	1.95643500	-0.27671500	-0.07836700
C	-1.77564300	1.37626000	0.67110900
H	-1.87014400	2.40279000	0.31336100
H	-1.89295000	1.40170900	1.76100300
C	-2.88201800	0.47457100	0.14900500
O	-2.78413900	-0.74708500	0.19007200
N	-3.97912100	1.11194200	-0.31966400
H	-3.96761900	2.11802000	-0.34893600
C	-5.15642100	0.41238100	-0.80574600
H	-6.04375500	0.71430100	-0.24645500
H	-5.31726300	0.61061300	-1.86721300
H	-4.99123500	-0.65285800	-0.66620500

Cartesian coordinates of the optimized geometry for **4d** (type II) at B2PLYP-D3/TZVP,cc-pVTZ level of theory (number of imaginary frequencies = 0):

Te	-0.05536700	-1.16226900	-0.12540200
N	-0.59832800	1.69476200	-0.32028000
C	0.39462700	0.88433600	-0.40590700
C	-1.83808300	1.12130900	-0.05372600
C	-2.98668000	1.91362200	0.06412000
H	-2.89173900	2.98577500	-0.04285700
C	-4.20579200	1.31864900	0.34176000
H	-5.09445400	1.92911900	0.43000100
C	-4.30283700	-0.07206000	0.47140400
H	-5.26118500	-0.52670900	0.68566500
C	-3.17328200	-0.87194300	0.36312700
H	-3.25990300	-1.94498400	0.47329200

C	-1.93927700	-0.28160400	0.08392900
C	1.77400200	1.36284700	-0.74940500
H	1.86386300	2.40430800	-0.43940200
H	1.89046300	1.33050100	-1.83754100
C	2.87168000	0.48606300	-0.17552000
O	2.80590200	-0.74097900	-0.22155000
N	3.93439500	1.13270300	0.35057000
H	3.89928700	2.13348900	0.43663500
C	5.05570000	0.40526500	0.92375300
H	5.87365100	1.10099200	1.09150800
H	4.78127300	-0.06576400	1.86792700
H	5.37779800	-0.37207300	0.23483100

Cartesian coordinates of the optimized geometry for **4a** (type III) at B3LYP-D3BJ/TZVP,cc-pVTZ level of theory (number of imaginary frequencies = 0):

N	0.52145600	-1.55516400	-0.26585200
C	-0.32955000	-0.88688500	0.42928300
C	1.68630500	-0.78076800	-0.26564900
C	2.93010900	-0.99117200	-0.85054100
H	3.12751400	-1.88528100	-1.42586800
C	3.89715900	-0.00969200	-0.66298100
H	4.87730200	-0.13873800	-1.10330000
C	3.63524600	1.14671700	0.08435800
H	4.41653900	1.88527200	0.20763900
C	2.39282300	1.36867700	0.67545400
H	2.18290200	2.25590500	1.25665400
C	1.45015000	0.38111400	0.47272600
C	-1.73693100	-1.22611800	0.74023900
H	-1.90850000	-1.14641800	1.81773300
H	-1.91336000	-2.25723100	0.44659600
C	-2.81966800	-0.37724400	0.04720400
O	-3.90408400	-0.86420000	-0.21818700
N	-2.50030300	0.91286200	-0.21021500
H	-1.61716800	1.27230800	0.11361200
C	-3.46543800	1.82422100	-0.80096600
H	-2.94220300	2.71261600	-1.15030500
H	-3.95810500	1.34154800	-1.64365100
H	-4.23445600	2.11858000	-0.08234100
O	0.15161200	0.31975600	0.92830500

Cartesian coordinates of the optimized geometry for **4a** (type III) at B2PLYP-D3/TZVP,cc-pVTZ level of theory (number of imaginary frequencies = 0):

N	0.51582900	-1.55816700	-0.25415900
C	-0.33027200	-0.87760500	0.44085500
C	1.68105900	-0.78471600	-0.25654500
C	2.91647200	-0.99151200	-0.86277200
H	3.11187900	-1.89003000	-1.43041700
C	3.89211000	-0.02064300	-0.66500300
H	4.86536300	-0.14601200	-1.11920500
C	3.63062400	1.14644100	0.06867900
H	4.41790700	1.87643200	0.19727900
C	2.39778800	1.36468400	0.67995800
H	2.18974700	2.25670100	1.25286200
C	1.44643800	0.38505500	0.46720100
C	-1.73709300	-1.21288200	0.76178500
H	-1.90667000	-1.11053900	1.83628800
H	-1.91127100	-2.24853600	0.48598700
C	-2.81072100	-0.37772100	0.04620800
O	-3.88992600	-0.87084800	-0.24229600
N	-2.49320700	0.91439100	-0.20808900
H	-1.62574800	1.28349600	0.14344300
C	-3.46366500	1.81517300	-0.80568800

```
 H                 -2.95374200    2.72735300   -1.10516000
 H                 -3.90795400    1.34708000   -1.68103300
 H                 -4.26531900    2.06119800   -0.10776500
 O                  0.15130300    0.32980300    0.93172000
```

Cartesian coordinates of the optimized geometry for **7a** (type I) at B3LYP-D3BJ/TZVP,cc-pVTZ level of theory (number of imaginary frequencies = 0):

```
 Te                -1.42372200   -1.57941900    0.00041100
 N                 -0.03132400    0.97270100   -0.00263600
 C                  0.25738200   -0.26928300   -0.00239500
 C                 -1.37857700    1.31864700   -0.00104600
 C                 -1.78944800    2.65373500   -0.00113900
 H                 -1.03563500    3.42992200   -0.00246600
 C                 -3.14091000    2.95870300    0.00045000
 H                 -3.45790600    3.99351500    0.00037500
 C                 -4.09665500    1.94016700    0.00214800
 H                 -5.15037600    2.18864300    0.00338200
 C                 -3.70833300    0.60624800    0.00227200
 H                 -4.45763200   -0.17451200    0.00359800
 C                 -2.35281800    0.29527100    0.00068200
 C                  1.66438400   -0.78393900   -0.00429900
 H                  1.81631900   -1.42340700   -0.88048700
 H                  1.81864000   -1.42474500    0.87043300
 N                  2.59912100    0.31502500   -0.00440800
 C                  3.93272000    0.09251000   -0.00042400
 H                  2.22616500    1.25216200   -0.00506200
 O                  4.45657000   -1.00340600    0.00232500
 C                  6.04066600    1.14694700    0.00420300
 H                  6.38054300    0.61613300    0.89338200
 H                  6.38538300    0.61411000   -0.88189800
 H                  6.41703600    2.16643600    0.00407100
 O                  4.61167500    1.26883300    0.00016200
```

Cartesian coordinates of the optimized geometry for **7a** (type I) at B2PLYP-D3/TZVP,cc-pVTZ level of theory (number of imaginary frequencies = 0):

```
 Te                -1.39004200   -1.57506100    0.05888300
 N                 -0.01214500    0.95371100   -0.32824500
 C                  0.26823300   -0.29543100   -0.28894400
 C                 -1.34301000    1.30868100   -0.13806600
 C                 -1.74663600    2.64823000   -0.14675900
 H                 -1.00297300    3.41441300   -0.31795900
 C                 -3.08343200    2.96440900    0.03181900
 H                 -3.39521100    3.99991900    0.02523000
 C                 -4.02763400    1.95713100    0.25724300
 H                 -5.06850700    2.21638600    0.39761600
 C                 -3.64837900    0.62103900    0.26923300
 H                 -4.38709300   -0.15033800    0.43916300
 C                 -2.30520400    0.29765400    0.08350000
 C                  1.65291700   -0.83073100   -0.51221900
 H                  1.65352300   -1.41088000   -1.44127800
 H                  1.92809700   -1.51903100    0.28846400
 N                  2.61355900    0.24672400   -0.56550500
 C                  3.85496600    0.08389300   -0.03975400
 H                  2.24908100    1.18161000   -0.66884900
 O                  4.32781700   -0.97045700    0.33738000
 C                  5.85201100    1.20339800    0.49022900
 H                  5.86049300    0.84162100    1.51627000
 H                  6.45935100    0.54398300   -0.12635200
 H                  6.22748500    2.22033600    0.44291500
 O                  4.51031400    1.27201500   -0.01220400
```

Cartesian coordinates of the optimized geometry for **7a** (type II) at B3LYP-D3BJ/TZVP,cc-pVTZ level of theory (number of imaginary frequencies = 0):

Te	0.08738000	-0.98375700	-0.38725900
N	1.06360700	1.75888400	-0.24219100
C	-0.02395400	1.13965400	-0.49824900
C	2.17290500	0.98170400	0.07642700
C	3.40588000	1.56946800	0.37507300
H	3.48612500	2.64838100	0.35152600
C	4.49097700	0.76986500	0.69211000
H	5.44589900	1.22460700	0.92245900
C	4.36030900	-0.62170200	0.71610300
H	5.21470200	-1.23900200	0.96427000
C	3.14307500	-1.22230400	0.42325700
H	3.05582600	-2.30112700	0.44548400
C	2.04673800	-0.42618900	0.10104200
C	-1.29401400	1.85883600	-0.86449000
H	-1.63758900	1.52870300	-1.84531200
H	-1.07520900	2.92482000	-0.91189200
N	-2.37649300	1.64861700	0.08293700
C	-3.08433400	0.49269700	0.07964900
H	-2.43636100	2.23363200	0.90050000
O	-2.94731700	-0.40064300	-0.73709800
O	-3.96893300	0.46423800	1.09998600
C	-4.75405000	-0.73510600	1.20058400
H	-4.11066200	-1.60292400	1.34187200
H	-5.39182100	-0.58893100	2.06778700
H	-5.35567900	-0.87558200	0.30339800

Cartesian coordinates of the optimized geometry for **7a** (type II) at B2PLYP-D3/TZVP,cc-pVTZ level of theory (number of imaginary frequencies = 0):

Te	0.07263700	-0.96526900	-0.40244100
N	1.06612300	1.76744800	-0.22913500
C	-0.02769100	1.15008600	-0.49521600
C	2.16515600	0.97534400	0.08579400
C	3.40597000	1.55119300	0.38519100
H	3.49398700	2.62910100	0.37676000
C	4.47919000	0.73920300	0.71029500
H	5.43874100	1.18249200	0.93983800
C	4.34021800	-0.65391000	0.70972900
H	5.18656700	-1.27872800	0.96302800
C	3.11727600	-1.24284600	0.41839100
H	3.02130500	-2.32046200	0.42662100
C	2.02889300	-0.43205900	0.09215700
C	-1.29447700	1.87887300	-0.85216400
H	-1.64407200	1.56209700	-1.83410100
H	-1.07031800	2.94351900	-0.88466700
N	-2.37317600	1.66197100	0.09696700
C	-3.06224700	0.49310300	0.08975700
H	-2.40294500	2.21332100	0.93888900
O	-2.92993400	-0.38424000	-0.74791000
O	-3.92287900	0.43442100	1.12832400
C	-4.68516000	-0.78109100	1.21306400
H	-4.02435300	-1.63836700	1.32045700
H	-5.30648400	-0.66596400	2.09469000
H	-5.30044800	-0.90777400	0.32532300

6. Crystal Structure Data

Crystal Structure Data of 7b

Table S8. Crystal structure data and structure refinement for **7b**.

Empirical formula	$C_{23} H_{18} N_2 O_2 Te$
Formula weight	481.99
Density (calculated)	1.657 g·cm^{-3}
F(000)	3808
Temperature	133(2) K
Crystal size	0.342 × 0.101 × 0.062 mm
Crystal color	colorless
Crystal description	needle
Wavelength	1.54178 Å
Crystal system	monoclinic
Space group	*Cc*
Unit cell dimensions	
a [Å]	38.4257(16)
b [Å]	9.9457(4)
c [Å]	22.2349(9)
α [°]	90
β [°]	114.6000(10)
γ [°]	90
Volume	7726.3(5) Å^3
Z	16
Cell measurement reflections used	9994
Cell measurement θ min/max	4.04°/78.78°
Diffractometer control software	Bruker APEX3(v2017.3-0)
Diffractometer measurement device	Bruker D8 Venture (Photon II detector)
Diffractometer measurement method	Data collection strategy APEX 3/Queen
θ range for data collection	2.529°- 79.201°
Completeness to θ = 67.679°	99.9%
Completeness to θ_{max} = 79.201°	99.0%
Index ranges	$-48 \leq h \leq 48$
	$-12 \leq k \leq 11$
	$-28 \leq l \leq 28$
Computing data reduction	Bruker APEX3(v2017.3-0)
Absorption coefficient	12.329 mm^{-1}
Absorption correction	Semi-empirical from equivalents
Computation absorption correction	SADABS
Max./min. Transmission	0.75/0.31
R_{merg} before/after correction	0.1784/0.0820
Computing structure solution	Bruker APEX3(v2017.3-0)
Computing structure refinement	SHELXL-2017/1 (Sheldrick, 2017)
Refinement method	Full-matrix least-squares on F^2
Reflections collected	87338
Independent reflections	15733
R_{int}	0.0474
Reflections with $I > 2\sigma(I)$	14553
Restraints	3340
Parameter	1178

GooF	1.170
Weighting details	$w = 1/[\sigma^2(F_{obs}^2) + 0.0478aP)^2 + 51.1119P]$<br>where $P = (F_{obs}^2 + 2F_{calc}^2)/3$
R_1 [$I > 2\sigma(I)$]	0.0553
wR_2 [$I > 2\sigma(I)$]	0.1415
R_1 [all data]	0.0592
wR_2 [all data]	0.1449
Absolute structure parameter	0.468(15)
Largest diff. peak and hole	1.195/-1.294

Comments

Treatment of hydrogen atoms

Riding model on idealized geometries with the 1.2 fold isotropic displacement parameters of the equivalent *Uij* of the corresponding carbon atom.

Twinning

Refined as a 2-component inversion twin.

Disorder

The Te bicyclic residues are disordered over two position. The residues were realted by non-crystallographic symmetry restraints. The corresponding bond lengths of the disordered parts were restrained to be equal (SADI) and to lie on a mutual plane (FLAT). RIGU restraints were applied to all adp and the ISOR restraints to those of the disordered light atoms. The adp of the disordered light atoms were additionally restrained to be similar (SIMU). To the adp of C2_2 an additional more strict ISOR restraint (sigma= 0.01) was applied to avoid a non positive definite. Due to the vast use of restraints quantitative results may be of limited validity.

Absolute structure

Considering the apparent pseudo-symmetry and the flack parameter of approx. 0.5 *C*2/*c* was tried. No model with and R1 of better than 40% could be obtained. The strongest scatteres can be related either by a 2_1 screw-axis or a series of inversion centers (origin shift of 0.25 0 0.25). In both cases this results in a double image of the light atoms which in neither orientation could be refined satisfactorily. Refinement as disorder over two positions also failed thus despite apparent centro-symmetry the non-centrosymmetric model was chosen.

Crystal Structure Data of 16

Table S9. Crystal structure data and structure refinement for **16**.

Empirical formula	$C_{44} H_{59} N_{10} O_9 Te_2$
Formula weight	1127.21
Density (calculated)	1.358 g·cm^{-3}
F(000)	2276
Temperature	100(2) K
Crystal size	0.331 × 0.114 × 0.018 mm
Crystal color	colorless
Crystal description	plate
Wavelength	1.54178 Å
Crystal system	orthorhombic
Space group	$P2_12_12$
Unit cell dimensions	
a [Å]	17.854(2)
b [Å]	27.107(4)
c [Å]	11.3909(14)
α [°]	90
β [°]	90
γ [°]	90
Volume	5513.0(12) Å^3
Z	4
Cell measurement reflections used	9937
Cell measurement θ min/max	2.96°/78.57°
Diffractometer control software	Bruker APEX3(v2017.3-0)
Diffractometer measurement device	Bruker D8 Venture (Photon II detector)
Diffractometer measurement method	Data collection strategy APEX 3/Queen
θ range for data collection	2.964°- 80.615°
Completeness to θ = 67.679°	100.0%
Completeness to θ_{max} = 80.615°	99.4%
Index ranges	$-22 \leq h \leq 22$
	$-34 \leq k \leq 34$
	$-14 \leq l \leq 11$
Computing data reduction	Bruker APEX3(v2017.3-0)
Absorption coefficient	8.813 mm^{-1}
Absorption correction	Semi-empirical from equivalents
Computation absorption correction	SADABS
Max./min. Transmission	0.75/0.50
R_{merg} before/after correction	0.1596/0.1170
Computing structure solution	Bruker APEX3(v2017.3-0)
Computing structure refinement	SHELXL-2017/1 (Sheldrick, 2017)
Refinement method	Full-matrix least-squares on F^2
Reflections collected	89442
Independent reflections	11950
R_{int}	0.1162
Reflections with $I > 2\sigma(I)$	10533
Restraints	0
Parameter	606
GooF	1.129
Weighting details	$w = 1/[\sigma^2(F_{obs}^2) + (0.0815P)^2 + 27.9161P]$
	where $P = (F_{obs}^2 + 2F_{calc}^2)/3$

R_1 [$I > 2\sigma(I)$]	0.0859
wR_2 [$I > 2\sigma(I)$]	0.2157
R_1 [all data]	0.0943
wR_2 [all data]	0.2209
Absolute structure parameter	0.157(4)
Largest diff. peak and hole	2.146/-1.621

Comments

Treatment of hydrogen atoms

Riding model on idealized geometries with the 1.2 fold isotropic displacement parameters of the equivalent *Uij* of the corresponding carbon atom. The methyl groups are idealized with tetrahedral angles in a combined rotating and rigid group refinement with the 1.5 fold isotropic displacement parameters of the equivalent *Uij* of the corresponding carbon atom. The hydrogen atoms of the water molecules could not be identified but were included in the sum formula.

Disorder

One of the water molecules is disordered over two position.

SQUEEZE

The structure contains highly disordered solvent - possibly EtOAc or methanol. The final refinement was done with a solvent free dataset from a PLATON/SQUEEZE run. For details see: A. L. Spek, *Acta Cryst. A* **1990**, *46*, 194-201. Since the nature and amount of the solvent is not clear it was not included in the sum formula.

Possible twinning by inversion

The starting material was enantiopure and a racemization of all four stereo centers is highly unlikely thus the possible twinning by inversion suggested by the Flack-parameter was ignored. The deviation from zero likely results from the low data quality.

Weak diffraction data

The intensities at resolutions > 1 Å were very weak and a high percentage < $2\sigma(I)$. Consequently, the model should be carefully interpreted and any conclusions draw from it supported by other means. Quantitative results may be unreliable.

Crystal Structure Data of 17

Table S10. Crystal structure data and structure refinement for **17**.

Empirical formula	$C_{65.04}$ $H_{82.07}$ $Cl_{1.31}$ N_{12} $O_{6.35}$ Te_3
Formula weight	1562.66
Density (calculated)	1.539 g·cm^{-3}
F(000)	1571
Temperature	100(2) K
Crystal size	0.286 × 0.253 × 0.224 mm
Crystal color	colorless
Crystal description	block
Wavelength	0.71073 Å
Crystal system	hexagonal
Space group	$P6_3$
Unit cell dimensions	
a [Å]	17.7926(5)
b [Å]	17.7926(5)
c [Å]	12.2997(4)
α [°]	90
β [°]	90
γ [°]	120
Volume	3372.1(2) Å^3
Z	2
Cell measurement reflections used	9789
Cell measurement *θ* min/max	2.83°/37.69°
Diffractometer control software	BRUKER APEX2(v2009.5-1)
Diffractometer measurement device	Bruker D8 KAPPA II (APEX II detector)
Diffractometer measurement method	Data collection strategy APEX 2/COSMO
θ range for data collection	2.119°- 40.349°
Completeness to *θ* = 25.242°	100.0%
Completeness to θ_{max} = 40.349°	98.7%
Index ranges	$-26 \leq h \leq 30$
	$-28 \leq k \leq 32$
	$-20 \leq l \leq 22$
Computing data reduction	BRUKER APEX2(v2009.5-1)
Absorption coefficient	1.400 mm^{-1}
Absorption correction	Semi-empirical from equivalents
Computation absorption correction	SADABS
Max./min. Transmission	0.75/0.63
R_{merg} before/after correction	0.0656/0.0482
Computing structure solution	BRUKER APEX2(v2009.5-1)
Computing structure refinement	SHELXL-2017/1 (Sheldrick, 2017)
Refinement method	Full-matrix least-squares on F^2
Reflections collected	180707
Independent reflections	13380
R_{int}	0.0282
Reflections with $I > 2\sigma(I)$	10644
Restraints	104
Parameter	333
GooF	1.163
Weighting details	$w = 1/[\sigma^2(F_{obs}^2) + (0.0530P)^2 + 2.2877P]$
	where $P = (F_{obs}^2 + 2F_{calc}^2)/3$

R_1 [$I > 2\sigma(I)$]	0.0388
wR_2 [$I > 2\sigma(I)$]	0.0958
R_1 [all data]	0.0617
wR_2 [all data]	0.1152
Absolute structure parameter	-0.036(3)
Largest diff. peak and hole	2.554/-0.832

Comments

Treatment of hydrogen atoms

Riding model on idealized geometries with the 1.2 fold isotropic displacement parameters of the equivalent *Uij* of the corresponding carbon atom. The methyl groups are idealized with tetrahedral angles in a combined rotating and rigid group refinement with the 1.5 fold isotropic displacement parameters of the equivalent *Uij* of the corresponding carbon atom. The NH hydrogen was refined freely with its NH bond lengths restrained to be equal to 0.85 Å (DFIX).

Disordered solvent

The structure contains a THF molecule disordered over a three-fold rotational axis. A second one is partly occupied and sharing its location with a dichloromethane molecule. All corresponding 1,2 and 1,3 distances of the THF were restrained to be equal (SADI). The C-O bond lengths were restrained to be equal to 1.43 Å (DFIX). The bond angle of the dichloromethane was restrained to the tetrahedral angle and its bond lengths to be equal (SADI, DFIX). RIGU restraints were applied to all solvents adp. All solvent molecules are highly disordered and amount and nature of the solvent should not be taken for granted.

Absolute structure

The absolute structure could be determined reliably. Parsons quotient method was used to determine the absolute structure parameter *x*. For more details see S. Parsons, H. D. Flack, *Acta Cryst. A* **2004**, *60*, 61 and S. Parsons, H. D. Flack, T. Wagner, *Acta Cryst.* B **2013**, 69, 249-259.

7. ^{1}H NMR and ^{13}C NMR Spectra of New Compounds

The field strengths and solvents of the following spectra are given in the Experimental Section.

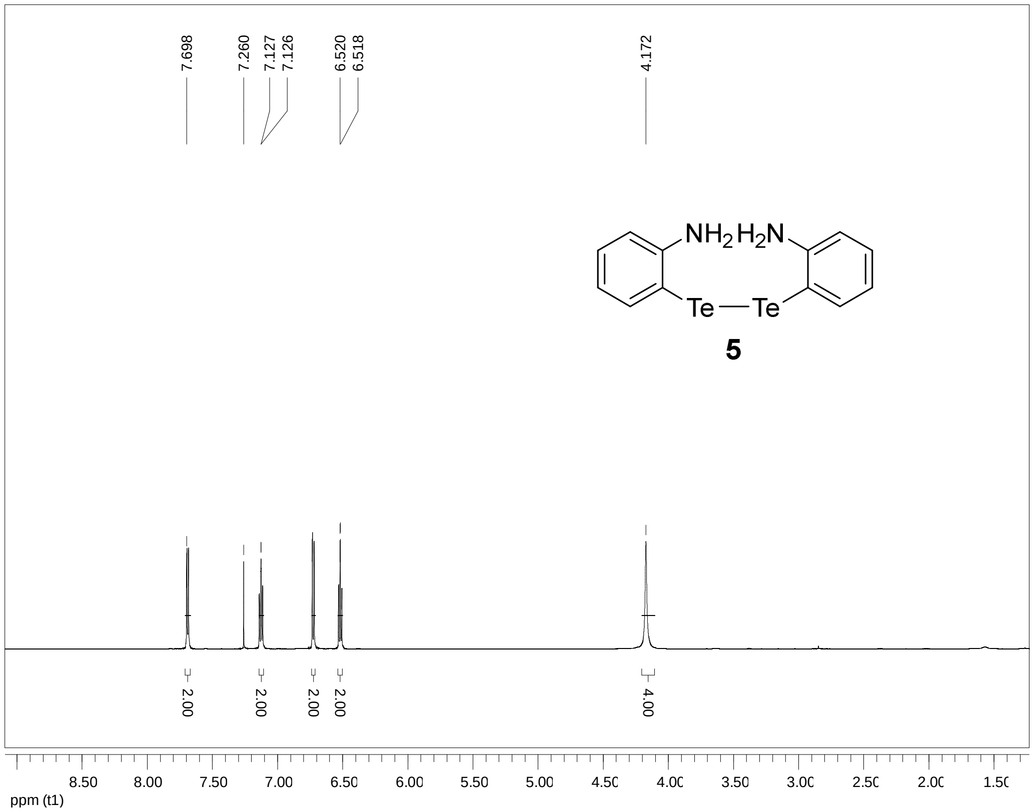

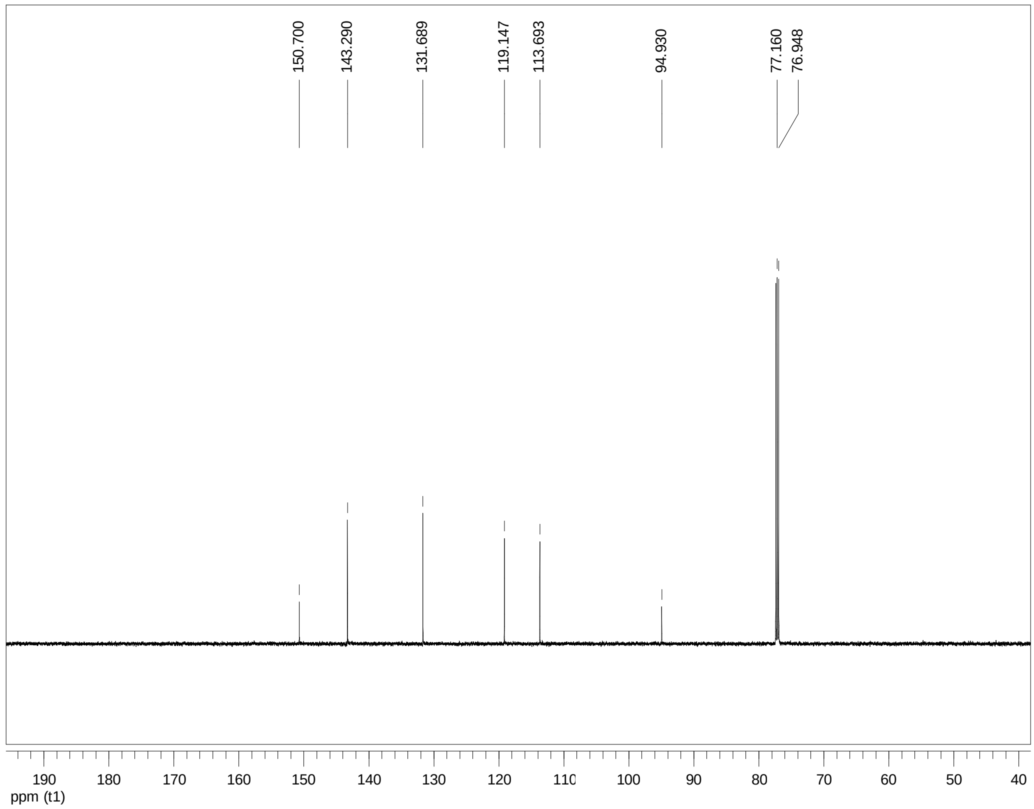

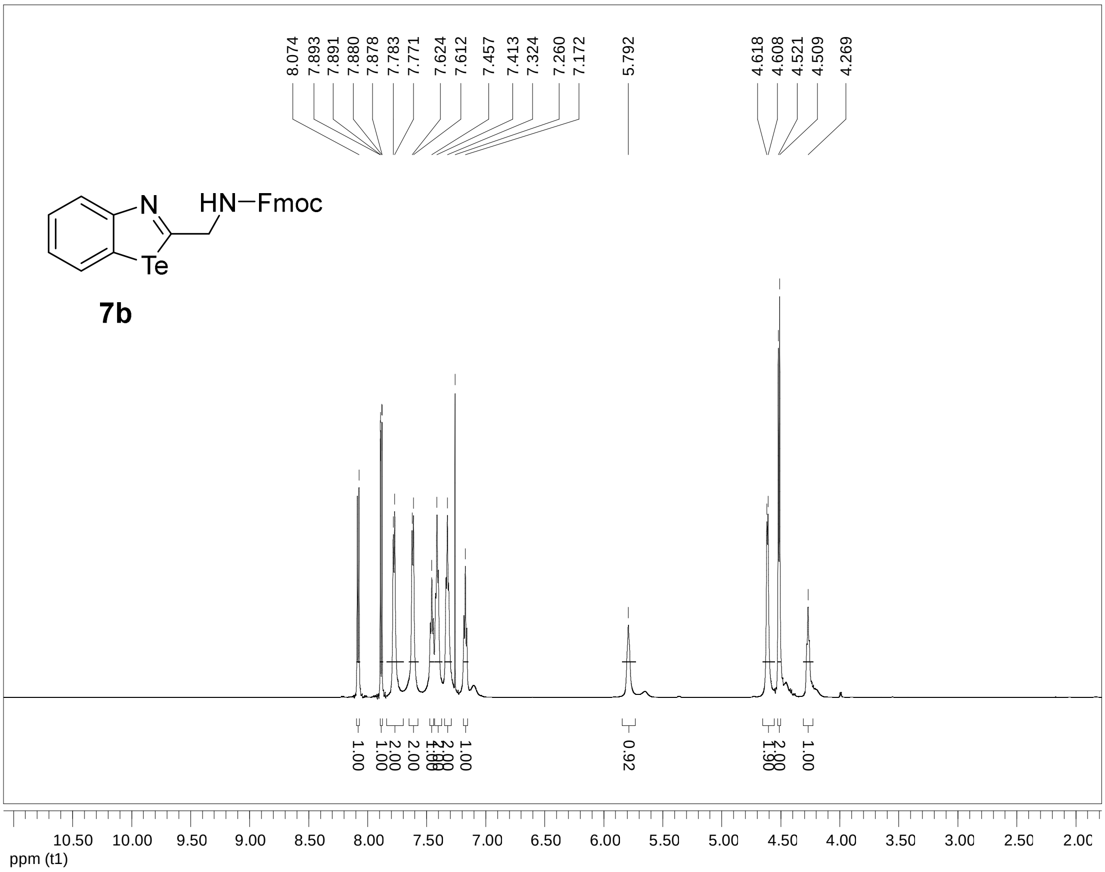
8.074
7.893
7.891
7.880
7.878
7.783
7.771
7.624
7.612
7.457
7.413
7.324
7.260
7.172
5.792
4.618
4.608
4.521
4.509
4.269
N
HN−Fmoc
Te
7b
1.00
1.00
2.00
2.00
2.00
1.00
0.92
1.00
1.00
10.50 10.00 9.50 9.00 8.50 8.00 7.50 7.00 6.50 6.00 5.50 5.00 4.50 4.00 3.50 3.00 2.50 2.00
ppm (t1)

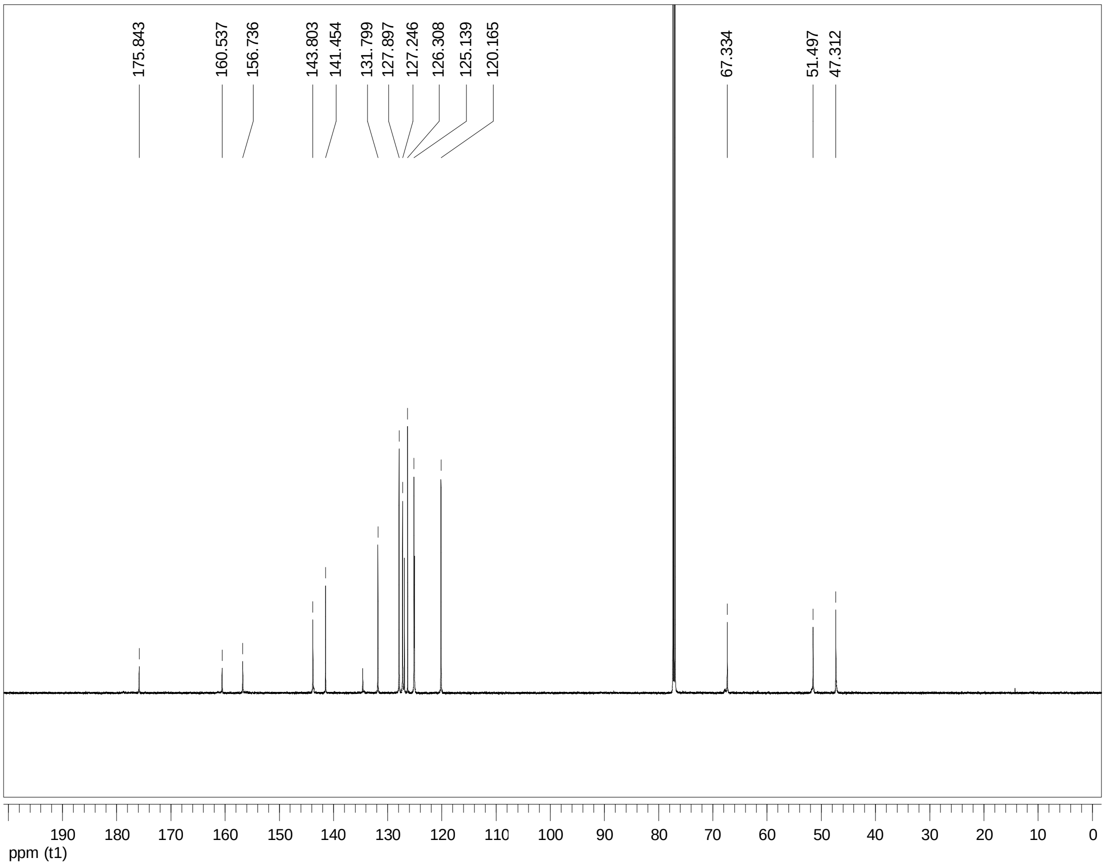
175.843
160.537
156.736
143.803
141.454
131.799
127.897
127.246
126.308
125.139
120.165
67.334
51.497
47.312
190 180 170 160 150 140 130 120 110 100 90 80 70 60 50 40 30 20 10 0
ppm (t1)

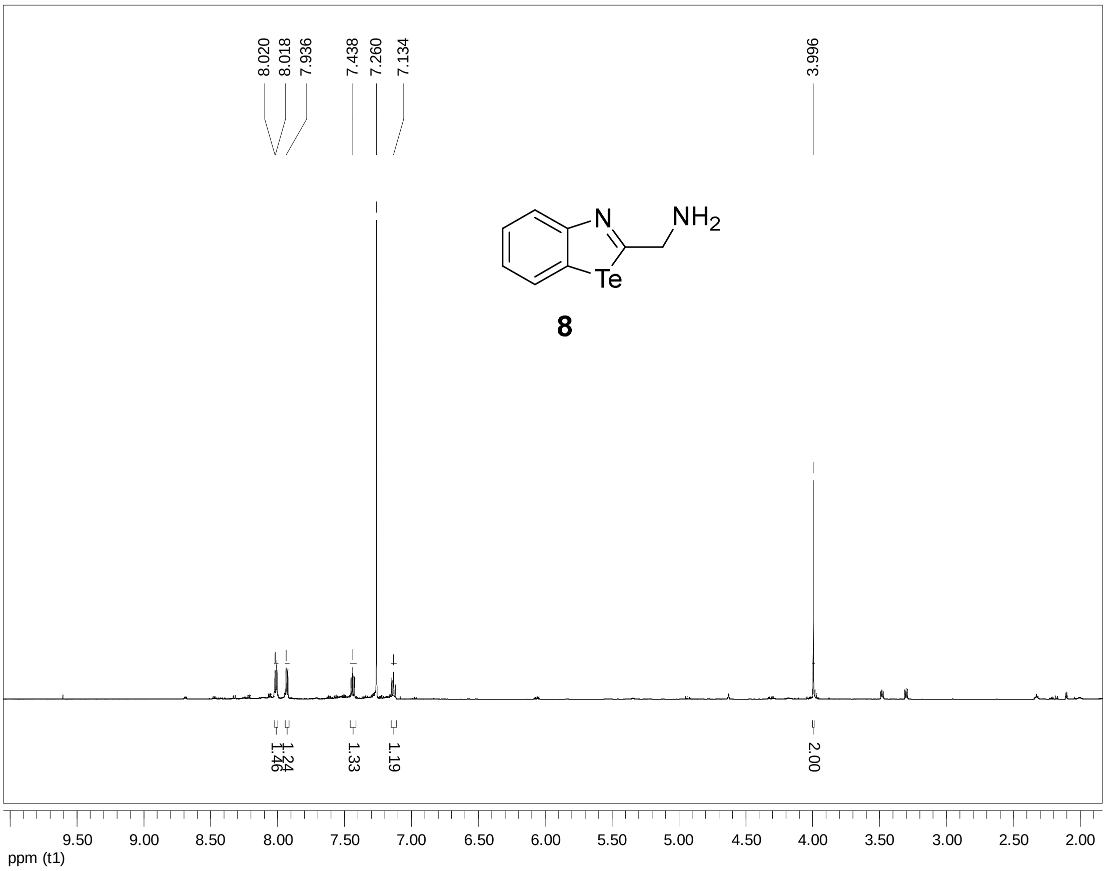
8.020
8.018
7.936
7.438
7.260
7.134
3.996
N
NH2
Te
8
1.46
1.24
1.33
1.19
2.00
9.50
9.00
8.50
8.00
7.50
7.00
6.50
6.00
5.50
5.00
4.50
4.00
3.50
3.00
2.50
2.00
ppm (t1)

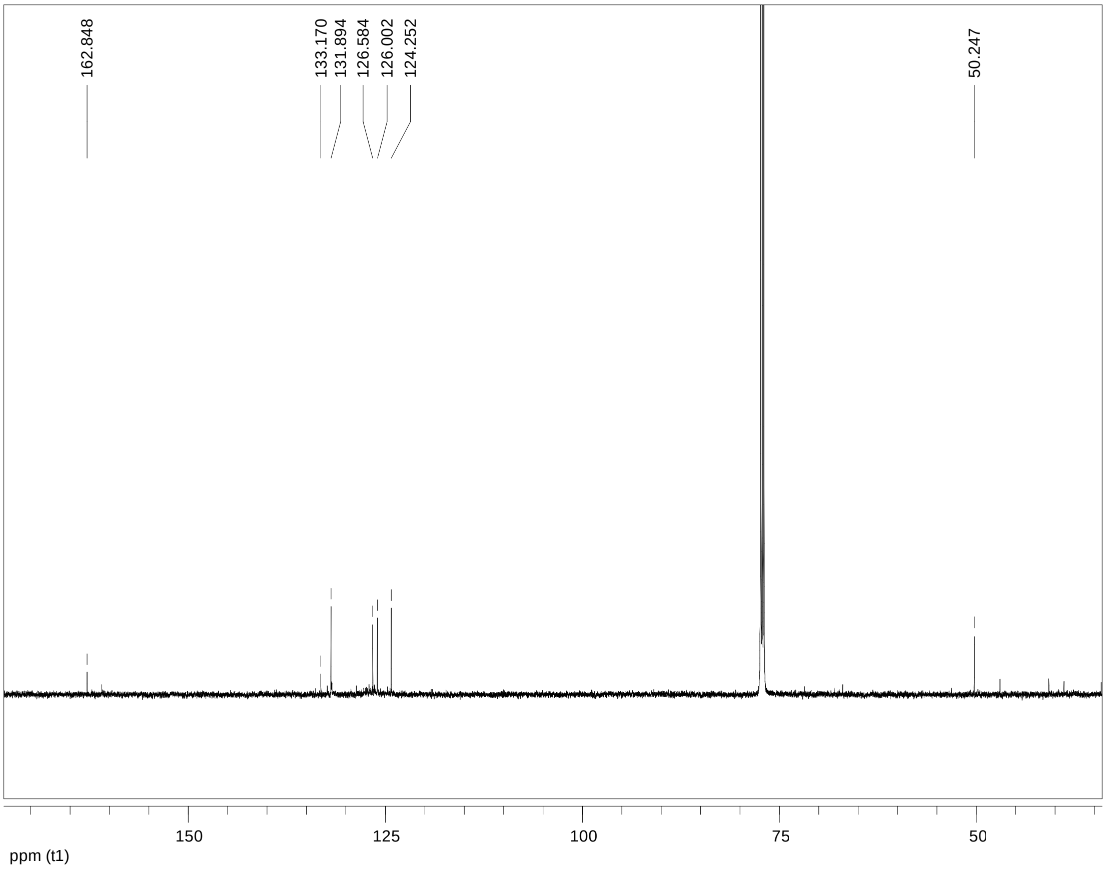
162.848
133.170
131.894
126.584
126.002
124.252
50.247
150
125
100
75
50
ppm (t1)

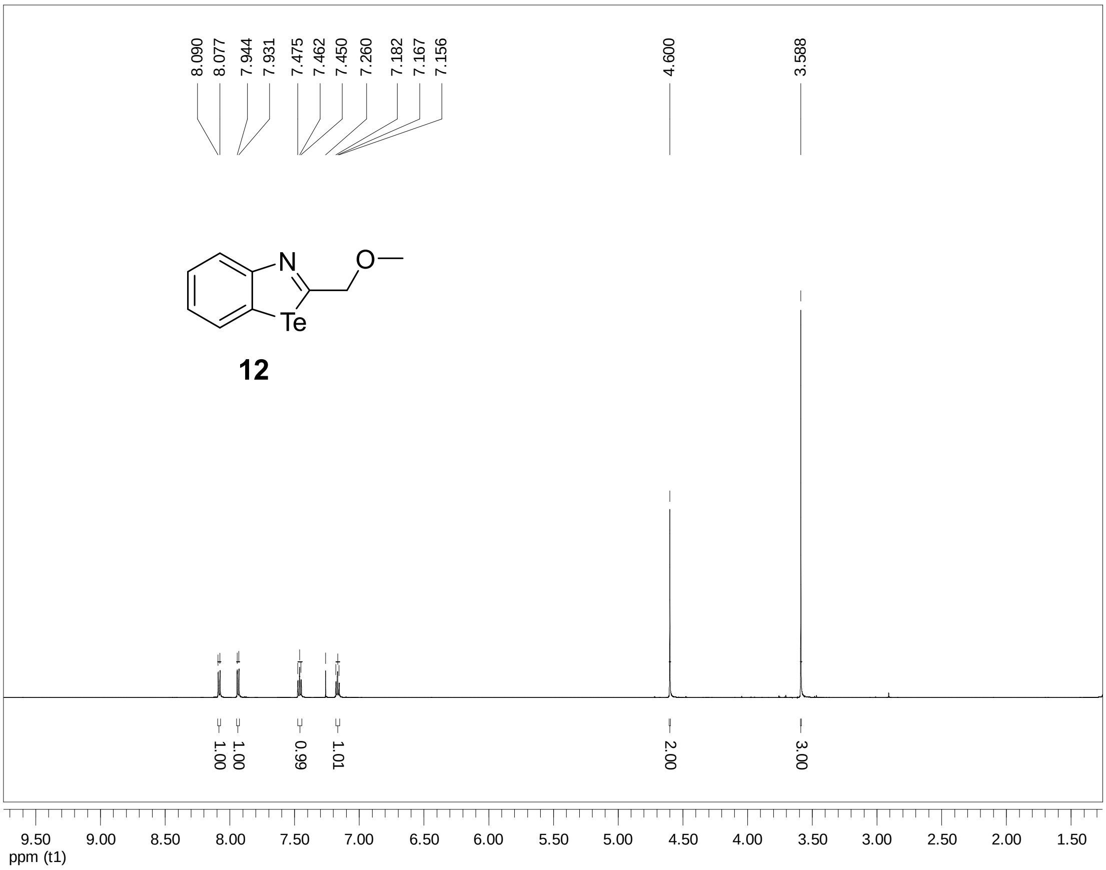
8.090
8.077
7.944
7.931
7.475
7.462
7.450
7.260
7.182
7.167
7.156
4.600
3.588
N
O
Te
12
1.00
1.00
0.99
1.01
2.00
3.00
9.50
9.00
8.50
8.00
7.50
7.00
6.50
6.00
5.50
5.00
4.50
4.00
3.50
3.00
2.50
2.00
1.50
ppm (t1)

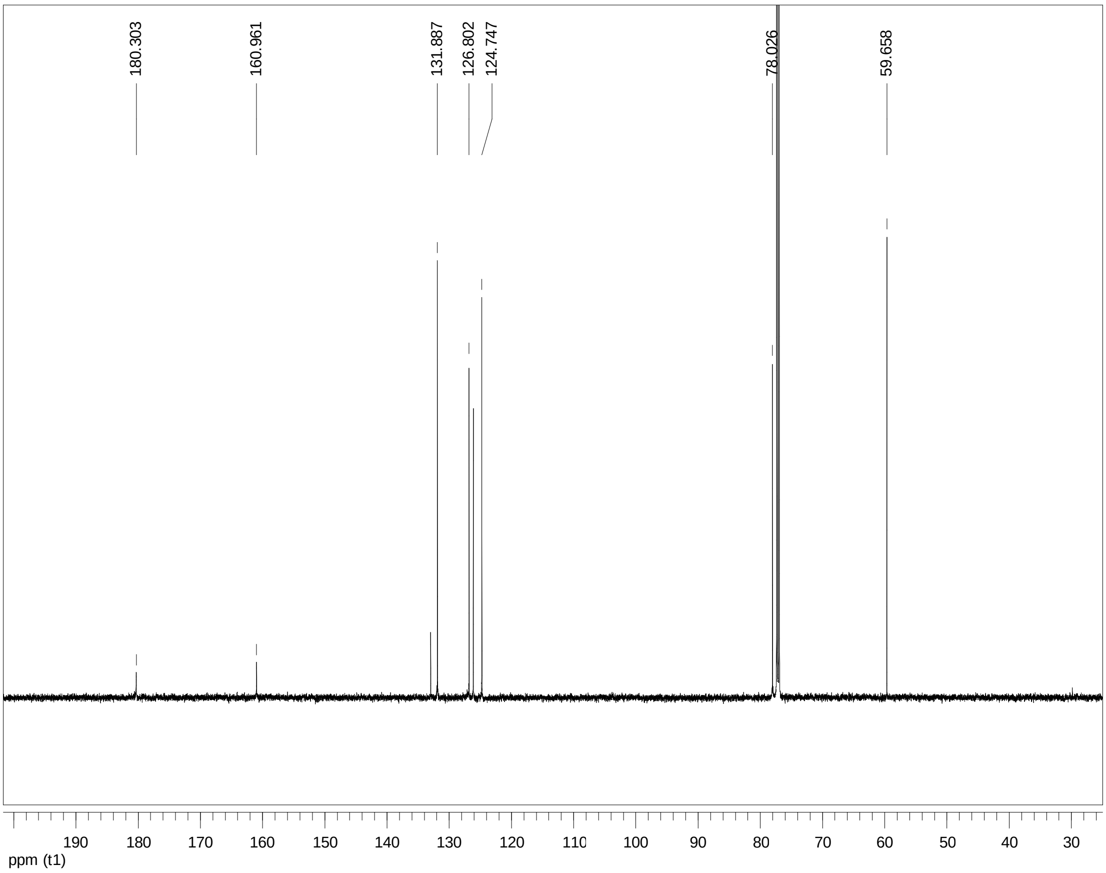
180.303
160.961
131.887
126.802
124.747
78.026
59.658
190
180
170
160
150
140
130
120
110
100
90
80
70
60
50
40
30
ppm (t1)

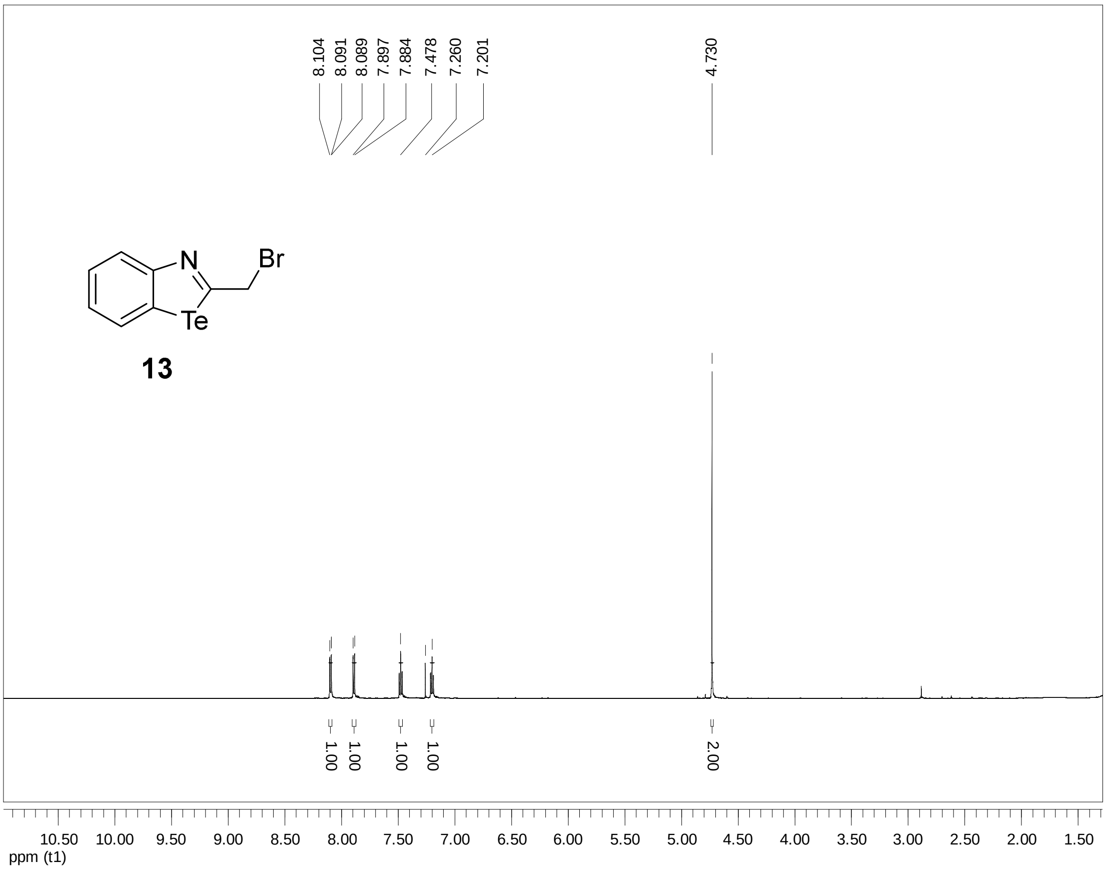
8.104
8.091
8.089
7.897
7.884
7.478
7.260
7.201
4.730
N
Br
Te
1.00
1.00
1.00
1.00
2.00
10.50 10.00 9.50 9.00 8.50 8.00 7.50 7.00 6.50 6.00 5.50 5.00 4.50 4.00 3.50 3.00 2.50 2.00 1.50
ppm (t1)

13

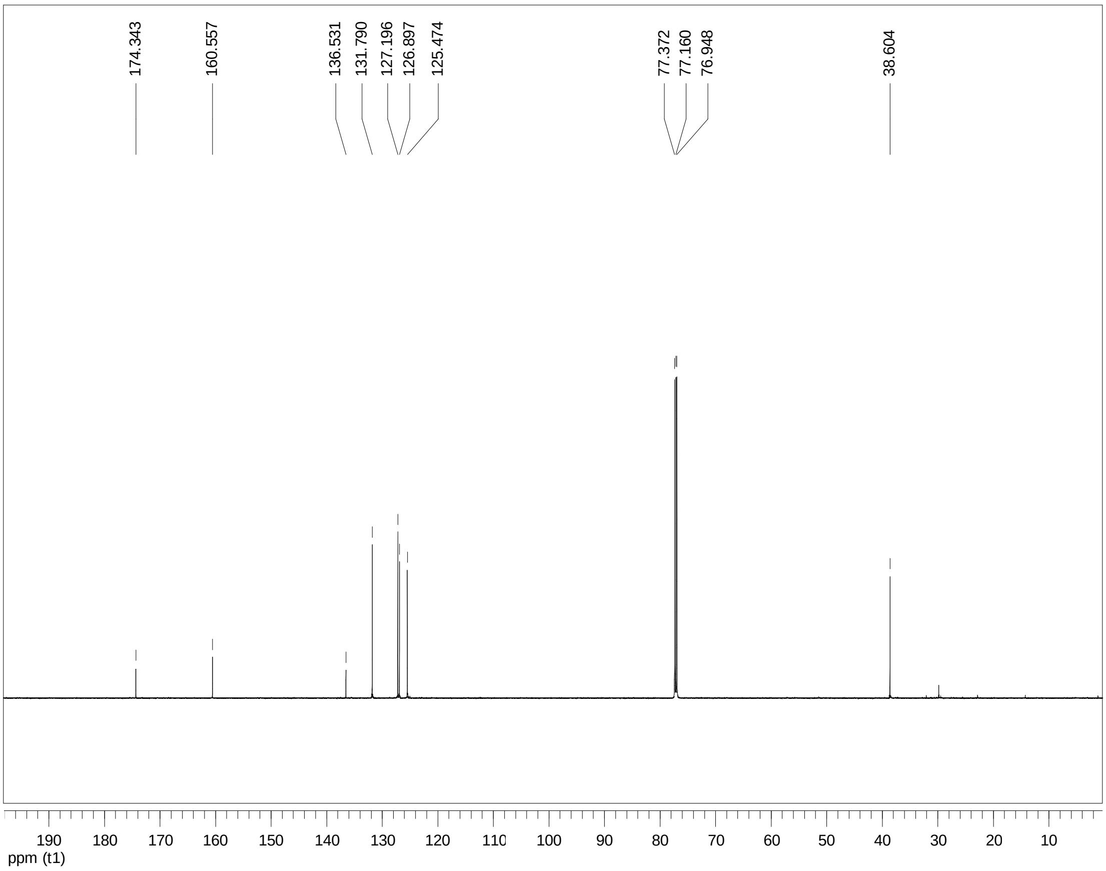
174.343
160.557
136.531
131.790
127.196
126.897
125.474
77.372
77.160
76.948
38.604
190 180 170 160 150 140 130 120 110 100 90 80 70 60 50 40 30 20 10
ppm (t1)

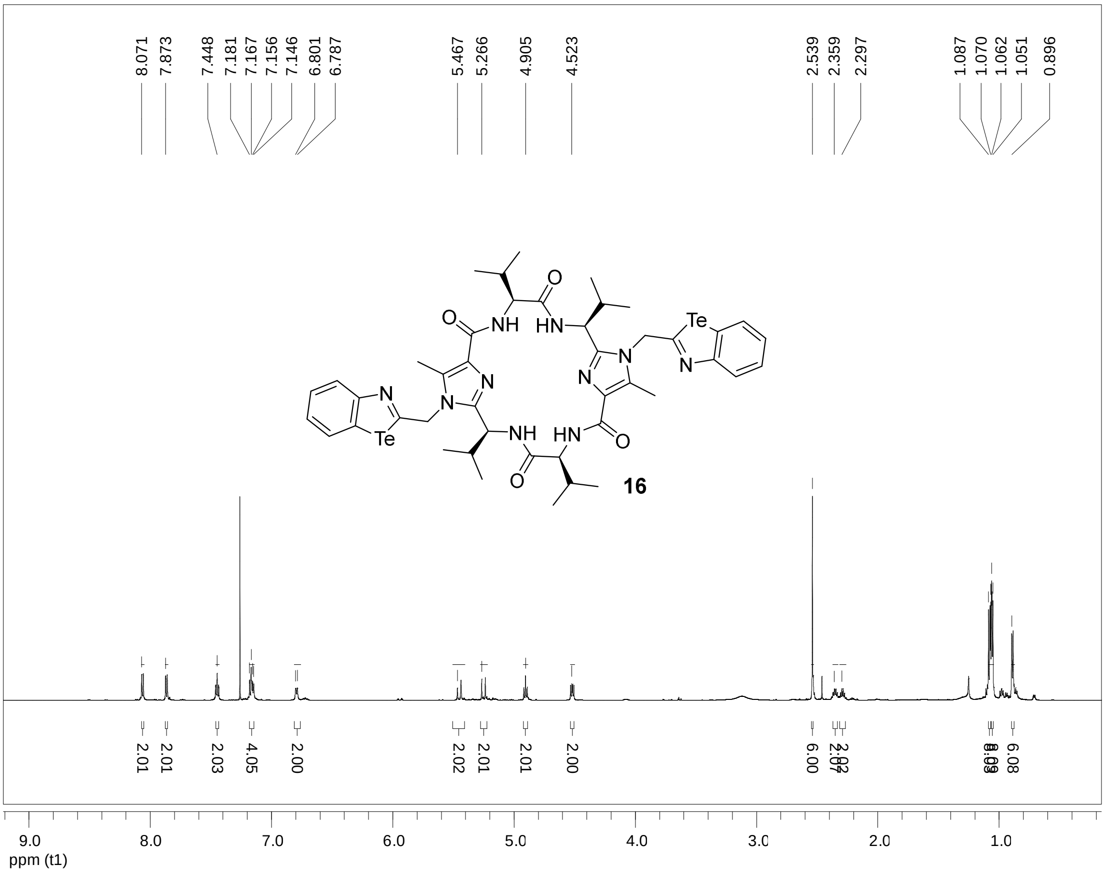
8.071
7.873
7.448
7.181
7.167
7.156
7.146
6.801
6.787
5.467
5.266
4.905
4.523
2.539
2.359
2.297
1.087
1.070
1.062
1.051
0.896
16
2.01
2.01
2.03
4.05
2.00
2.02
2.01
2.01
2.00
6.00
6.08
9.0
8.0
7.0
6.0
5.0
4.0
3.0
2.0
1.0
ppm (t1)

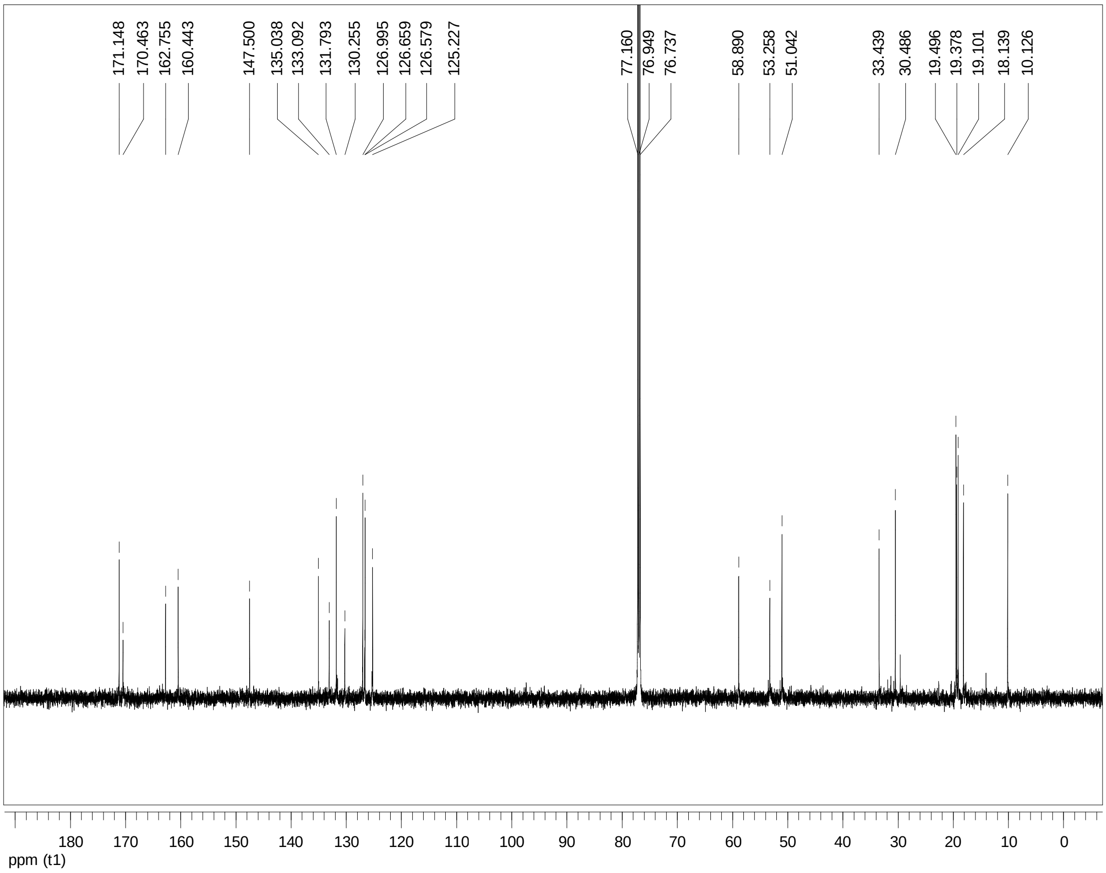
171.148
170.463
162.755
160.443
147.500
135.038
133.092
131.793
130.255
126.995
126.659
126.579
125.227
77.160
76.949
76.737
58.890
53.258
51.042
33.439
30.486
19.496
19.378
19.101
18.139
10.126
180
170
160
150
140
130
120
110
100
90
80
70
60
50
40
30
20
10
0
ppm (t1)

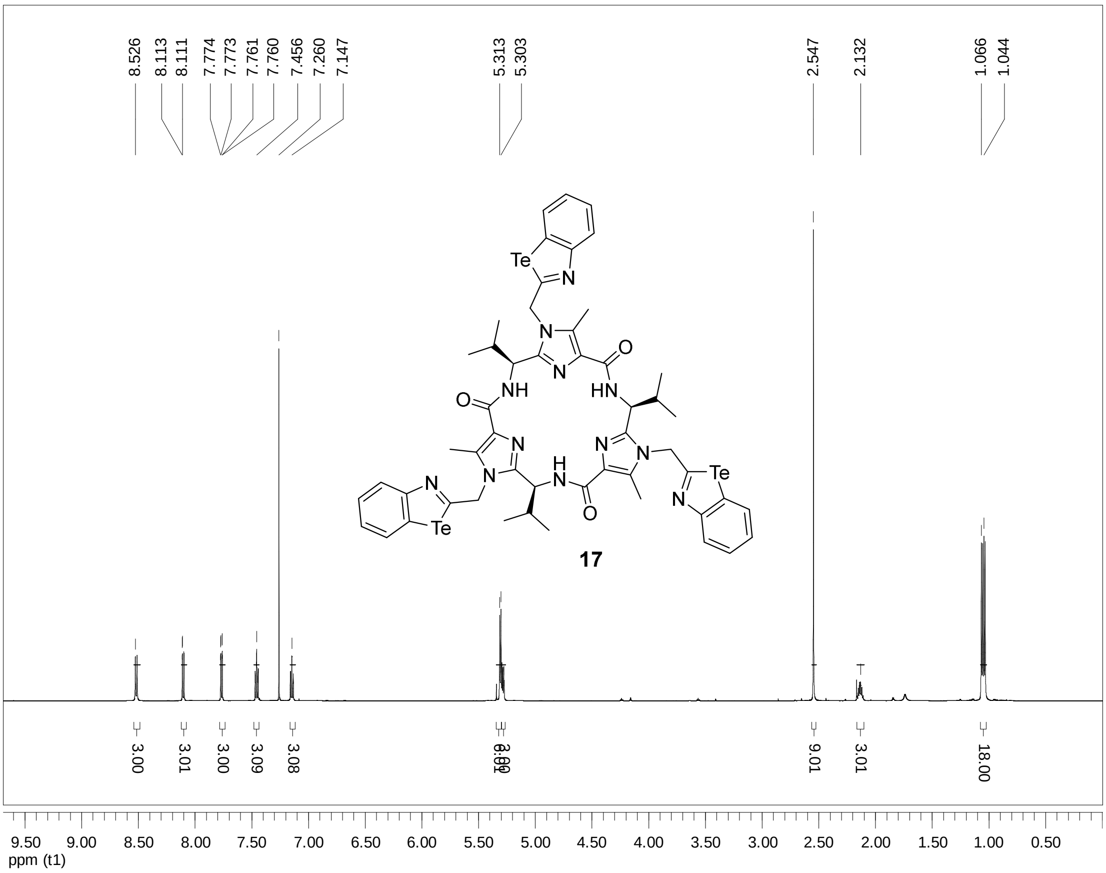
8.526
8.113
8.111
7.774
7.773
7.761
7.760
7.456
7.260
7.147
5.313
5.303
2.547
2.132
1.066
1.044
Te
N
O
NH
HN
H
17
3.00
3.01
3.00
3.09
3.08
9.01
3.01
18.00
9.50 9.00 8.50 8.00 7.50 7.00 6.50 6.00 5.50 5.00 4.50 4.00 3.50 3.00 2.50 2.00 1.50 1.00 0.50
ppm (t1)

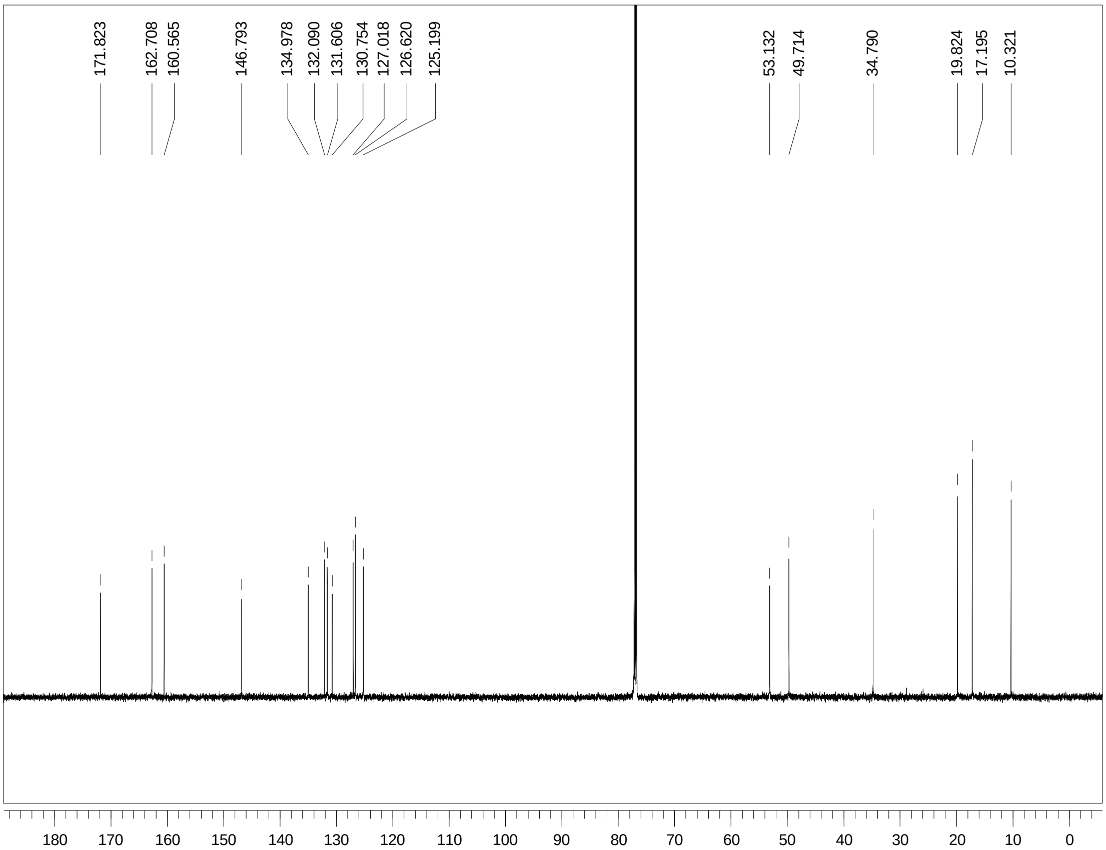
171.823
162.708
160.565
146.793
134.978
132.090
131.606
130.754
127.018
126.620
125.199
53.132
49.714
34.790
19.824
17.195
10.321
180 170 160 150 140 130 120 110 100 90 80 70 60 50 40 30 20 10 0
ppm (t1)

8. Supporting Information References

[1] G. N. Ramachandran, R. Chandrasekaran, K. D. Kopple, *Biopolymers* **1971**, *10*, 2113-2131.

[2] G. Haberhauer, C. Kallweit, C. Wölper, D. Bläser, *Angew. Chem.* **2013**, *125*, 8033-8036; *Angew. Chem., Int. Ed.* **2013**, *52*, 7879-7882.

[3] G. Haberhauer, F. Rominger, *Eur. J. Org. Chem.* **2003**, 3209-3218.

[4] M. Schnopp, S. Ernst, G. Haberhauer, *Eur. J. Org. Chem.* **2009**, 213-222.

[5] N. C. McMullen, F. R. Fronczek, T. Junk, *J. Heterocyclic Chem.* **2013**, *50*, 120-124.

[6] G. Haberhauer, F. Rominger, *Tetrahedron Lett.* **2002**, *43*, 6335-6338.

[7] G. Haberhauer, Á. Pintér, T. Oeser, F. Rominger, *Eur. J. Org. Chem.* **2007**, 1779-1792.

[8] G. Haberhauer, *Angew. Chem.* **2007**, *119*, 4476-4479; *Angew. Chem., Int. Ed.* **2007**, *46*, 4397-4399.

[9] B. Miehlich, A. Savin, H. Stoll, H. Preuss, *Chem. Phys. Lett.* **1989**, *157*, 200-206.

[10] A. D. Becke, *Phys. Rev. A* **1988**, *38*, 3098-3100.

[11] C. Lee, W. Yang, R. G. Parr, *Phys. Rev. B* **1988**, *37*, 785-789.

[12] S. Grimme, *J. Chem. Phys.* **2006**, *124*, 034108.

[13] S. Grimme, S. Ehrlich, L. Goerigk, *J. Comp. Chem.* **2011**, *32*, 1456-1465.

[14] J. Čížek, *Adv. Chem. Phys.* **1969**, *14*, 35-89.

[15] G. D. Purvis III, R. J. Bartlett, *J. Chem. Phys.* **1982**, *76*, 1910-1918.
